# Phenylpyridine-Based Borenium Salts as Lewis Acid Catalysts for Homocoupling of Diaryldiazomethanes

**DOI:** 10.1021/acs.inorgchem.5c04965

**Published:** 2026-01-05

**Authors:** Michaela Buziková, Jiří Schulz, Miroslava Litecká, Karel Škoch

**Affiliations:** a Institute of Inorganic Chemistry of Czech Academy of Sciences, Husinec-Řež 1001 250 68, Czech Republic; b Department of Inorganic Chemistry, Faculty of Science, 37740 Charles University, Hlavova 2030, Prague 128 40, Czech Republic

## Abstract

Cationic borenium salts have emerged as powerful Lewis acids, but their potential in carbon–carbon bond formation remains largely unexplored. Here, we report the synthesis of a family of sterically and electronically differentiated arylpyridine-based borenium species prepared by controlled hydride elimination and subsequent heteroatom or aryl substitution at boron. These compounds act as highly active catalysts for the homocoupling of diaryldiazomethanes to give tetraarylethylenes under remarkably mild conditions (0.1 mol % catalyst loading at room temperature). The majority of the prepared cationic Lewis acids outperformed neutral tris­(pentafluorophenyl)­borane, highlighting the advantage of cationic species in this transformation, with the sterically crowded mesityl-substituted borenium catalyst proving to be the most active. Control experiments and DFT calculations support a mechanism in which the borenium catalyst coordinates to the diazo substrate, promotes stepwise nitrogen extrusion and carbocation formation, and releases the tetraarylethylene product in a thermodynamically favorable pathway. This work reveals an unexploited avenue for the main-group catalysis of C–C bond-forming reactions and broadens the synthetic utility of borenium Lewis acids beyond their established reactivity profiles.

## Graphical abstract

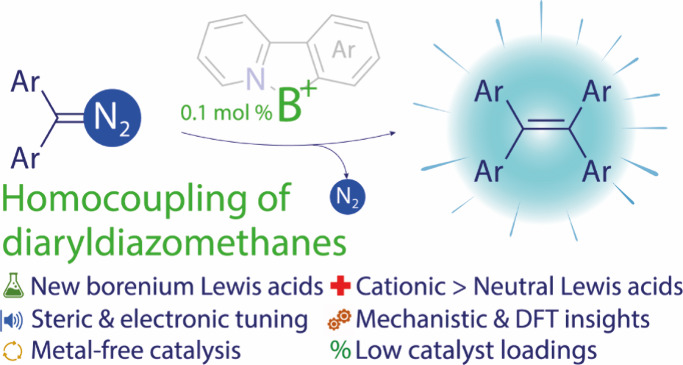

## Introduction

Tetraphenylethylenes (TPEs) represent a widely studied class of compounds due to their rich excited-state properties resulting from the specific molecular geometry of the extended π-system. These features contribute to their applications across various fields, spanning from material science^1^ to electron-transfer catalysis,^2^ biochemical sensing,^3^ or formation of supramolecular assemblies.^4^ TPEs represent an archetypal luminogen for aggregation-induced emission (AIE)^5^ and as such are valued also as fluorescent sensors^6^ or stimuli-response materials.^7^

Typical methods for preparation of substituted polyaromatic ethylenes include McMurry homocoupling,^8^ Peterson olefination,^9^ Barton–Kellogg reaction,^10^ Suzuki coupling,^11^ or Pd-mediated arylation of acetylenes.^12^ These are well-established methods, many of which allow for precise control of stereochemistry. However, they are either transition-metal mediated or require stoichiometric amounts of reactive reagents such as TiCl_3_ or alkyl lithiums. A notable gap in the field is the lack of methods utilizing main-group element catalysis.

Our interest lies in the group of highly Lewis acidic cationic boron species, known as the borenium salts.^13^ Recently, we reported the synthesis of simple arylpyridine-based C,N-boron chelates featuring weakly coordinating counterions, which exhibit remarkable Lewis acidity.^14^ Given their structural similarity to boroles, which are well known for undergoing insertion reactions of diazo compounds into the B–C bond,^15^ we examined the reactivity of phenylpyridine borane **2** featuring weakly coordinating triflimide counterion in a reaction with diphenyldiazomethane. Instead of the anticipated ring extension, we observed rapid consumption of the diazo compound and the formation of tetraphenylethene, while compound **2** remained unchanged throughout the process, apparently acting as a catalyst (Figure 1).

**Figure 1:**
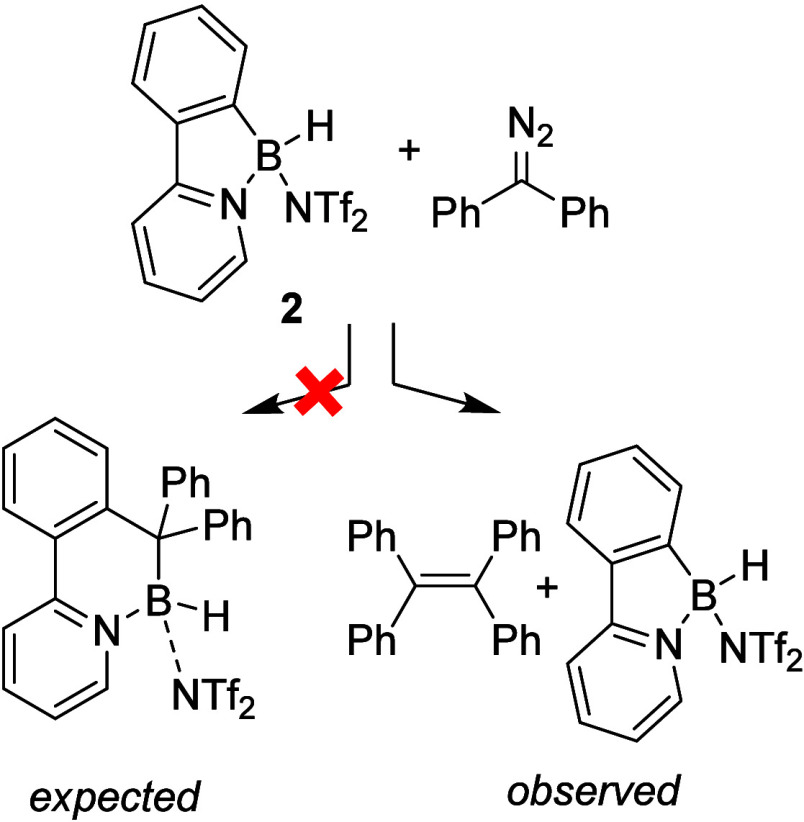
Serendipitous discovery of the borenium-mediated homocoupling of diaryldiazomethanes.

Borenium ions have recently emerged as exceptionally attractive catalysts^16^ for a wide range of transformations, including reductions of unsaturated bonds,^17^ C–H and C–C activation processes,^18^ olefin isomerizations,^19^ and diverse electrophile-mediated borylations of aromatic compounds.^20^ These reactions predominantly deliver C–H or C–B bonds. Only a few examples of C–C bond formation have been reported including a borenium-catalyzed Mukaiyama aldol-type reaction^21^ and an enantioselective Diels–Alder-type cyclization typically mediated by an acid-activated chiral oxaborolidine.^22^ Activation of α-carbonyl diazo compounds by neutral Lewis acids (typically tris­(pentafluorophenyl)­borane, BCF) and their subsequent transformation has been extensively investigated by Melen^23^ and others.^24^ In one of these studies, the formation of tetraphenylethylene arising from the homocoupling of diphenyldiazomethane was identified as a competing product during the substrate-scope investigation.^25^ Diazocompound (homo)­coupling reactions have also been achieved with late transition-metal systems, typically employing rhodium^26^ or ruthenium^27^ complexes characteristic of carbene transfer chemistry. Similar reactivity has been observed as stoichiometric outcomes in copper-^28^ or iron-mediated^29^ processes.

This study therefore introduces a distinct main-group catalytic approach and highlights the underexplored potential of borenium catalysts in promoting carbon–carbon bond-forming reactions and significantly expands their synthetic utility beyond the established reactivity profiles.

## Results and Discussion

The key precursor for the preparation of the cationic Lewis acid catalysts investigated in this work is borane **1**, which is readily accessible in a two-step synthesis starting from 2-phenylpyridine.^30^ The hydride elimination afforded the simplest Lewis acid catalyst in this study, boron-hydride-type borenium compound **2**. As this compound smoothly hydroborates olefins, we introduced cyclopentylborenium compound **3** into this study as a representative of alkyl- substituted cationic Lewis acid (Figure 2). Building on our previous studies of borenium **2** with azide nucleophiles,^31^ we extended its reactivity to oxygen- and sulfur-based nucleophiles, simultaneously taking advantage of the acidic character of aromatic alcohols and thiols to prepare sterically and electronically distinct species. Treatment of **2** with a stoichiometric amount of mesityl alcohol initially afforded a metastable adduct **4O**, in which the acidic phenolic proton and the boron hydride coexist. Upon slow synproportionation of these fragments, hydrogen gas was eliminated, leading to the formation of borenium ion **5O**, which was isolated after 10 days as the sole reaction product in an excellent yield of 91% (for experimental details and reaction monitoring, see the Supporting Information (SI)). Similar reactivity with the more acidic thiol analogue was expected; however, no reaction was observed when triflimide borane **2** was treated with mesityl thiol. Gratifyingly, when the *in situ* generated borenium **2′** (featuring a noncoordinating tetrakis­(pentafluorophenyl)­borate counterion) was used, the reaction proceeded significantly faster; intermediate **4S** was converted to borenium **5S** within hours, and compound **5S** was ultimately isolated in 89% yield, albeit contaminated with a small amount of some trityl-based impurity (see the SI). In both cases, formation of borenium salts was evidenced by NMR, as these compounds feature a shift typical for tricoordinate boron species (^11^B NMR δ_B_ = 31.7 for **5O** and 53.5 for **5S**). Single crystals were obtained, and molecular structures for **5O** and **5S** were determined (see Figure 4; for further details on synthesis, characterization data, and discussion on molecular structures, see the SI).

**Figure 2:**
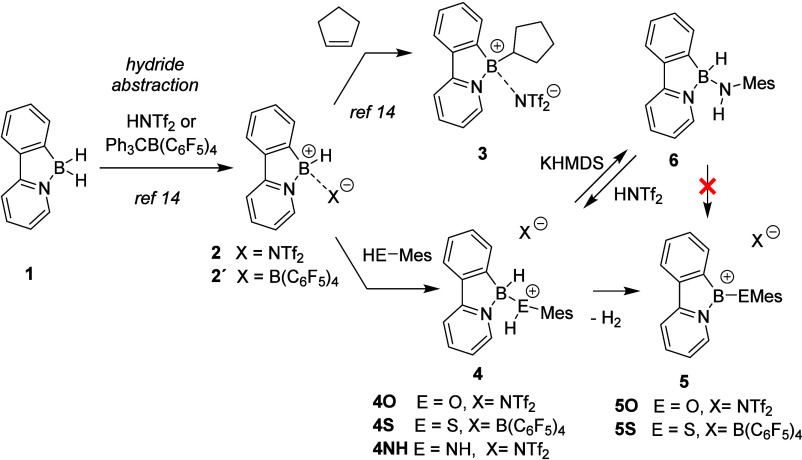
Preparation of Lewis acids **2**, **3**, **5O**, and **5S**.

For completeness, compound **2** was also reacted with a mesityl amine. In this case, adduct **4NH** was formed, which did not undergo dihydrogen elimination even under forcing conditions. Treatment with a base afforded a boron amine species **6** (a structurally related motif was recently reported via Rh-mediated reaction of boranes with nitrenes).^32^ Attempts to abstract the hydride and stabilize the corresponding borenium salt featuring the NH-Mes moiety were not successful. The reaction with trityl borate did not proceed selectively and resulted in a complex mixture of products, whereas the reaction with triflimic acid led only to protonation of the nitrogen atom and regeneration of compound **4NH** (for details, see the SI).

For the introduction of a bulky aromatic carbon substituent (mesitylene), we have adapted a procedure previously reported for the reaction of organometallic reagents with borenium-type species (Figure 3).^33^ First, borenium **2** was treated with 0.5 equiv. of iodine and the *in situ* generated boron iodide was then reacted with MesMgBr. The obtained mesityl boron hydride **7** is stable enough to be purified by column chromatography, and it was isolated in a moderate yield of 58% (for characterization and crystal structure of **7**, see the SI). Abstraction of the second hydride by the addition of trityl borate to **7** provided sterically encumbered borenium **8**, which was isolated as a yellow solid in an excellent yield of 82% (for reaction details, see the SI). In this case, the formation of tricoordinate borenium species was also manifested by ^11^B NMR, featuring a broad signal at δ_B_ = 61.3 ppm (alongside a sharp singlet at δ_B_ = −17.7 ppm corresponding to the B­(C_6_F_5_)_4_ counterion), and its structure was unambiguously confirmed by single-crystal X-ray diffraction analysis (see Figures 3 and 4; for details on synthesis and characterization data, see the SI).

**Figure 3:**
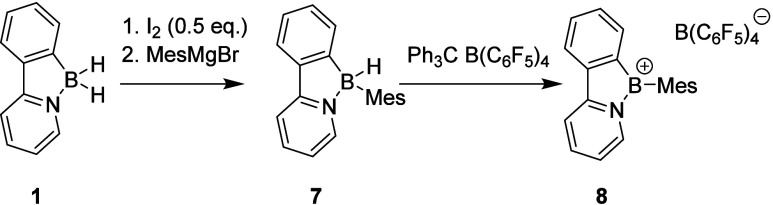
Preparation of mesitylborenium **8**.

**Figure 4:**
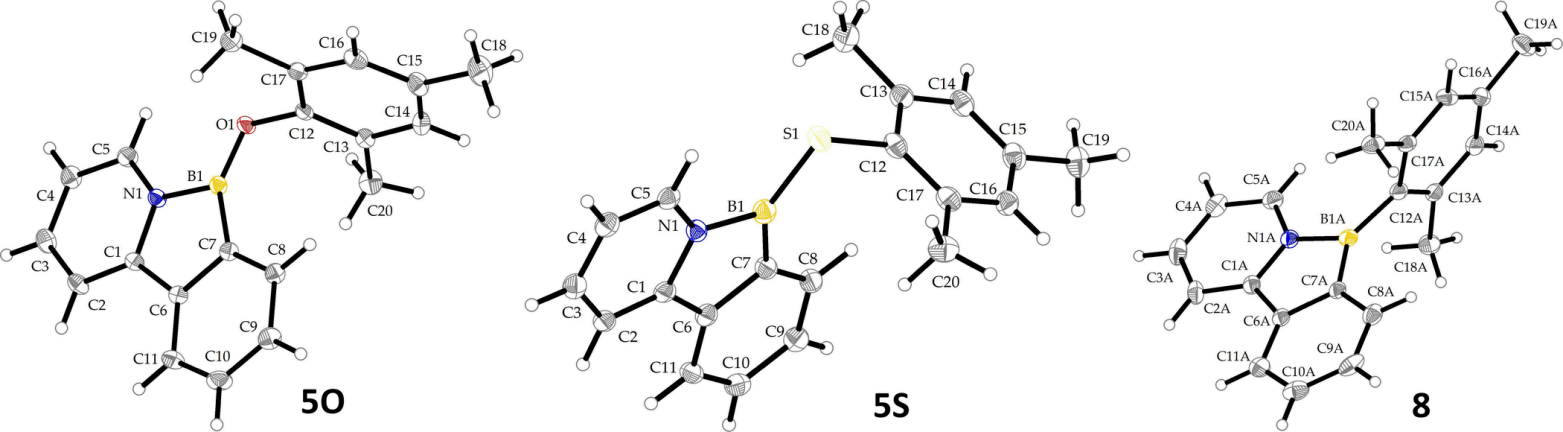
Crystal structure of the cationic part of compounds **5O**, **5S**, and **8** with thermal ellipsoids shown at the 30% probability level. For compound **8**, one of the two independent cations is shown. Selected bond lengths and angles for **5O**: B1–O1 1.332(2), B1–N1 1.516(2), B1–C7 1.547(2), N1–B1–C7 104.6(1), B1–O1–C12 119.7(1), and ∑B1_ONC_ 399.95; **5S**: B1–S1 1.769(5), B1–N1 1.523(6), B1–C7 1.540(6), N1–B1–C7 104.3(3), B1–S1–C12 101.4(2), and ∑B1_SNC_ 360; **8** (two independent units A/B): B1–C12 1.538(5)/1.544(5), B1–N1 1.537(5)/1.531(4), B1–C7 1.532(5)/1.533(5), N1–B1–C7 102.1(3)/102.6(3), and ∑B1_NCC_ 360/360.

Fluoride ion affinity (FIA) is a well-established tool for the quantitative assessment of Lewis acidity.^34^ To obtain a better understanding of the Lewis acidity trends within the prepared series of borenium compounds, FIA values were calculated at the PW6B95­(D3)/def2-QZVPP//PBE0­(D3)/def2-TZVP level of theory. Calculations were carried out both in vacuo and within a dichloromethane solvent continuum, as cationic Lewis acids may otherwise display substantially exaggerated values arising from charge separation.^35^ This effect was clearly reflected in the FIA values determined for the borenium cations derived from compounds **2**, **3**, **5O**, **5S**, and **8** (see Table 1). The computed energies confirm that the derived cations are indeed strong Lewis acids, with compounds **2** and **3** appearing as the strongest in this series, at least according to the FIA parameter.

**Table 1:** Fluoride Affinities FIA (in kJ·mol^–1^ at 298.15 K) Calculated In Vacuo and in Dichloromethane at the PW6B95­(D3)/def2-QZVPP//PBE0­(D3)/def2-TZVP Level of Theory and Determined the Buried Volumes (*V*_Bur_) of Derived Borenium Cations^a^

compound	FIA (vacuo)	FIA (CH_2_Cl_2_)	*V*_Bur_ (%)
**2**	776	317	42.2
**3**	744	285	49.0
**5O**	708	248	55.3
**5S**	722	263	55.5
**8**	722	262	56.5

a Solvent effects have been approximated using the PCM model.

Having already optimized the structures required for the FIA calculations, we also determined the corresponding buried volumes of the individual borenium cations. Although this parameter was originally developed for describing properties of Lewis bases,^36^ it can be effectively applied to evaluate steric crowding of Lewis acids as well.^37^ The calculated values are summarized in Table 1, and the individual topographic steric maps are provided in the Supporting Information.

### Catalyst Screening

Our initial observation of tetraphenylethylene formation was done in a stoichiometric reaction of **2** with diphenyldiazomethane **9a** with direct monitoring of the reaction by ^1^H NMR spectroscopy. Equal immediate gas evolution and rapid discoloration of the reaction mixture were observed when 10 mol % **2** was employed. In this case, tetraphenylethylene **10a** was isolated in 81% yield, alongside an additional species (7% yield), identified as bis­(diphenylmethylene)­hydrazine **11a**. At 1 mol % catalyst loading, formation of azine **11a** was effectively suppressed and quantitative formation of tetraphenylethylene **10a** was observed by NMR after 60 min. The product **10a** was isolated in 92% yield by simple filtration through a silica gel plug.

Given the rapid nature of the reaction, the system was further investigated using bis­(*p*-tolyl)­diazomethane **9b**, which offers a more diagnostic NMR signal pattern, while the discoloration of its deep-purple color also enables monitoring of the reaction rate at a lower concentration by UV–vis spectroscopy. This approach allowed us to capture the subtle differences in catalyst performance.

First, we examined the effect of the solvent on the reaction. The study was carried out under rather competitive conditions, when bis­(*p*-tolyl)­diazomethane **9b** was treated with 0.1 mol % of the simplest representative in our borenium series, compound **2**, in the corresponding solvent, and the reaction progress was directly monitored by UV–vis spectroscopy (for experimental details, see the SI). Plotting the time dependence of the natural logarithm of absorbance at 535 nm (the absorption maximum of the substrate) revealed that the reaction follows first-order kinetics. Individual solvents were evaluated in terms of the observed reaction rate constants *k*_obs_ (see Figure 5 and Table 2). The highest activity was observed in halogenated solvents (chloroform or dichloromethane), which represent the best compromise between solubility and stability of the acidic centers. During these experiments, the reaction mixture essentially decolorized within the reaction time frame (90 min), and the absorbance at the maximum decreased to <10% of its initial value indicating conversion of the starting material exceeding 90%. In contrast, the reaction proceeded significantly slower in ethereal solvents, consistent with the competitive coordination of the solvent and substrate to the Lewis acidic center, thereby suppressing catalytic activity. For subsequent studies, chloroform was selected due to its availability, capacity to heat the reaction mixture, and the possibility to directly monitor the reaction progress by NMR spectroscopy.

**Figure 5:**
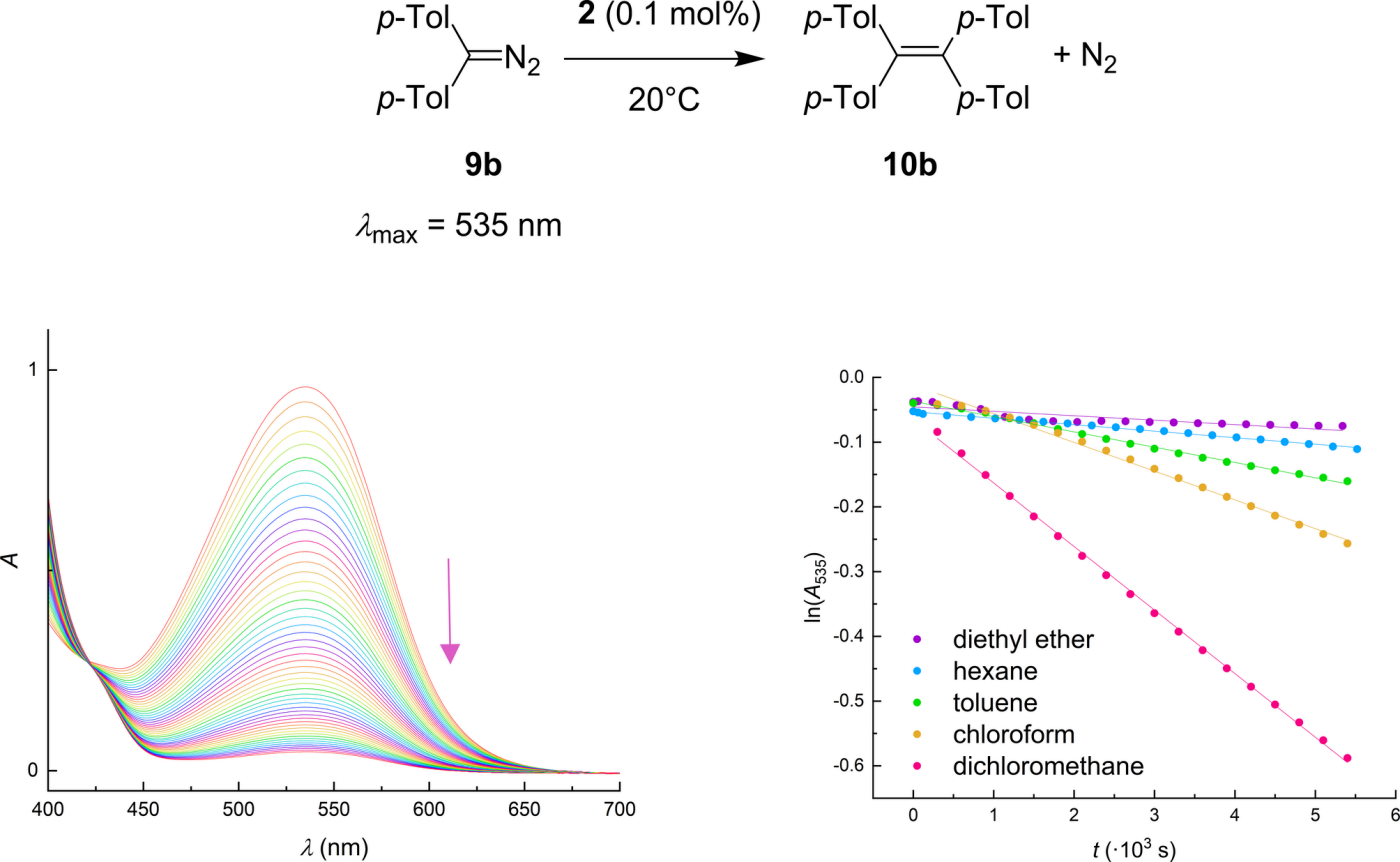
Example of a course of the homocoupling reaction monitored by UV–vis spectroscopy, showing the decrease in absorbance at 535 nm corresponding to the absorption maximum of **9b** (left); comparison of kinetic profiles in different solvents used in this study (right). Reaction conditions: [**9b**] = 10 mM, [**2**] = 0.01 mM, *T* = 20 °C, *t* = 0–90 min (right).

**Table 2:** Observed Rate Constants (*k*_obs_) of the Homocoupling Reaction in Various Solvents Determined by UV–Vis Reaction Monitoring under the Same Conditions as in Figure 5

solvent	*k*_obs_ (· 10^–5^ s^–1^)
hexane	1.00
diethyl ether	0.682
dichloromethane	9.82
chloroform	3.91
toluene	2.36

In the following step, we investigated the activity of individual borenium salts **2**, **3**, **5O**, and **8** and electronically differentiated borenium-type compounds **12**–**14** (Figure 6), which we reported previously.^14^ Tris­(pentafluorophenyl)­borane B­(C_6_F_5_)_3_ was also included as a benchmark Lewis acid for comparison. Catalytic performances of tested compounds expressed as observed rate constants (*k*_obs_) are summarized in Table 3.

**Figure 6:**
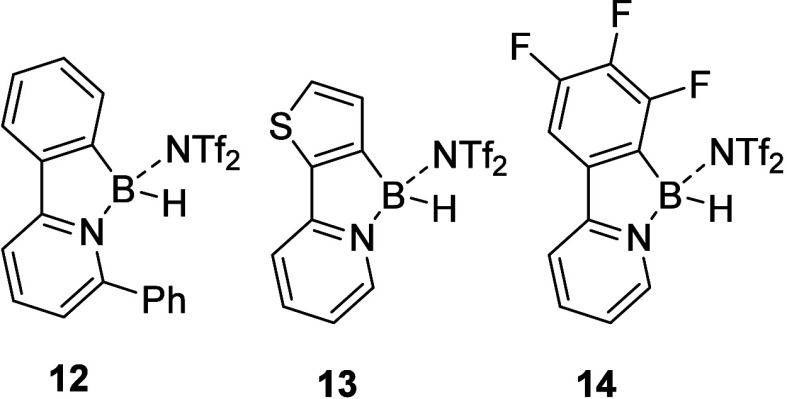
Sterically and electronically differentiated borenium-type compounds were included in this study.

**Table 3:** Observed Rate Constants (*k*_obs_) for Individual Catalysts Determined by UV–Vis Reaction Monitoring, Employing 0.1 mol % of the Corresponding Catalyst^a^

catalyst	*k*_obs_ (˙· 10^–5^ s^–1^)
**2**	3.91
**3**	5.17
**5O**	10.9
**8**	15.4
**12**	2.68
**13**	7.48
**14**	n.a.
B(C_6_F_5_)_3_	1.37

a Conditions: [**9b**] = 10 mM, [**2**] = 0.01 mM, *T* = 20 °C, *t* = 0–5 h, n.a. = not available

Modification of the arylpyridine scaffold of the borenium catalyst indicated a trend in which the reaction proceeded more efficiently with electronically richer phenylthiophene-based borenium species **13** than with the strongly Lewis acidic trifluorophenylpyridine **14**, which proved to be essentially inactive. A clearer trend emerged upon direct substitution and introduction of steric bulk at the boron center, as exemplified by increasing efficiency of cyclopentylborenium **3**, mesityloxyborenium **5O**, and most notably mesitylborenium compound **8**. The best performance was observed for strictly tricoordinated cations **5O** and **8**, indicating that the energy required for counterion dissociation can also play a significant role. Neutral Lewis acid B­(C_6_F_5_)_3_ also displayed considerable catalytic activity; however, under the conditions employed (0.1 mol % catalyst loading, r.t.), its performance was consistently inferior to those of the cationic Lewis acids (with the exception of compound **14**). These results suggest that the efficiency of the catalyst is not dictated simply by maximizing the Lewis acidity but rather reflects a delicate balance between the acidity and the steric crowding at the active site.

In a short substrate study (Figure 7), we included synthetically accessible diaryldiazomethanes bearing both electron-donating and electron-withdrawing substituents. As benchmark conditions, we employed 1 mol % of catalyst **8** in CHCl_3_ as the solvent (see Figure 7). The reaction proceeded smoothly with unsubstituted diphenyldiazomethane **9a**, as well as with diaryldiazomethanes carrying electron-donating groups (*p*-methyl **9b**, *p*-methoxy **9c**) and with cyclic diazomethanes derived from fluorene or dibenzosuberone, providing the corresponding tetraethylenes **10** in excellent yields. The electron-deficient bis­(*p*-bromophenyl)­diazomethane **9d** required heating to 60 °C but also afforded tetra­(bromophenyl)­ethylene **10d** in an excellent yield of 87%. Compounds bearing Lewis-basic substituents (e.g., cyano or nitro) did not undergo any reaction, while reactions with trifluoromethyl-substituted diaryldiazomethane ((3,5-CF_3_)_2_C_6_H_3_)_2_CN_2_ gave product mixtures, likely due to competing processes (the Lewis acid-mediated defluorination of ArCF_3_ groups is well documented).^38^ reference 38 here We have also included phenyl-*tert*-butyldiazomethane **9g** as a synthetically available and isolable alkyl–aryl diazomethane representative. This provided the corresponding azine **11g** as the sole product. Notably, the reaction does not proceed with α-carbonyl diazomethanes (methyl phenyldiazoacetate), as it generates a less nucleophilic carbocation according to Mayr’s nucleophilicity scale.^39^

**Figure 7:**
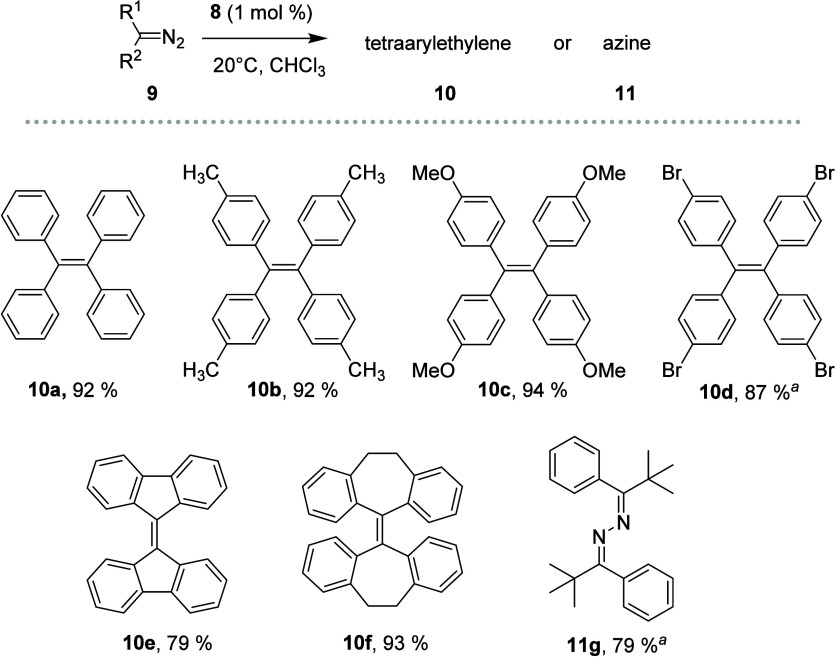
Substrate scope with isolated yields. ^a^Reaction conducted at 60 °C.

### Investigation of the Reaction Mechanism

Based on the obtained experimental data, we attempt to establish a reaction mechanism. Diaryldiazomethane possesses resonance-stabilized structures that allow it to act as either an N- or C-nucleophile. In a previous study, Stephan reported that coordination of Lewis acid B­(C_6_F_5_)_3_ to the nitrogen atom (as an N-nucleophile) affords a more stable isomer and even determined its structure, by isolation at low temperatures. He both experimentally and computationally evidenced that its rearrangement and coordination to carbon is thermodynamically accessible (19.4 kcal·mol^–1^) and it is readily followed by nitrogen elimination, yielding the thermodynamically stable B­(C_6_F_5_)_3_-diphenylcarbene adduct.^40^ We assumed a similar reaction pathway in our system. Borenium catalyst (A) initially coordinates to the carbon atom of diaryldiazomethane (intermediate B), followed by nitrogen elimination to form a trityl-like cation (C). This intermediate subsequently reacts with a second molecule of diaryldiazomethane acting again as a C-nucleophile to form adduct D. Further elimination of nitrogen (intermediate E), followed by the release of the borenium cation, leads to the formation of tetraphenylethene. The proposed mechanism is depicted in Figure 8.

**Figure 8:**
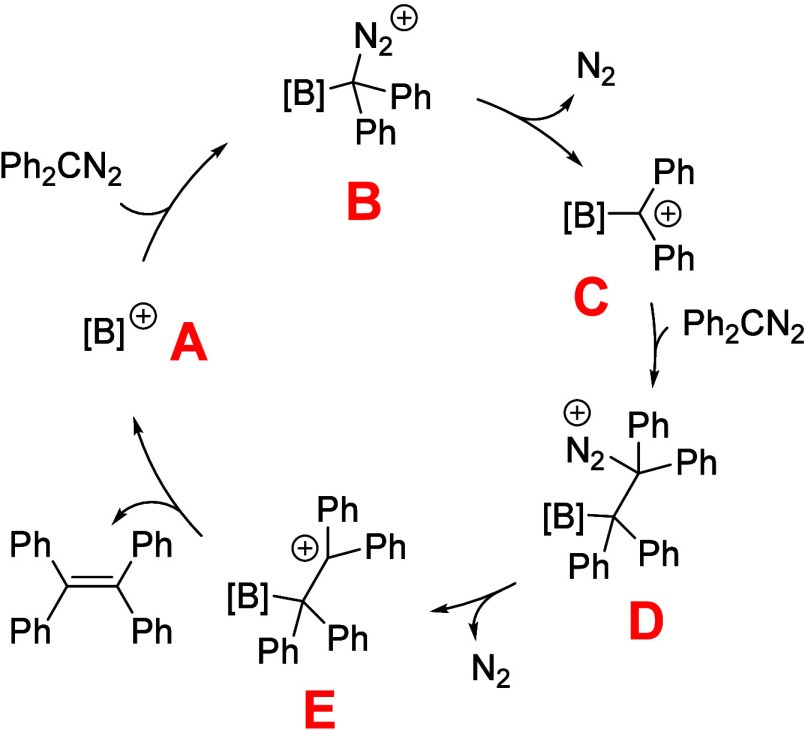
Proposed reaction mechanism for borenium-mediated homocouping of diaryldiazomethanes ([B]^+^ denotes a borenium cation as catalyst; A–E represent individual reaction steps in the reaction sequence).

In some cases, we observed the formation of minor amounts of bis-di­(*p*-tolylmethylene)­hydrazine **11b**. In a control experiment, azine **11b** was treated with 1 mol % of **2**, but no reaction occurred, suggesting that the azine is not an intermediate in the catalytic cascade but an off-cycle side product. We rationalize its formation as the result of an N-nucleophilic attack of diaryldiazomethane during the C → D step. When the reaction was carried out under control conditions in the presence of an olefin (styrene or cyclopentene), no traces of corresponding cyclopropene products were detected, further supporting the hypothesis of a free carbene intermediate not being involved in the reaction sequence.

In the mixed experiments, bis­(*p*-tolyl)­diazomethane **9b** was treated with an equimolar amount of bis­(*p*-methoxyphenyl)­diazomethane **9c** or bis­(*p*-bromophenyl)­diazomethane **9d** in the presence of 2 mol % **8** as the most efficient catalyst. In the **9b**/**9c** experiment, only a small amount (21%) of mixed tetraarylethylene **10bc** was obtained, whereas in the case of **9b/9d**, no cross-coupling product was observed at all. Majority of the material was identified as the product of homocoupling in both cases. This further supports our proposed mechanism, as the formation of both proposed intermediates B and D is governed by the same parameter: the nucleophilicity of the substrate. For further details on experimental investigation on reaction mechanism, see the SI.

The proposed reaction mechanism was also investigated using computational methods using the PBE0 ^41^ density functional in conjunction with the def2-TZVP^42^ basis set with added Grimme’s D3 dispersion correction.^43^ Solvent effects (dichloromethane) have been approximated using the PCM model^44^ (see Figure 9). DFT calculations indicate that borenium-NTf_2_ forms a strongly associated ion pair in solution, with dissociation to a free borenium cation being markedly endergonic (Δ*G* ≈ +22.4 kcal mol^–1^). Replacement of NTf_2_^–^ by diphenyldiazomethane to generate the corresponding borenium–substrate complex is significantly less endergonic (Δ*G* ≈ +8.6 kcal mol^–1^), which implies that although the catalytically active borenium–substrate species would only be a minor component of the equilibrium mixture, its steady-state concentration is still sufficient for the observed catalytic reaction. Coordination of the borenium cation via a nitrogen atom is thermodynamically preferred (by 5.6 kcal·mol^–1^). Extrusion of the nitrogen molecule after the isomerization to a carbon-bound isomer is a virtually barrierless (1.0 kcal·mol^–1^), highly exothermic process (−31.1 kcal·mol^–1^, relative to the nitrogen-bound isomer). The resulting carbocation (intermediate C) can then react with another molecule of the diazo compound. The activation energy for the reaction in which the diazo compound acts as a N-nucleophile is much lower (13.0 kcal·mol^–1^) than when it acts as a C-nucleophile (25.2 kcal·mol^–1^). However, in the latter case, an unstable intermediate is formed that can release another nitrogen molecule with virtually no barrier, forming an alkene. This second reaction channel is therefore much more thermodynamically favorable (by ca. 43 kcal·mol^–1^) and irreversible due to the extrusion of the nitrogen molecule. Consequently, the alkene can be described as a thermodynamic product, while the diazine would be a kinetic product. It is also worth mentioning that diazine may form a very stable N-bound adduct with a cationic borenium catalyst. The formation of this adduct may be one of the factors contributing to the preference of the red reaction pathway, because it leads to a notable difference in the relative stability of the product–catalyst complexes toward the substitution by a new substrate molecule (see Figure 9). In the case of the blue reaction pathway, displacement of the N-bound product by an incoming diazo substrate is calculated to be endergonic (6.8 kcal·mol^–1^), indicating that the product remains strongly coordinated and may inhibit a further catalytic turnover. In contrast, for the red pathway, product substitution by a substrate molecule is exergonic for all considered adducts of the borenium catalyst with tetraphenylethylene, suggesting that this reaction channel enables more efficient catalyst cycling. This distinction further implies that the red reaction pathway, although kinetically less accessible, may ultimately be more favorable.

**Figure 9:**
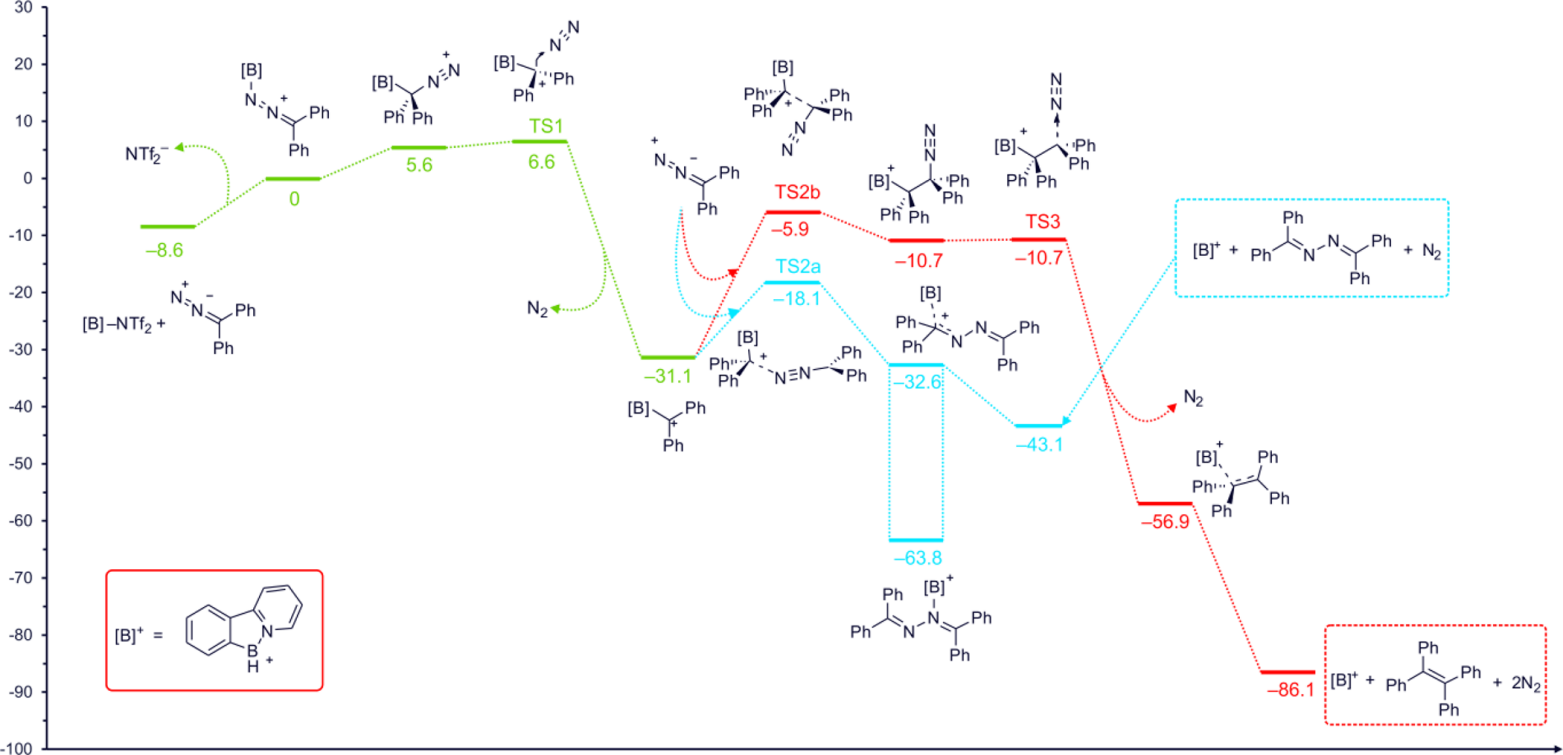
Energy profile calculated for the reaction of diphenyldiazomethane with the cationic borenium catalyst, showing the initial N_2_ extrusion and divergence into two consequent reaction pathways. The blue pathway proceeds via a lower barrier and furnishes the kinetic product, while the red pathway involves higher barriers but leads to the thermodynamically most stable species. The relative free energies (Δ*G*) are given in kcal·mol^–1^ relative to those of the N-bound adduct. Calculations were performed at the PBE0­(D3)/def2-TZVP level of theory with the solvent effect (CH_2_Cl_2_) approximated by the PCM solvation model.

## Conclusions

In conclusion, we report the synthesis of novel borenium-type Lewis acids and their application as catalysts in a homocoupling reaction of diaryldiazomethanes, leading to the corresponding tetraarylethylenes. The reaction proceeds efficiently even with very low catalyst loadings (0.1 mol %) at room temperature. Reported series of simple arylpyridine-based borenium Lewis acids outperform the traditional benchmark neutral Lewis acid tris­(pentafluorophenyl)­borane, which indicates that the use of cationic Lewis acids is advantageous for this type of reaction. Through control experiments and computational studies, we propose a reaction mechanism involving a stepwise electrophilic attack of the Lewis acid on the diazomethane substrate, further highlighting the key role of the cationic nature of the catalyst. This work reveals the untapped potential of activating diazo compounds with borenium species and demonstrates the promise of exploiting this combination of highly reactive intermediates for novel C–C bond-forming transformations.

## Experimental Methods

Experiments were performed under a dry argon atmosphere using standard Schlenk-type glassware and/or an argon filled glovebox, unless stated otherwise. Reaction solvents hexane, toluene, and tetrahydrofuran were dried by refluxing over sodium metal in the presence of benzophenone ketyl radical under an argon atmosphere. Dichloromethane was purified by distillation from CaH_2_ in argon atmosphere. Anhydrous solvents were stored over activated 4 Å molecular sieves. All chemicals were purchased from commercially available resources (Sigma-Aldrich, BLD-Pharm, TCI, and ABCR) and used as received. Compounds **1**, **2**, **3**, **12**, **13**, and **14** were prepared according to a reported procedure.^14^ Additional experimental details, methods, and materials including detailed reports on synthesis and characterizations of all prepared compounds is provided in the Supporting Information (SI).

*CAUTION: The diazo compounds investigated in this study are potentially unstable and may be prone to explosive decomposition, although no such incidents were observed during our experiments. Likewise, certain precursors (such as hydrazine or mercury oxide) may present additional toxicity hazards. All experimental work should therefore be conducted following appropriate safety protocols and established laboratory standards.*

NMR spectra were recorded on a JEOL Delta 600 spectrometer at 20 °C. ^1^H and ^13^C NMR chemical shifts (δ in ppm) are referenced to the residue solvent signal. ^11^B, ^19^F, and ^31^P are referenced according to the primary reference for the unified chemical shift scale following the IUPAC recommendation. Elemental analysis was performed on a FlashSmart Elemental Analyzer. Mass spectrometry measurements were performed on an Orbitrap Exploris 120 instrument using electrospray ionization. Infrared spectra were collected on a Nexus 670 FT-IR spectrometer. X-ray diffraction analysis was performed with a Rigaku XtaLAB Synergy S diffractometer equipped using Cu (Cu/Kα radiation; λ = 1.54184 Å) with a microfocus X-ray source and a Hybrid Pixel Array Detector (HyPix-6000HE). An Oxford Cryosystems (Cryostream 800) cooling device was used for data collection at a temperature of 100 K.

### Synthesis of **5O**

To a stirring solution of **2** (292 mg, 0.65 mmol) in 5 mL of DCM was added mesityl alcohol (89.1 mg, 0.65 mmol, 1 equiv) in DCM (5 mL). The mixture was stirred at room temperature for 10 days to ensure the reaction completion. Then, the solvent was evaporated under reduced pressure, the residue redissolved in a minimum amount of DCM (ca. 1 mL), layered with hexane (ca. 8 mL), and left overnight for crystallization. Obtained crystals were filtered, washed with hexane, and dried, providing **5O** in the form of yellow crystalline solid (345 mg, yield: 91%). Elemental analysis calculated for C_22_H_19_BF_6_N_2_O_5_S_2_ (580.32): C 45.53, H 3.30, N 4.83; found C 44.69, H 3.42, N 4.38; NMR (CDCl_3_, 20 °C): ^1^H (600 MHz): δ 2.17 (s, 6H, *o*–C*H*_3_), 2.34 (s, 3H, *p*–C*H*_3_), 6.85 (d, ^3^ *J*_HH_ = 7.2 Hz), 6.96 (s, 2H, C*H* mesityl), 7.37 (t, ^3^ *J*_HH_ = 7.5 Hz, 1H), 7.66 (td, ^3^ *J*_HH_ = 7.7, ^4^ *J*_HH_ = 1.2 Hz, 1H), 7.89 (ddd, ^3^ *J*_HH_ = 7.3, 5.7 Hz; ^4^ *J*_HH_ = 0.97 Hz, 1H), 7.94 (d, ^3^ *J*_HH_ = 7.7 Hz, 1H), 8.27 (d, ^3^ *J*_HH_ = 8.0 Hz, 1H), 8.63 (td, ^3^ *J*_HH_ = 7.9 Hz; ^4^ *J*_HH_ = 1.5 Hz, 1H), 8.87 (m, 1H); ^11^B (193 MHz) δ 31.5; ^11^B­{^1^H} (193 MHz) δ 31.7 (s); ^19^F (565 MHz) δ – 78.6 (s); ^13^C­{^1^H} (151 MHz) δ 16.6, 20.9, 119.8 (q, ^1^ *J*_FC_ = 321 Hz), 121.3, 124.7, 126.6, 127.3, 130.1, 134.0, 134.5, 135.5, 136.3, 141.7, 143.1, 148.3, 152.5, 158.2; signal of C–B was not observed; HRMS (ESI): *m*/*z* [M]^+^ calculated for C_20_H_19_NBO: 300.1554; found: 300.1551; HRMS (ESI): *m*/*z* [M]^−^ calculated for C_2_F_6_NO_4_S_2_: 279.9178; found: 279.9169; IR (KBr): 3120 (m), 3095 (m), 3069 (m), 2958 (m), 2925 (m), 2866 (w), 1631 (s), 1609 (m), 1568 (w), 1528 (m), 1508 (s), 1486 (m), 1437 (s), 1352 (s), 1275 (m), 1198 (s, NTf_2_), 1136 (s), 1096 (m), 1056 (s), 957 (w), 900 (m), 852 (m), 791 (m), 779 (m), 763 (m), 741 (s), 730 (s), 653 (m), 619 (s), 575 (s), 514 (s), 430 (w) cm^–1^.

### Synthesis of **4NH**

To a stirring solution of **2** (394 mg, 0.88 mmol) in DCM (10 mL) was added mesityl amine (119.4 mg, 1 equiv) in DCM (5 mL), and the solution was left stirring at room temperature for 1 h. Then, the solvent was evaporated and the residue triturated with hexane (3 × 3 mL). Upon solvent evaporation and drying *in vacuo*, **4NH** was obtained in the form of white powder (490 mg, yield: 95%). Elemental analysis calculated for C_22_H_22_BF_6_N_3_O_4_S_2_ (581.36): C 45.45, H 3.81, N 7.23; found C 44.91, H 3.27, N 7.07; NMR (CDCl_3_, 20 °C): ^1^H (600 MHz): δ 1.60 (br s, 3H), 2.33 (s, 3H), 2.54 (br s, 3H), 3.95 (br s, 1H, B-*H*), 6.24 (br m, 1H, N*H*), 6.66 (d, ^3^ *J*_HH_ = 7.3 Hz, 1H), 6.91 (br s, 2H), 7.28 (t, ^3^ *J*_HH_ = 7.3 Hz, 1H), 7.39 (br d, ^2^ *J*_HH_ = 13.4 Hz, 1H, N*H*), 7.44 (td, ^3^ *J*_HH_ = 7.6 Hz, ^4^ *J*_HH_ = 1.1 Hz, 1H), 7.65 (ddd, ^3^ *J*_HH_ = 7.2, 5.6 Hz; ^4^ *J*_HH_ = 1.2 Hz, 1H), 7.85 (d, ^3^ *J*_HH_ = 7.7 Hz, 1H), 8.07 (d, ^3^ *J*_HH_ = 8.1 Hz, 1H), 8.27 (td, ^3^ *J*_HH_ = 7.8, ^4^ *J*_HH_ = 1.4 Hz, 1H), 8.85 (d, ^3^ *J*_HH_ = 5.8 Hz, 1H); ^13^C­{^1^H} (151 MHz): δ 17.5, 20.8, 118.9, 119.4 (q, ^3^ *J*_FC_ = 321 Hz), 122.2, 123.9, 129.1, 130.3, 130.4, 131.7, 132.1, 137.3, 137.9, 144.5, 145.4, 146.8 (*C*-B), 158.4; ^11^B (193 MHz): δ – 2.8, ^11^B­{^1^H} (193 MHz) δ – 2.7 (s); ^19^F (565 MHz): δ – 78.9 (s); HRMS (ESI): *m*/*z* [M]^+^ calculated for C_20_H_22_BN_2_: 301.1871; found: 301.1902; HRMS (ESI): *m*/*z* [M]^−^ calculated for C_2_F_6_NO_4_S_2_: 279.9178; found: 279.9167; IR (KBr): 3226 (m, NH), 3145 (m, NH), 3070 (w), 2926 (w), 2866 (w), 2466 (m, BH), 1628(s), 1595 (m), 1541 (w), 1491 (s), 1452 (m), 1342 (s), 1231 (s), 1196 (br s, NTf_2_), 1134 (s), 1108 (s), 1080 (m), 1059 (s), 1010 (w), 939 (w), 859 (m), 791 (w), 763 (m), 740 (m), 663 (m), 654 (m), 615 (m), 601 (s), 572 (s), 509 (m), 461 (w) cm^–1^.

### Synthesis of **6**

Compound **4NH** (257 mg, 0.44 mmol) was dissolved in anhydrous toluene (10 mL), and potassium bis­(trimethylsilyl)­amide (KHMDS, 88.2 mg, 1 equiv) was added. The mixture was stirred at room temperature for 2 h. Then, the solvent was evaporated and the residue redissolved in chloroform (10 mL). Precipitated salts were removed by filtration through a PTFE microfilter, and the obtained solution concentrated *in vacuo*. After drying, **6** was obtained in the form of yellow powder (130 mg, yield: 98%). NMR (CD_2_Cl_2_, 20 °C): ^1^H (600 MHz): δ 2.11 (s, 6H), 2.21 (s, 3H), 4.02 (d, ^3^ *J*_HH_ = 133.8 Hz, 1H, B-*H*), 6.76 (s, 2H), 7.27 (ddd, ^3^ *J*_HH_ = 7.1, 5.6 Hz; ^4^ *J*_HH_ = 1.3 Hz, 1H), 7.36 (td, ^3^ *J*_HH_ = 7.5 Hz, ^4^ *J*_HH_ = 1.2 Hz, 1H), 7.43 (td, ^3^ *J*_HH_ = 7.2 Hz, ^4^ *J*_HH_ = 1.1 Hz, 1H), 7.70 (d, ^3^ *J*_HH_ = 7.2 Hz, 1H), 7.85 (dt, ^3^ *J*_HH_ = 7.6 Hz, ^4^ *J*_HH_ = 1.0 Hz, 1H), 7.95 (dt, ^3^ *J*_HH_ = 8.1 Hz, ^4^ *J*_HH_ = 1.1 Hz, 1H), 7.99 (td, ^3^ *J*_HH_ = 7.7 Hz, ^4^ *J*_HH_ = 1.5 Hz, 1H), 8.16 (dt, ^3^ *J*_HH_ = 5.7 Hz, ^4^ *J*_HH_ = 1.3 Hz, 1H); ^13^C­{^1^H} (151 MHz): δ 19.8, 20.6, 118.2, 121.5, 122.3, 126.8, 127.6, 128.5, 129.4, 130.9, 136.9, 140.9, 143.6, 146.6, 146.5, 156.1, 158.1 (br, *C*-B); ^11^B (193 MHz): δ 0.2 (d, ^1^ *J*_BH_ ≈ 100 Hz); ^11^B­{^1^H} (193 MHz): δ 0.2 (s); IR (KBr): 3363 (m, NH), 3042 (w), 2908 (m), 2854 (m), 2734 (w), 2379 (s, B–H), 1615 (s), 1570 (w), 1561 (w), 1481 (s), 1439 (m), 1386 (m), 1326 (m), 1298 (m), 1287 (w), 1267 (w), 1244 (m), 1230 (m), 1172 (m), 1160 (w), 1111 (m), 1081 (w), 1064 (m), 1044 (w), 1007 (m), 976 (w), 94 (w), 927 (w), 859 (m), 824 (w), 766 (s), 741 (s), 716 (w), 689 (m), 654 (m), 605 (m), 585 (m), 568 (m), 507 (w), 487 (w), 457 (w), 419 (w) cm^–1^.

### Synthesis of **7**

To a stirred solution of **1** (334 mg, 2.0 mmol) in anhydrous toluene (5 mL), a solution of iodine (254 mg, 1 mmol, 0.5 equiv) in 10 mL of toluene was added dropwise. Upon addition, release of hydrogen gas was observed, together with the discoloration of iodine in solution. After addition, the mixture was stirred for 30 min at room temperature. The yellow suspension was then cooled to −78 °C using ethanol/liquid nitrogen bath. After cooling, solution of MesMgBr (1.0 m in Et_2_O, 2.2 mL, 1.1 equiv) was added dropwise and the mixture was cooled for additional 30 min. The solution was then allowed to warm to room temperature and stirred overnight. The next day, the reaction was quenched with methanol (2 mL), volatiles were removed *in vacuo*, and the mixture was purified by column chromatography on silica gel using DCM/hexane 1/1 (v/v) as a mobile phase. Combined fractions containing the product were evaporated, yielding **7** in the form of white solid (330 mg, yield: 58%). Elemental analysis calculated for C_20_H_20_NB (285.2): C 84.23, H 7.07, N 4.91; found C 84.05, H 7.24, N 5.29; NMR (CDCl_3_, 20 °C): ^1^H (600 MHz): δ 1.17 (s, 3H), 2.30 (s, 3H), 2.79 (s, 3H), 4.49 (br m, 1H, B-*H*), 6.63 (s, 1H), 7.01 (s, 1H), 7.27 (dd, ^3^ *J*_HH_ = 7.3, 5.7 Hz, 1H), 7.36 (dd, ^3^ *J*_HH_ = 7.8 Hz, 1H), 7.43 (dd, ^3^ *J*_HH_ = 7.8 Hz, 1H), 7.65 (d, ^3^ *J*_HH_ = 7.5 Hz, 1H), 7.94 (d, ^3^ *J*_HH_ = 7.7 Hz, 1H), 7.98 (dd, ^3^ *J*_HH_ = 7.4 Hz, 1H), 8.04 (d, ^3^ *J*_HH_ = 7.8 Hz, 1H), 8.35 (d, ^3^ *J*_HH_ = 5.7 Hz, 1H); ^13^C­{^1^H} (151 MHz): δ 21.1, 21.4, 25.4, 118.1, 121.3, 121.8, 125.3, 128.2, 128.4, 130.0, 130.7, 135.7, 136.2, 139.4, 141.4 (br, C–B), 142.9, 143.6, 144.6, 158.2, 163.4 (br, C–B); ^11^B (193 MHz): δ – 4.3 (d, *J*_BH_ ≈ 95 Hz); ^11^B­{^1^H} (193 MHz): δ – 4.3 (br s); IR (KBr) ν: 3055 (w), 3016 (w), 2968 (m), 2941 (m), 2912 (m), 2850 (w), 2333 (s, B–H), 1619 (m), 1600 (m), 1554 (m), 1477 (s), 1441 (s), 1417 (m), 1371 (w), 1322 (m), 1284 (w), 1267 (w), 1189 (w), 1160 (m), 1129 (w), 1082 (w), 1061 (m), 1016 (m), 995 (m), 946 (w), 884 (w), 851 (m), 778 (s), 744 (s), 686 (m), 663 (m), 590 (m), 528 (w), 433 (w) cm^–1^.

### Synthesis of **8**

To a solution of **7** (66.9 mg, 0.235 mmol) in DCM (5 mL) was added a solution of triphenylmethylium tetrakis­(pentafluorophenyl) borate (195 mg, 0.9 equiv) in 5 mL of DCM, and the mixture was stirred for 10 min at room temperature. Then, the solvent was evaporated, the residue dissolved in minimum amount of DCM (1 mL) and triturated with hexane (5 mL). This procedure was repeated twice, and the residue was then washed with hexane (3 × 5 mL) and dried *in vacuo*. Product **8** was obtained in the form of bright-yellow powder (185 mg, yield: 82%). Lower yield is most probably caused by losses in the trituration process employing DCM, in which the compound is highly soluble. Elemental analysis calculated for C_44_H_19_B_2_F_20_N (963.23): C 54.89, H 1.99, N 1.45; found C 53.62, H 2.15, N 1.49; NMR (CDCl_3_, 20 °C): ^1^H (600 MHz): δ 2.22 (s, 6H), 2.22 (s, 3H), 7.02 (s, 2H), 7.63 (t, ^3^ *J*_HH_ = 7.4 Hz, 1H), 7.68 (m, 1H), 7.78 (d, ^3^ *J*_HH_ = 7.5 Hz, 1H), 7.83 (d, ^3^ *J*_HH_ = 7.6 Hz, 1H), 7.93 (d, ^3^ *J*_HH_ = 7.2 Hz, 1H), 8.07 (d, ^3^ *J*_HH_ = 7.9 Hz, 1H), 8.30 (dd, ^3^ *J*_HH_ = 5.9, ^4^ *J*_HH_ = 1.4 Hz, 1H), 8.52 (td, ^3^ *J*_HH_ = 7.8, ^4^ *J*_HH_ = 1.6 Hz, 1H); ^11^B (193 MHz) δ 61.3 (br s), −17.7 (s, BAr^F^); ^11^B­{^1^H} (CDCl_3_, 193 MHz) δ 61.3 (br s), −17.7 (s, BAr^F^); ^19^F (565 MHz): δ −132.5 (s, 2F), −162.5 (s, 1F), −166.5 (s, 2F); ^13^C (151 MHz): 21.4, 23.1, 120.8, 123.8 (br, *i*-C_6_F_5_), 124.6, 126.5, 129.1, 133.6, 135.2, 136.2 (br d, ^1^ *J*_FC_ ≈ 248 Hz, C_6_F_5_), 138.1 (br d, ^1^ *J*_FC_ ≈ 245 Hz, C_6_F_5_), 139.1, 139.6, 140.9, 141.7, 143.9, 146.6, 148.1 (d, ^1^ *J*_FC_ = 240 Hz, C_6_F_5_), 153.8, 159.9; neither C–B signal was observed; HRMS (ESI): *m*/*z* [M]^+^ calculated for C_20_H_19_BN: 284.1605; found: 284.1632; HRMS (ESI): *m*/*z* [M]^−^ calculated for C_24_BF_20_: 678.9779; found: 678.9763.

### Catalytic Evaluation—Solvent and Catalyst Comparison Study

Samples were prepared using the following general procedure: 500 μL of substrate **9b** stock solution (*c* = 0.06 m, *c*_F_ = 0.01 m) in corresponding solvent was transferred into a 3 mL quartz cuvette followed by 2.4 mL of the solvent. Then, 300 μL of catalyst stock solution (*c* = 10^–4^ m, *c*_F_ = 10^–5^ m, *V*_F_ = 3 mL) prepared in the respective solvent was added. For each solvent tested, a corresponding blank was prepared using the same catalyst concentration (300 μL of a 10^–4^ m stock solution) in pure solvent (final volume 3 mL) to account for background absorbance. After mixing, the cuvettes were sealed and promptly transported to the UV–vis spectrometer. Spectra were recorded in the range of 400 to 700 nm every 5 min over a 90 min period at a constant temperature of 20 °C. Catalyst performance was assessed by monitoring the decrease in absorbance at 535 nm (the absorption maximum of the substrate) over time.

### Catalytic Evaluation—Substrate Study

The corresponding diazo compound (100 mg) was dissolved in CHCl_3_ (10 mL) and transferred to a dried Schlenk flask under an inert atmosphere. A solution of catalyst **8** (1 mol %) in chloroform (2 mL) was added, and the mixture was stirred overnight at room temperature at 60 °C. As the reaction progressed, gradual discoloration of the diazo compound was observed. After completion, the resulting light-yellow solution was concentrated under reduced pressure and the crude residue was purified by column chromatography on silica gel (eluent composition and retention factors are provided for each respective product). For characterization data of individual products, see the SI.

Theoretical calculations were performed using the Gaussian 16 program package.^45^ If available, the geometry optimizations were started from atomic coordinates determined by X-ray diffraction analysis, using the PBE0 density functional in conjunction with the def2-TZVP basis set with added Grimme’s D3 dispersion correction.^43^ Solvent effects (dichloromethane) have been approximated using the PCM model.^44^ The fluoride ion affinities (FIA) were calculated following the method developed by Greb et al.^34^ using the geometries and the thermal corrections obtained at the PBE0­(D3)/def2-TZVP level of theory combined with the PW6B95­(D3)/def2-QZVPPsingle-point electronic energies and anchored quasi-isodesmically with the Me_3_Si^+^/Me_3_SiF system.

## Supplementary Material





## Notes

Description of experimental procedures including complete characterization of prepared compounds, copies of the NMR spectra, crystallographic parameters, additional structure diagrams and further computational details (PDF)
Crystallographic data (XYZ)
