# Supplementary material for: Phenylpyridine-Based Borenium Salts as Lewis Acid Catalysts for Homocoupling of Diaryldiazomethanes
Source: Inorg Chem. 2026 Jan 5;65(2):1408–18. doi: 10.1021/acs.inorgchem.5c04965 (PMC12820930; doi:10.1021/acs.inorgchem.5c04965)
Supplement: Supplementary file 1 [file ic5c04965_si_001.pdf]

Supporting Information

Phenylpyridine-based borenium salts as Lewis acid catalysts for homocoupling of diaryldiazomethanes

Michaela Buziková,[a] Jiří Schulz,[b] Miroslava Litecká,[a] Karel Škoch[a,*]

[a] Institute of Inorganic Chemistry of Czech Academy of Sciences, Husinec-Řež 1001, 250 68, Czech Republic

[b] Department of Inorganic Chemistry, Faculty of Science, Charles University, Hlavova 2030, 128 40 Prague, Czech Republic

Corresponding author e-mail: skoch@iic.cas.cz

Table of contents

Experimental Part 2
Synthesis of Lewis acids 4
  Synthesis of **2** 4
  Synthesis of **3** 4
  Synthesis of **5S** 10
  Synthesis of **4NH** 15
  Synthesis of **6** 19
  Attempted hydride abstraction from compound **6** 22
  Synthesis of **7** 24
  Synthesis of **8** 28
  Determination of Buried Volume 32
  Preparation of hydrazones and diazomethanes 33
Catalytic evaluation 39
Solvent comparison study 39
  Catalyst screening 40
  Determination of rate constants in homocoupling of diazomethanes 42
  Characterization data of prepared tetraarylethylenes 43
Mechanistic investigation 45
Cross experiments 47
References – Supplementary Information 58

Experimental Part

Experiments were performed under dry argon atmosphere using standard Schlenk-type glassware and/or argon filled glovebox, unless stated otherwise. Reaction solvents hexane, toluene and tetrahydrofuran were dried by refluxing over sodium metal in the presence of benzophenone ketyl radical under argon atmosphere. Dichloromethane was purified by distillation from CaH_2 in argon atmosphere. Anhydrous solvents were stored over activated 4Å molecular sieves. Solvents used for extractions and/or column chromatography were used as received. Deuterated solvents were distilled from CaH_2 and stored over 4Å molecular sieves under argon atmosphere. All chemicals were purchased from commercially available resources (Sigma-Aldrich, BLD-Pharm, TCI, ABCR) and used as received, unless stated otherwise. Compounds **1, 2**, **3**, **12**, **13** and **14** were prepared according to reported procedure.[S1]

CAUTION: The diazo compounds investigated in this study are potentially unstable and may be prone to explosive decomposition, although no such incidents were observed during our experiments. Likewise, certain precursors (such as hydrazine or mercury oxide), may present additional toxicity hazards. All experimental work should therefore be conducted following appropriate safety protocols and established laboratory standards.

NMR spectra were recorded on JEOL Delta 600 spectrometer at 20 °C. ^{1}H and ^{13}C NMR chemical shifts (δ in ppm) are given relative to TMS and referenced to the residue solvent signal ($CDCl_3$: δ_H = 7.26, δ_C = 77.0, CD_2Cl_2: δ_H =5.32, δ_C = 53.8). ^{11}B, ^{19}F and ^{31}P are referenced according to the primary reference for the unified chemical shift scale following IUPAC recommendation [R.K. Harris. E.D. Becker, S.M. Cabral de Menezes, R. Goodfellow, P. Granger *Pure Appl. Chem.* **2001**, *73*, 1795-1818]. The multiplicity of the signals is indicated as s, d, t, q or m for singlets, doublets, triplets, quartets or multiplets, coupling constants *J* are given in Hertz as positive values regardless of their individual signs. Assignment of the peaks was supported by 2D experiments (COSY, HSQC and HMBC). MestreNova software package was used for analysing the spectra.

Elemental Analysis was performed on FlashSmart™ Elemental Analyzer. Tin capsules were filled with samples and sealed in glovebox atmosphere. Mass spectrometry measurements were performed on Orbitrap Exploris™ 120 instrument using electrospray ionization. Infrared spectra were collected on Nexus 670 FT-IR spectrometer. Air stable samples were analysed by ATR method, samples sensitive to ambient conditions were measured as KBr pellets prepared in glovebox atmosphere. Samples were measured in range 4000-400 cm^{-1} and the relative intensities are given in parentheses (s, m, w for strong, medium and weak). UV-Vis kinetic experiments were recorded on Perkin Elmer Lambda 35 Spectrometer, with the sample temperature maintained at a constant value of 20 °C using a PTP-1 Peltier Temperature Programmer (PerkinElmer Instruments).

X-ray diffraction analysis of the compounds **5O**, **5S**, **4NH**, **7** and **8** was performed with a Rigaku XtaLAB Synergy S diffractometer equipped using Cu (Cu/Kα radiation; λ = 1.54184 Å) with micro-focus X-ray source and Hybrid Pixel Array Detector (HyPix-6000HE). Oxford Cryosystems (Cryostream 800) cooling device was used for data collection at a temperature of 100 K. CrysAlisPro software[S2] was used for data collection and cell refinement, data reduction and absorption correction. Data were corrected for absorption effects using empirical absorption correction (spherical harmonics), implemented in SCALE3 ABSPACK scaling algorithm and numerical absorption correction based on gaussian integration over a multifaceted crystal model was applied as described by Coppens *et al.*[S3] The structures of prepared compounds were solved with the SHELXT[S4] structure solution program using Intrinsic Phasing and refined with the refinement package using SHELXL[S5] Least Squares minimization implemented in Olex2[S6]. Anisotropic displacement parameters were refined for all non-H atoms. The

hydrogen atoms were calculated to idealized positions, or found in the Fourier map (Boronic hydrogens). For crystallographic data and structure refinement see below. Molecular graphic for all crystal structures was generated using DIAMOND software.[S7]

Theoretical calculations were performed using the Gaussian 16 program package.[S8] If available, the geometry optimizations were started from atomic coordinates determined by X-ray diffraction analysis, using PBE0[S9] density functional in conjunction with the def2-TZVP[S10] basis set with added Grimme's D3 dispersion correction.[S11] Solvent effects (dichloromethane) have been approximated using PCM model.[S12] The fluoride ion affinities (FIA) were calculated following the method developed by Greb et al.[S13] using the geometries and the thermal corrections obtained at the PBE0(D3)/def2-TZVP level of theory combined with the PW6B95(D3)/def2-QZVPP single-point electronic energies and anchored quasi-isodesmically with the Me_3Si^+/Me_3SiF system.

Synthesis of Lewis acids

Synthesis of 2

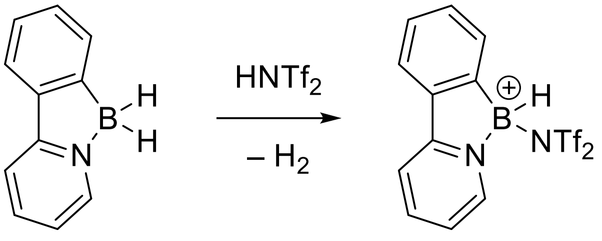

1 **2**

To a stirred solution of **1** (148.4 mg, 0.89 mmol) in 5 mL of anhydrous DCM was added solution of bistriflimide (249.8 mg, 1 equiv.) in DCM (5 mL), upon which the release of hydrogen gas was observed. The mixture was left stirring at room temperature for 30 minutes. Then the solvent was evaporated and resulting product was washed with hexane (3x 3 mL) and dried, which provided product **2** in the form of white powder (375 mg, 95 %).

Characterization data are in accordance with those previously published.[S1]

Synthesis of 3

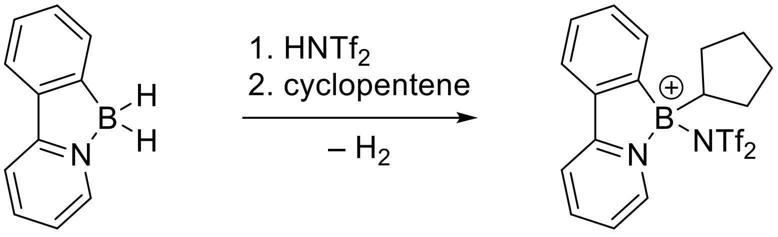

1 **3**

To a stirred solution of freshly prepared **2** (286.4 mg, 0.64 mmol) in 5 mL of anhydrous DCM was added cyclopentene (65.6 mg, 1.5 equiv.) in DCM (3 mL). The mixture was left stirring at room temperature for 1 hour, upon which the solvent and excess cyclopentene were evaporated *in vacuo*. The residue was washed with hexane and evaporated to dryness, providing **3** in the form of white solid (322 mg, 98 %).

Characterization data are in accordance with those previously published.[S1]

Synthesis of 4O

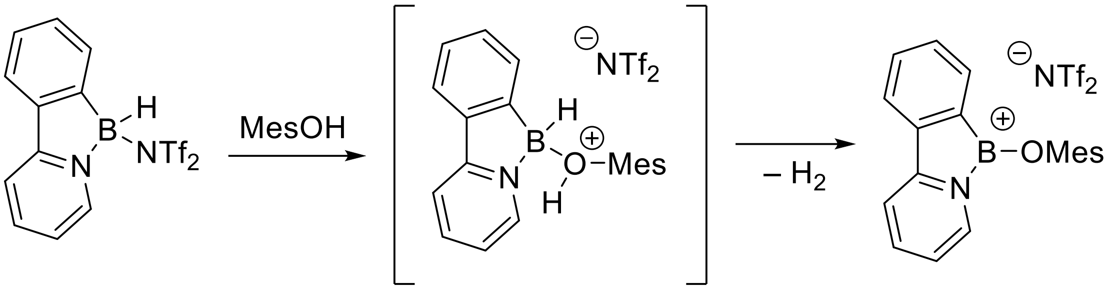

2 **4O** **5O**

NMR monitoring

To a freshly prepared compound **2** (88.6 mg, 0.15 mmol) in 0.4 mL CD_2Cl_2 in Young NMR tube was added mesityl alcohol (20.7 mg, 1.0 equiv.) in 0.2 mL CD_2Cl_2. The reaction was monitored by ^{1}H and ^{11}B NMR, confirming a formation of the intermediate **4O**. The spectra were acquired over several days, during which the initially formed compound **4O** gradually converted into the final product **5O** (see spectra below).

*NMR characterization of in situ generated **4O**; peak assignment is tentative*

4O NMR (CD_2Cl_2, 20 °C): ^{1}H (600 MHz): δ 1.95 (s, 12H), 2.18 (s, 6H), 4.29 (br m, B-*H*), 6.73 (s, 4H), 7.63 (dt, $^3J_{HH}$ = 7.7 Hz, $^4J_{HH}$ = 1.0 Hz, 1H), 7.96 (dt, $^3J_{HH}$ = 7.9 Hz, $^4J_{HH}$ = 1.5 Hz, 1H), 8.49 (td, $^3J_{HH}$ = 7.9 Hz, $^4J_{HH}$ = 1.6 Hz, 1H), 8.55 (td, $^3J_{HH}$ = 6.5 Hz, $^4J_{HH}$ = 1.6 Hz, 1H), 13.22 (br s, O-*H*);

^{11}B (193 MHz) δ 25.4 (br s); ^{11}B{^{1}H} (193 MHz) δ 25.4 (s);

^{19}F (565 MHz) δ –78.99 (s).

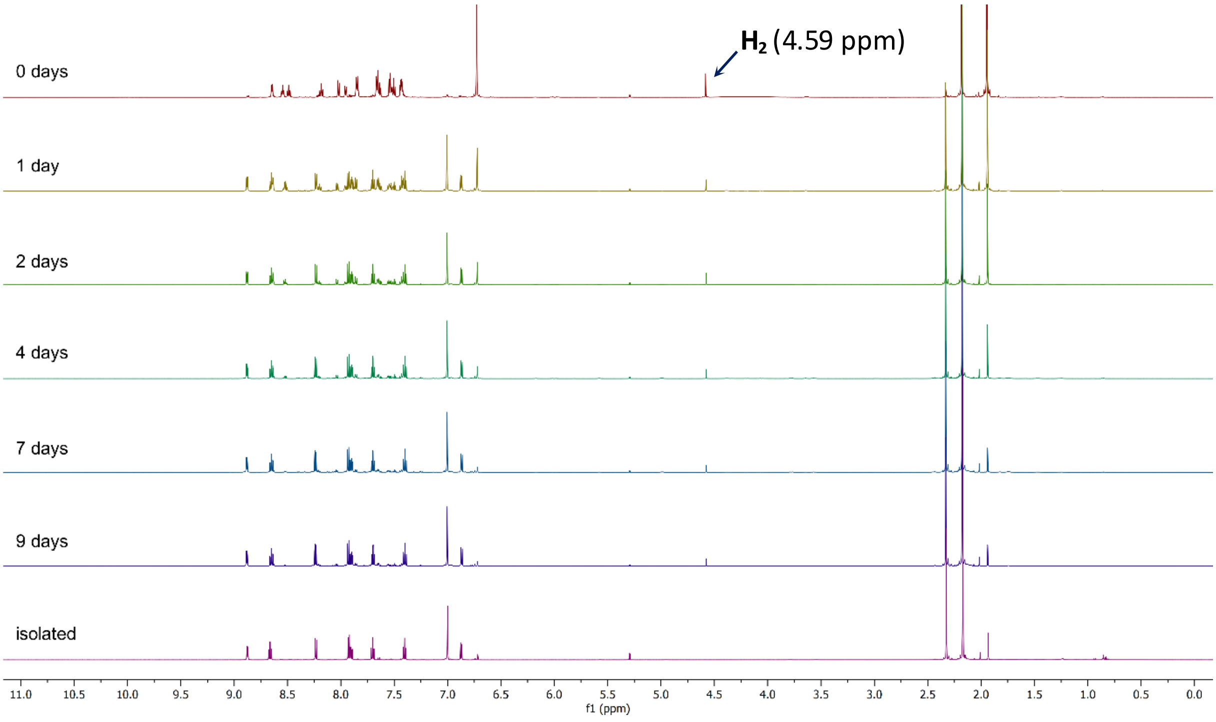

Figure S1: ^{1}H NMR (CD_2Cl_2, 600 MHz, 20°C) monitoring of reaction kinetics in formation of **5O**.

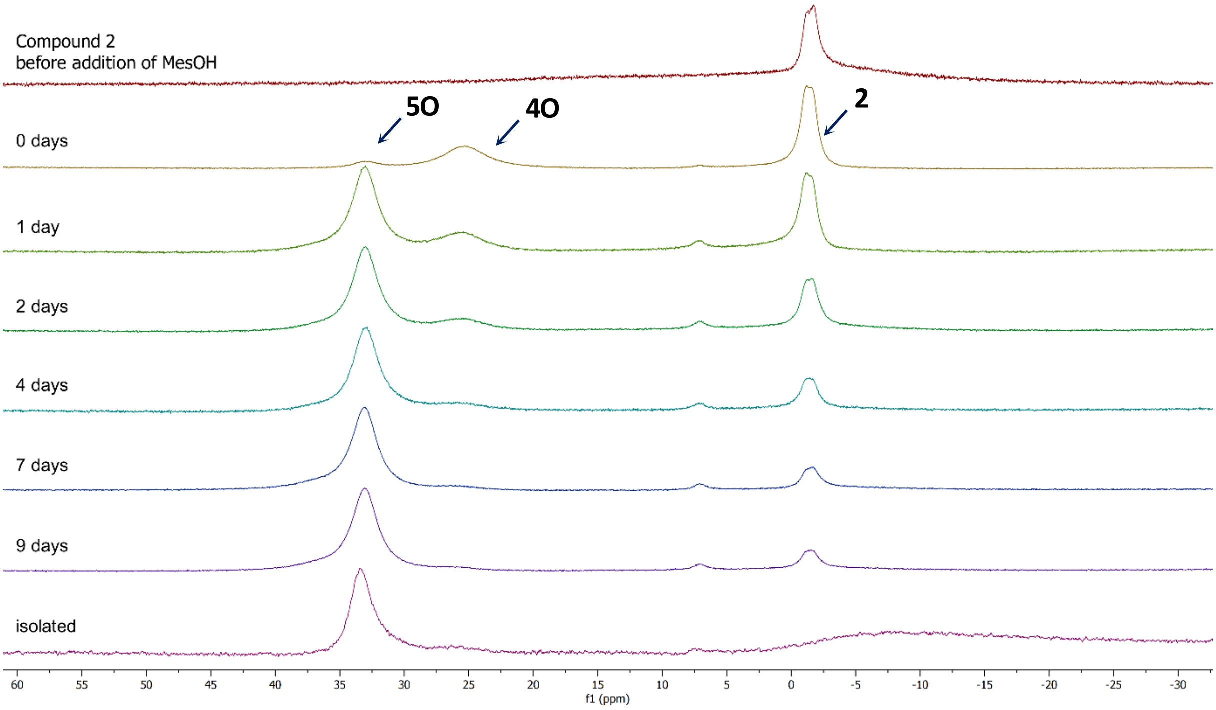

Figure S2: $^{11}B\{^1H\}$ NMR (CD_2Cl_2, 193 MHz, 20 °C) monitoring of reaction kinetics in formation of **5O**.

Preparative experiment:

To a stirring solution of **2** (292 mg, 0.65 mmol) in 5 mL DCM was added mesityl alcohol (89.1 mg, 0.65 mmol, 1 equiv.) in DCM (5 mL). The mixture was stirred at room temperature for 10 days to ensure the reaction completion. Then the solvent was evaporated under reduced pressure, the residue redissolved in minimum amount of DCM (ca 1 mL), layered with hexane (ca 8 mL) and left overnight for crystallization. Obtained crystals were filtered, washed with hexane and dried, providing **5O** in the form of yellow crystalline solid (345 mg, yield: 91 %).

Characterization of isolated **5O**

Elemental analysis calculated for $C_{22}H_{19}BF_6N_2O_5S_2$ (580.32): C 45.53, H 3.30, N 4.83; found C 44.69, H 3.42, N 4.38;

NMR ($CDCl_3$, 20 °C): ^1H (600 MHz): δ 2.17 (s, 6H, *o*-CH_3), 2.34 (s, 3H, *p*-CH_3), 6.85 (d, $^3J_{HH}$ = 7.2 Hz), 6.96 (s, 2H, C*H* mesityl), 7.37 (t, $^3J_{HH}$ = 7.5 Hz, 1H), 7.66 (td, $^3J_{HH}$ = 7.7, $^4J_{HH}$ = 1.2 Hz, 1H), 7.89 (ddd, $^3J_{HH}$ = 7.3, 5.7 Hz; $^4J_{HH}$ = 0.97 Hz, 1H), 7.94 (d, $^3J_{HH}$ = 7.7 Hz, 1H), 8.27 (d, $^3J_{HH}$ = 8.0 Hz, 1H), 8.63 (td, $^3J_{HH}$ = 7.9 Hz; $^4J_{HH}$ = 1.5 Hz, 1H), 8.87 (m, 1H);

^{11}B (193 MHz) δ 31.5; $^{11}B\{^1H\}$ (193 MHz) δ 31.7 (s);

^{19}F (565 MHz) δ –78.6 (s);

$^{13}C\{^1H\}$ (151 MHz) δ 16.6, 20.9, 119.8 (q, $^1J_{FC}$ = 321 Hz), 121.3, 124.7, 126.6, 127.3, 130.1, 134.0, 134.5, 135.5, 136.3, 141.7, 143.1, 148.3, 152.5, 158.2; signal of C-B was not observed;

HRMS (ESI): *m/z* $[M]^+$ calculated for $C_{20}H_{19}NBO$: 300.1554; found: 300.1551;

HRMS (ESI): *m/z* $[M]^-$ calculated for $C_2F_6NO_4S_2$: 279.9178; found: 279.9169;

IR (KBr): 3120 (m), 3095 (m), 3069 (m), 2958 (m), 2925 (m), 2866 (w), 1631 (s), 1609 (m), 1568 (w), 1528 (m), 1508 (s), 1486 (m), 1437 (s), 1352 (s), 1275 (m), **1198 (s, NTf_2)**, 1136 (s), 1096 (m), 1056

(s), 957 (w), 900 (m), 852 (m), 791 (m), 779 (m), 763 (m), 741 (s), 730 (s), 653 (m), 619 (s), 575 (s), 514 (s), 430 (w) cm^{-1}.

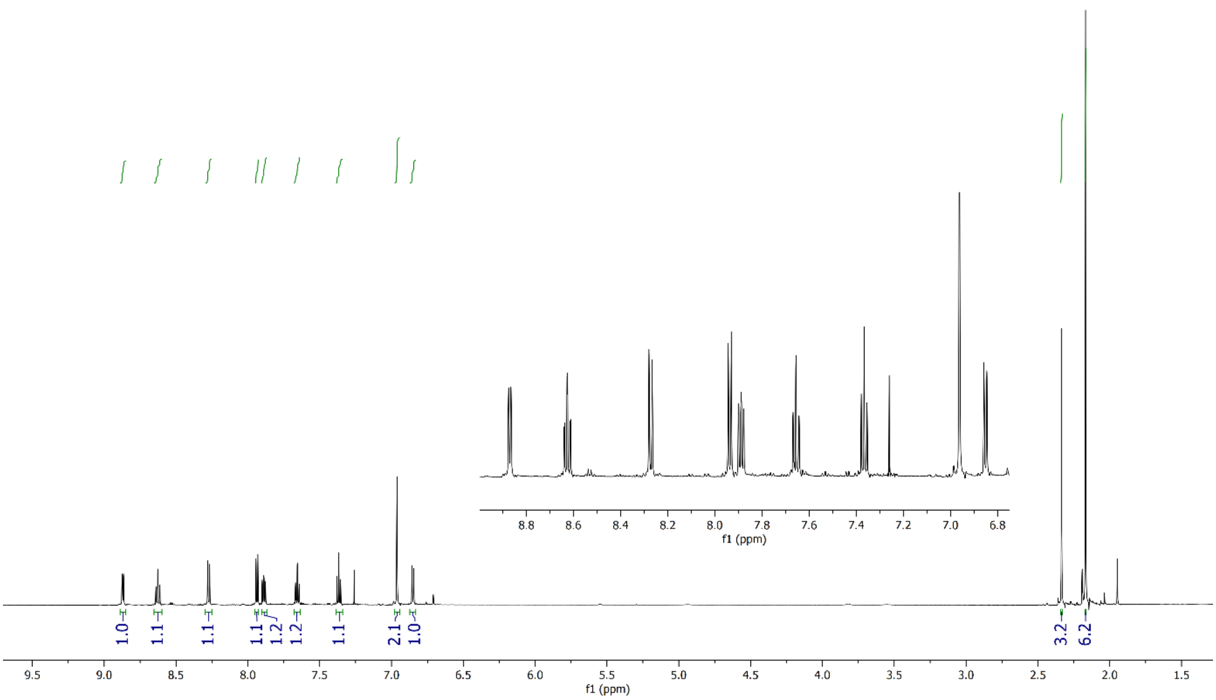

Figure S3: ^{1}H NMR (600 MHz, $CDCl_3$, 20 °C) spectrum of compound **5O**.

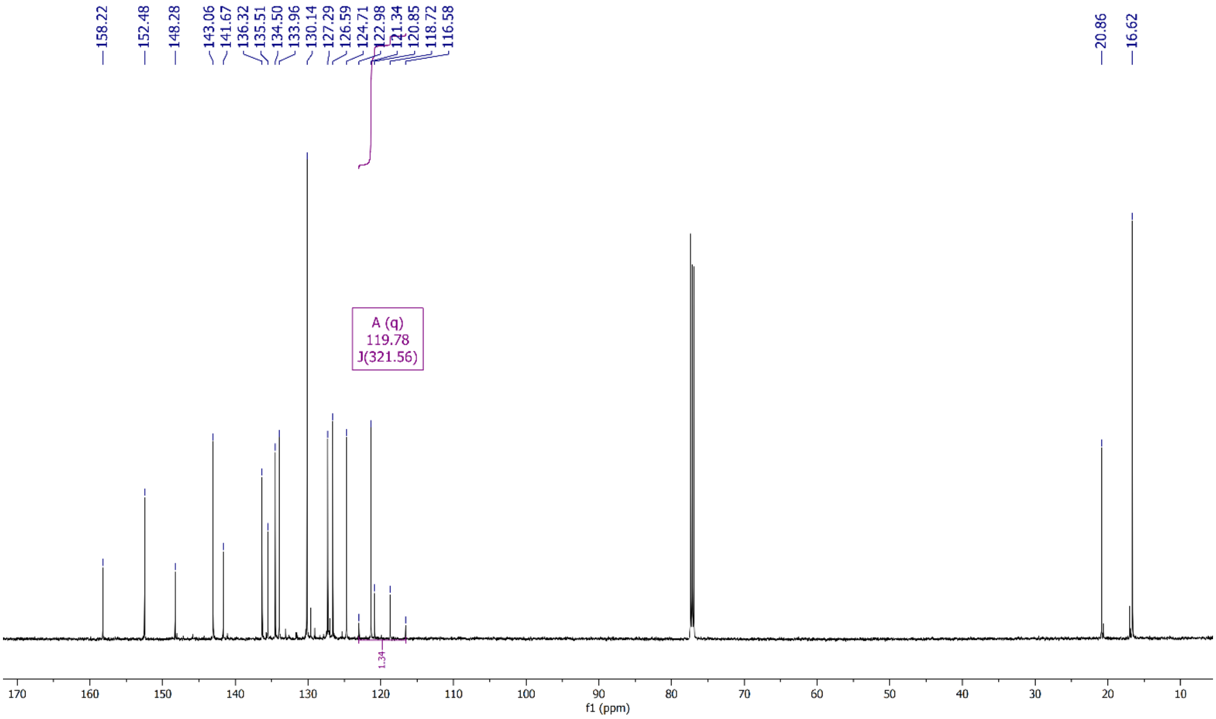

Figure S4: ^{13}C{^{1}H} NMR (151 MHz, $CDCl_3$, 20 °C) spectrum of compound **5O**.

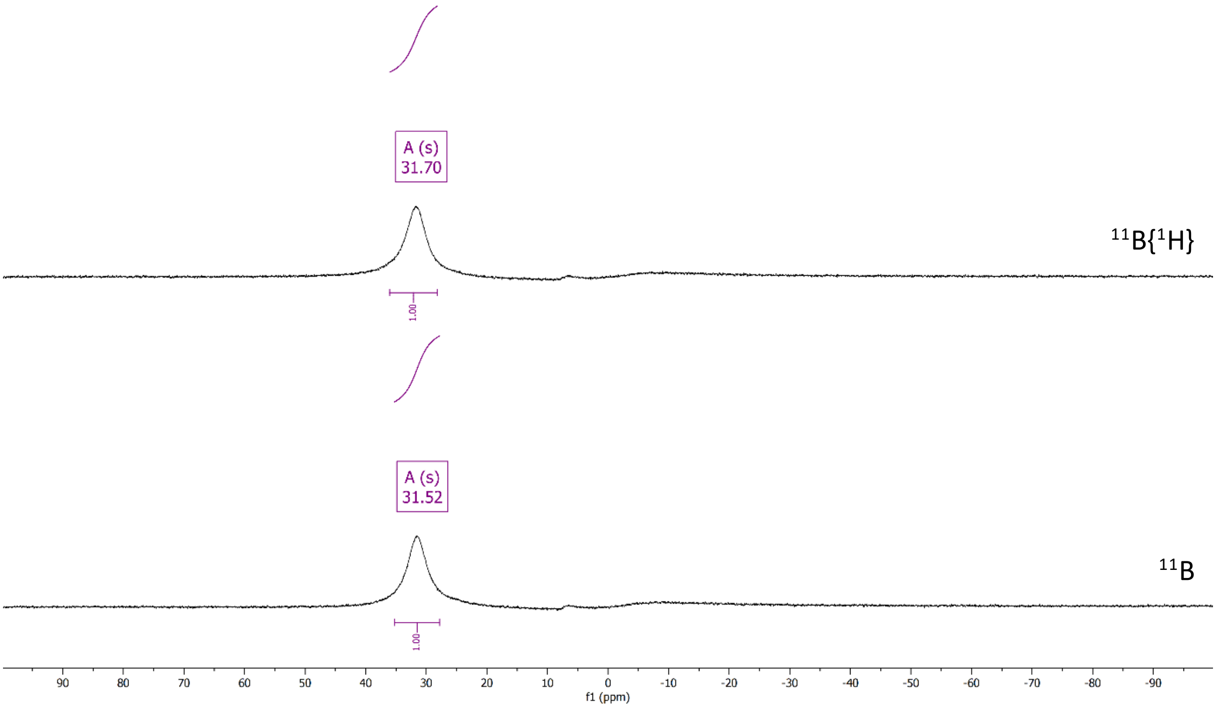

Figure S5: ^{11}B and $^{11}B\{^1H\}$ NMR (193 MHz, $CDCl_3$, 20 °C) spectra of compound **5O**.

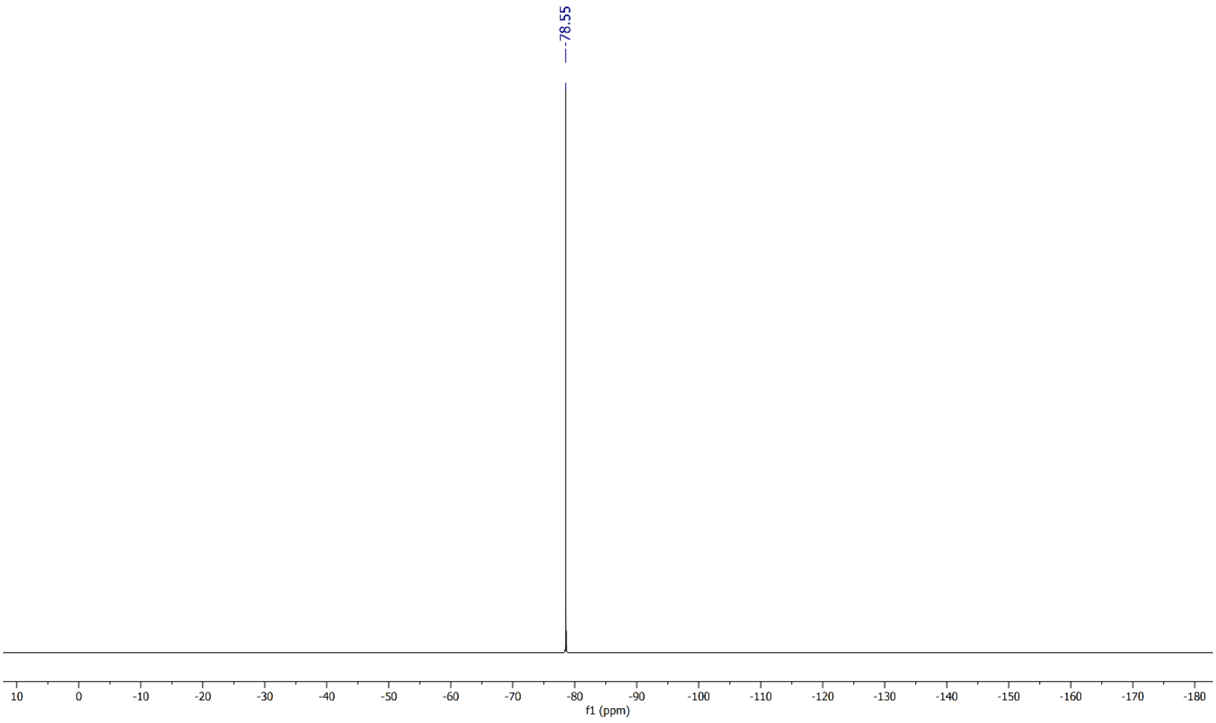

Figure S6: ^{19}F NMR (564 MHz, $CDCl_3$, 20 °C) spectrum of compound **5O**.

Crystal structure determination of 5O [KSK390_auto]

Suitable crystal for SC-XRD structure determination was obtained by diffusion of hexane into dichloromethane solution of **5O** at room temperature.

Crystal Data for $C_{22}H_{19}BF_6N_2O_5S_2$ (M =580.32 g/mol): monoclinic, space group $P2_1/c$ (no. 14), a = 11.0163(2) Å, b = 16.8551(3) Å, c = 13.4372(3) Å, β = 95.911(2)°, V = 2481.76(8) Å^3, Z = 4, T = 100.00(10) K, μ(Cu $K\alpha$) = 2.712 mm^{-1}, D_{calc} = 1.553 g/cm^3, 16576 reflections measured (8.068° ≤ 2Θ ≤ 133.19°), 4369 unique (R_{int} = 0.0332, R_{sigma} = 0.0305) which were used in all calculations. The final R_1 was 0.0323 ($I > 2\sigma(I)$) and wR_2 was 0.0850 (all data). CCDC Deposition Number: **2488312**.

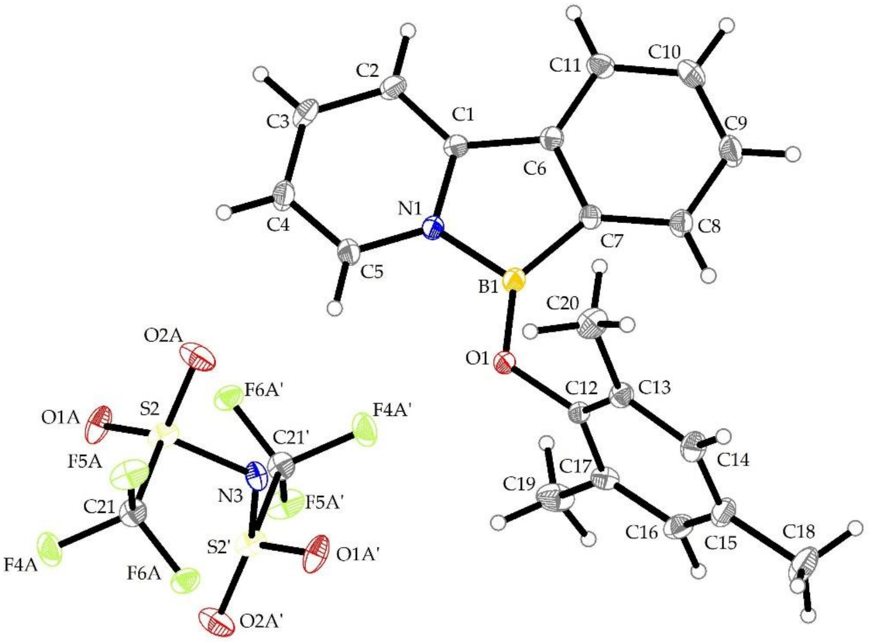

Figure S7: Crystal structure of **5O** (thermal ellipsoids shown at 30 % probability level).

Synthesis of 5S

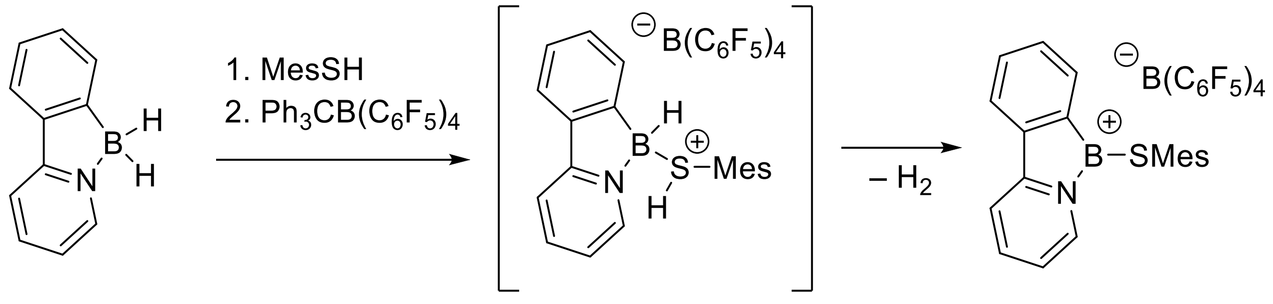

To a Young NMR tube with the mixture of **1** (22.1 mg, 0.13 mmol) and mesityl thiol (20.2 mg, 0.13 mmol, 1 equiv.) in CD_2Cl_2 (0.4 mL) was added triphenylmethylium tetrakis(pentafluorophenyl) borate (122.1 mg, 0.13 mmol, 1 equiv.) in CD_2Cl_2 (0.2 mL). Reaction progress was monitored by ^{1}H and ^{11}B NMR for 12 hours at 20 °C. After this time, the spectra of the reaction mixture were no longer changing. Product was obtained by diffusion of hexane directly into the CD_2Cl_2 solution, which crystallized the product and efficiently removed the triphenylmethane byproduct. Product **5S** was obtained after crystallization and drying in the form of yellow-brown crystals (117 mg, yield: 89 %).

The sample was obtained with ca. 90% purity as judged by NMR; additional peaks (ca. 10 %) correspond to residual trityl-derived material.

Characterization of **5S**

NMR (CD_2Cl_2, 20 °C): ^{1}H (600 MHz): δ 2.38 (s, 6H, *o*-C*H*$_3$), 2.41 (s, 3H, *p*-C*H*$_3$), 7.18 (s, 2H, C*H* mesityl), 7.34 (t, $^3J_{HH}$ = 7.6 Hz, 1H), 7.64 (m, 1H), 7.66 (m, 1H), 7.77 (d, $^3J_{HH}$ = 7.6 Hz, 1H), 7.80 (m, 1H), 7.87 (m, 1H), 8.06 (d, $^3J_{HH}$ = 8.1 Hz, 1H), 8.54 (td, $^3J_{HH}$ = 7.9 Hz, $^4J_{HH}$ = 1.5 Hz, 1H);

^{11}B (193 MHz) δ –17.7 (s, BAr^F), 53.5 (br s); ^{11}B{^{1}H} (193 MHz) δ –17.7 (s, BAr^F), 53.5 (br s);

^{19}F (565 MHz) δ –167.3 (s, 2F), –163.4 (s, 1F), –133.0 (s, 2F);

^{13}C{^{1}H} (151 MHz) δ 21.3, 22.7, 119.4, 120.9, 124.2, 124.6 (br, *i*-C_6F_5), 126.5, 130.9, 131.0, 135.4, 136.7 (br d, $^3J_{FC} \approx$ 245 Hz, C_6F_5), 138.6 (br d, $^3J_{FC} \approx$ 245 Hz, *p*-C_6F_5), 140.3, 141.5, 143.1, 143.4, 144.0, 148.5 (br d, $^1J_{FC} \approx$ 241 Hz C_6F_5), 152.6, 159.0;

IR (KBr): *ν*: 3143 (w), 3088 (m), 3061 (w), 2958 (m), 2923 (m), 2872 (w), 1644 (s), 1363 (s), 1584 (m), 1568 (w), 1514 (s), 1460 (s), 1375 (m), 1357 (m), 1315 (w), 1299 (w), 1275 (s), 1238 (m), 1219 (s), 1178 (m), 1167 (m), 1153 (m), 1083 (s), 1035 (w), 1028 (w), 986 (s), 907 (w), 853 (m), 9807 (w), 788 (w), 775 (s), 756 (s), 725 (s), 709 (m), 683 (s), 661 (s), 610 (m), 602 (m), 576 (s) 549 (w), 448 (w), 474 (w), 430 (w) cm^{-1}.

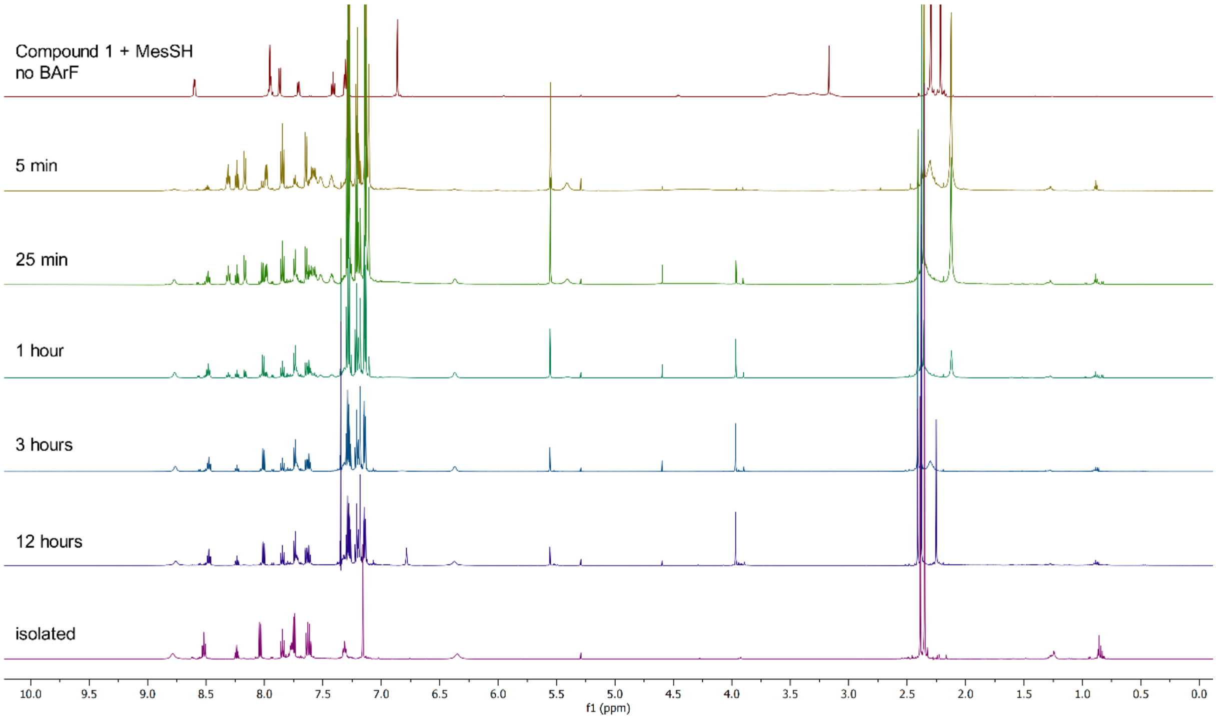

Figure S8: ^{1}H NMR (CH_2Cl_2, 600 MHz, 20 °C) monitoring of reaction kinetics of **5S** formation.

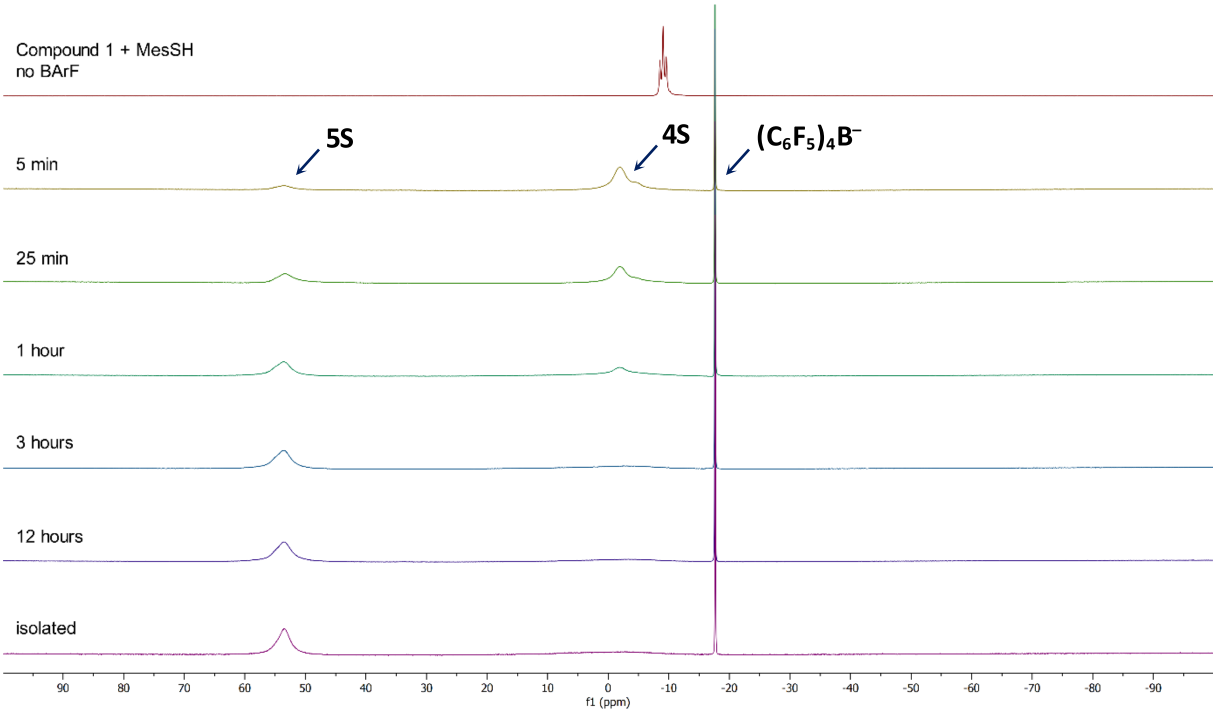

Figure S9: $^{11}B\{^{1}H\}$ NMR (CH_2Cl_2, 193 MHz, 20 °C) monitoring of reaction kinetics of **5S** formation.

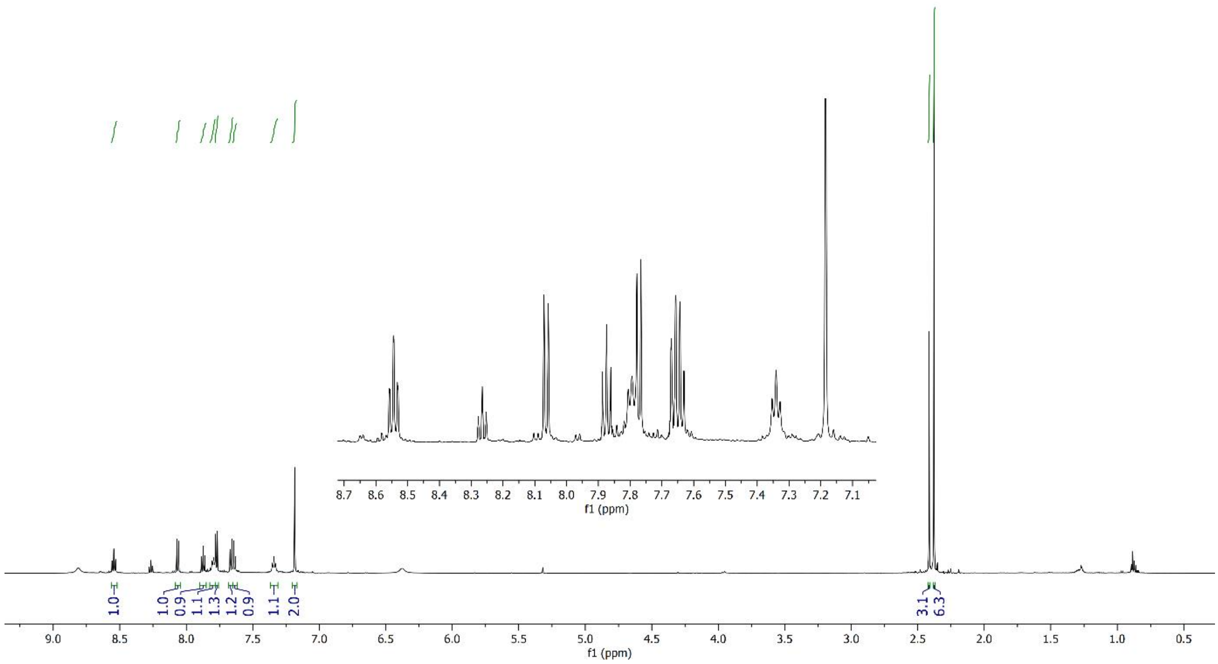

Figure S10: ^{1}H NMR (600 MHz, CD_2Cl_2, 20 °C) spectrum of compound **5S**.

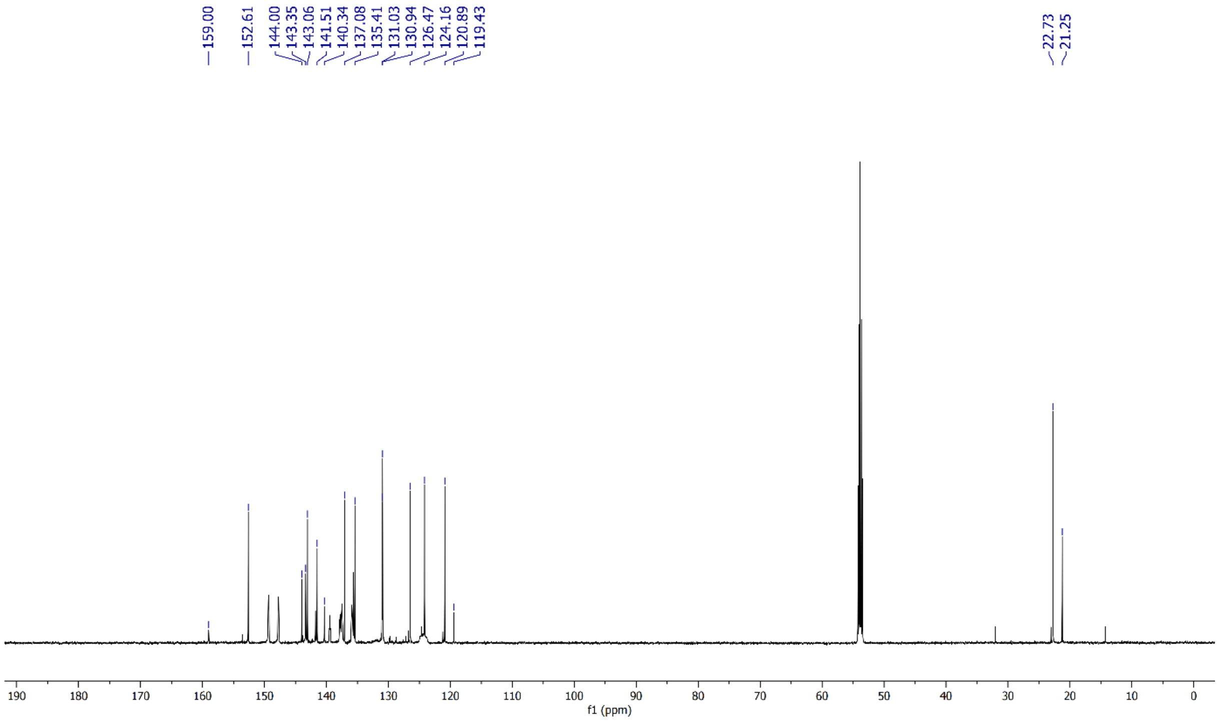

Figure S11: $^{13}C\{^{1}H\}$ NMR (151 MHz, CD_2Cl_2, 20 °C) spectrum of compound **5S**.

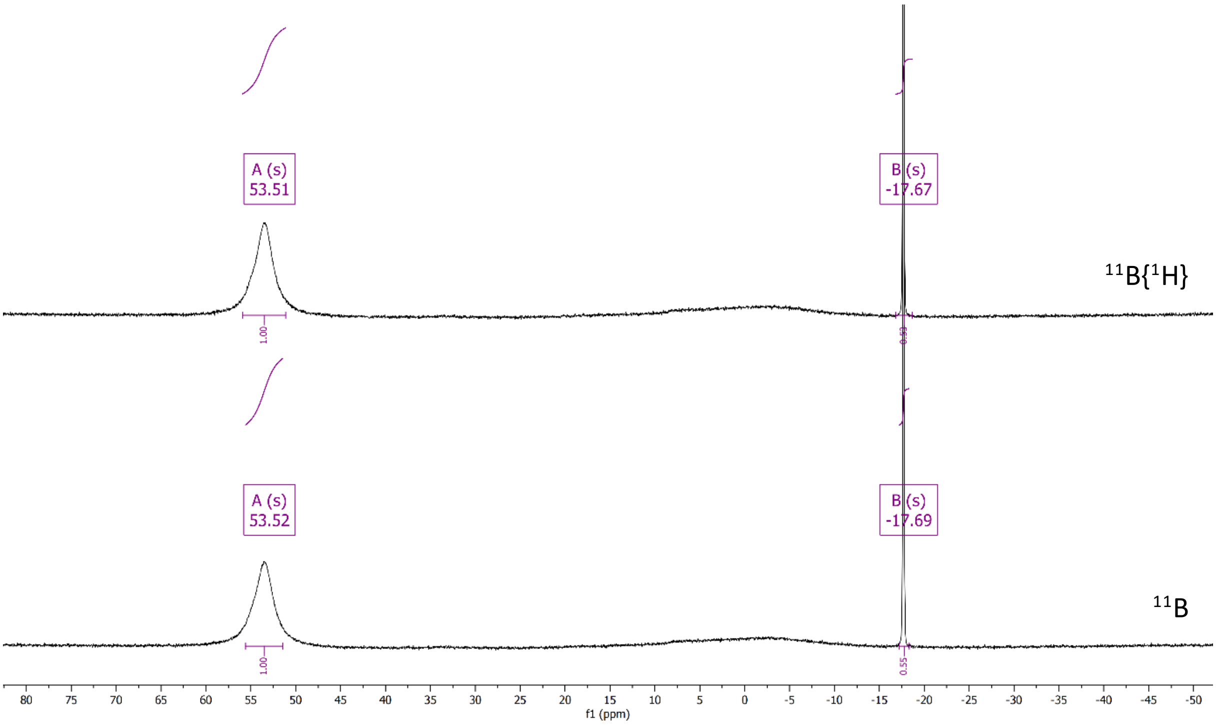

Figure S12: ^{11}B and ^{11}B{^{1}H} NMR (193 MHz, CD_2Cl_2, 20 °C) spectra of compound **5S**.

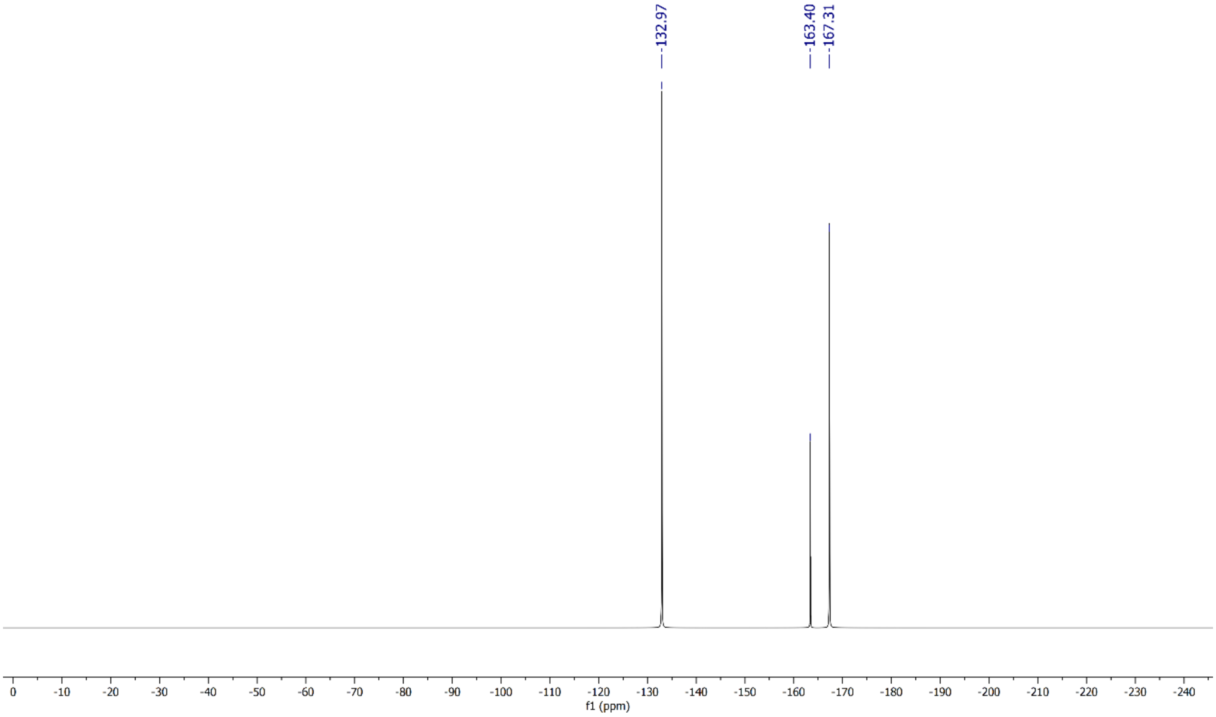

Figure S13: ^{19}F NMR (564 MHz, CD_2Cl_2, 20 °C) spectrum of compound **5S**.

Crystal structure determination of 5S [MB125-PP2-3-gg]

Suitable crystal for SC-XRD structure determination was obtained by diffusion of hexane into dichloromethane solution of **5S** at room temperature.

Crystal Data for $C_{44}H_{19}B_2F_{20}NS$ (M =995.28 g/mol): monoclinic, space group $P2_1/c$ (no. 14), a = 20.1483(8) Å, b = 10.9477(4) Å, c = 18.1416(7) Å, β = 97.515(4)°, V = 3967.3(3) Å^3, Z = 4, T = 100.00(11) K, μ(Cu $K\alpha$) = 1.931 mm^{-1}, D_{calc} = 1.666 g/cm^3, 29331 reflections measured (8.854° ≤ 2Θ ≤ 133.182°), 6979 unique (R_{int} = 0.0765, R_{sigma} = 0.0619) which were used in all calculations. The final R_1 was 0.0588 ($I > 2\sigma(I)$) and wR_2 was 0.1717 (all data). CCDC Deposition Number: **2488314**.

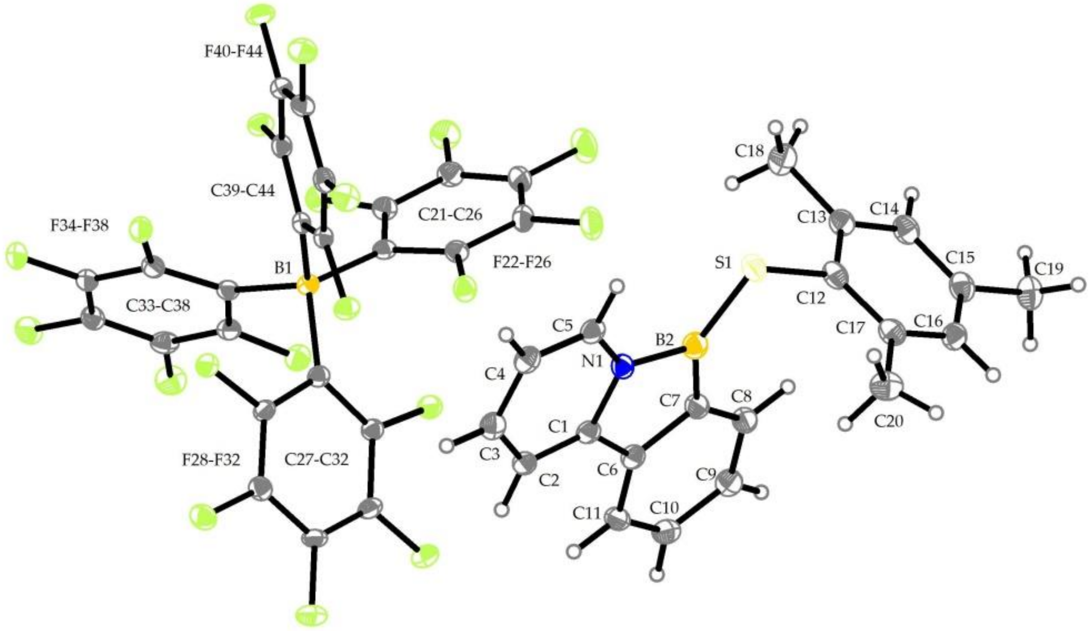

Figure S14: Crystal structure of **5S** (thermal ellipsoids shown at 30 % probability level).

Synthesis of 4NH

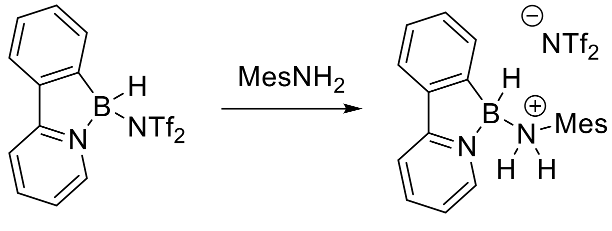

To a stirring solution of **2** (394 mg, 0.88 mmol) in DCM (10 mL) was added mesityl amine (119.4 mg, 1 equiv.) in DCM (5 mL) and the solution was left stirring at room temperature for 1 hour. Then the solvent was evaporated and the residue triturated with hexane (3x 3 mL). Upon solvent evaporation and drying *in vacuo*, **4NH** was obtained in the form of white powder (490 mg, yield: 95 %).

Characterization of **4NH**

Elemental analysis calculated for $C_{22}H_{22}BF_6N_3O_4S_2$ (581.36): C 45.45, H 3.81, N 7.23; found C 44.91, H 3.27, N 7.07;

NMR ($CDCl_3$, 20 °C): ^{1}H (600 MHz): δ 1.60 (br s, 3H), 2.33 (s, 3H), 2.54 (br s, 3H), 3.95 (br s, 1H, B-*H*), 6.24 (br m, 1H, N*H*), 6.66 (d, $^3J_{HH}$ = 7.3 Hz, 1H), 6.91 (br s, 2H), 7.28 (t, $^3J_{HH}$ = 7.3 Hz, 1H), 7.39 (br d, $^2J_{HH}$ = 13.4 Hz, 1H, N*H*), 7.44 (td, $^3J_{HH}$ = 7.6 Hz, $^4J_{HH}$ = 1.1 Hz, 1H), 7.65 (ddd, $^3J_{HH}$ = 7.2, 5.6 Hz; $^4J_{HH}$ = 1.2 Hz, 1H), 7.85 (d, $^3J_{HH}$ = 7.7 Hz, 1H), 8.07 (d, $^3J_{HH}$ = 8.1 Hz, 1H), 8.27 (td, $^3J_{HH}$ = 7.8, $^4J_{HH}$ = 1.4 Hz, 1H), 8.85 (d, $^3J_{HH}$ = 5.8 Hz, 1H);

$^{13}C\{^1H\}$ (151 MHz): δ 17.5, 20.8, 118.9, 119.4 (q, $^3J_{FC}$ = 321 Hz), 122.2, 123.9, 129.1, 130.3, 130.4, 131.7, 132.1, 137.3, 137.9, 144.5, 145.4, 146.8 (*C*-B), 158.4;

^{11}B (193 MHz): δ –2.8, $^{11}B\{^1H\}$ (193 MHz) δ –2.7 (s);

^{19}F (565 MHz): δ –78.9 (s);

HRMS (ESI): *m/z* $[M]^+$ calculated for $C_{20}H_{22}BN_2$: 301.1871; found: 301.1902;

HRMS (ESI): *m/z* $[M]^-$ calculated for $C_2F_6NO_4S_2$: 279.9178; found: 279.9167;

IR (KBr): 3226 (m, NH), 3145 (m, NH), 3070 (w), 2926 (w), 2866 (w), **2466 (m, BH)**, 1628(s), 1595 (m), 1541 (w), 1491 (s), 1452 (m), 1342 (s), 1231 (s), 1196 (br s, NTf_2), 1134 (s), 1108 (s), 1080 (m), 1059 (s), 1010 (w), 939 (w), 859 (m), 791 (w), 763 (m), 740 (m), 663 (m), 654 (m), 615 (m), 601 (s), 572 (s), 509 (m), 461 (w) cm^{-1}.

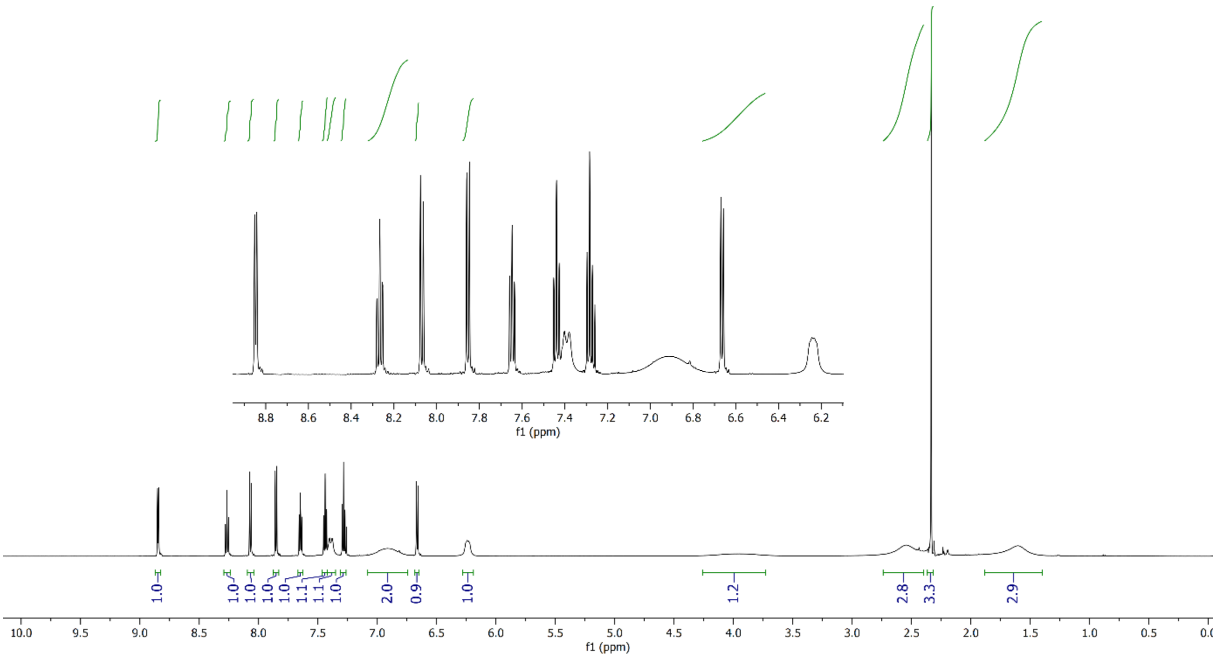

Figure S15: ^{1}H NMR (600 MHz, $CDCl_3$, 20 °C) spectrum of compound **4NH**.

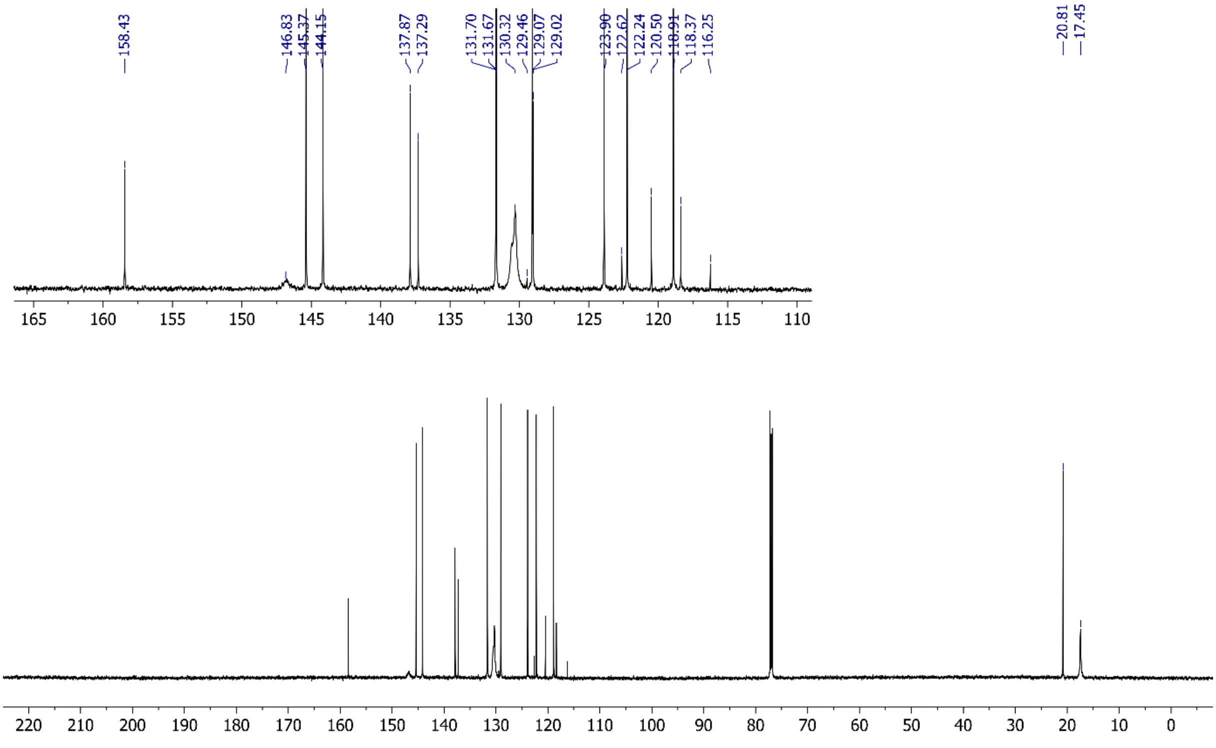

Figure S16: $^{13}C\{^{1}H\}$ NMR (151 MHz, $CDCl_3$, 20 °C) spectrum of compound **4NH**.

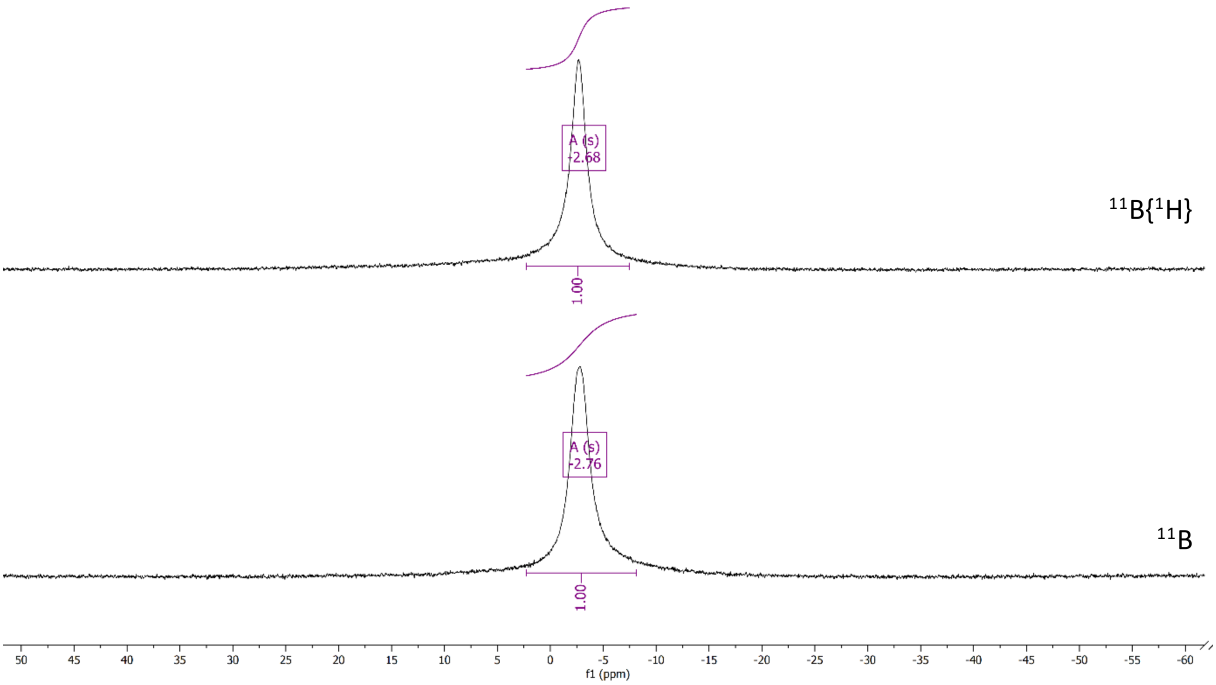

Figure S17: ^{11}B and $^{11}B\{^{1}H\}$ NMR (193 MHz, $CDCl_3$, 20 °C) spectra of compound **4NH**.

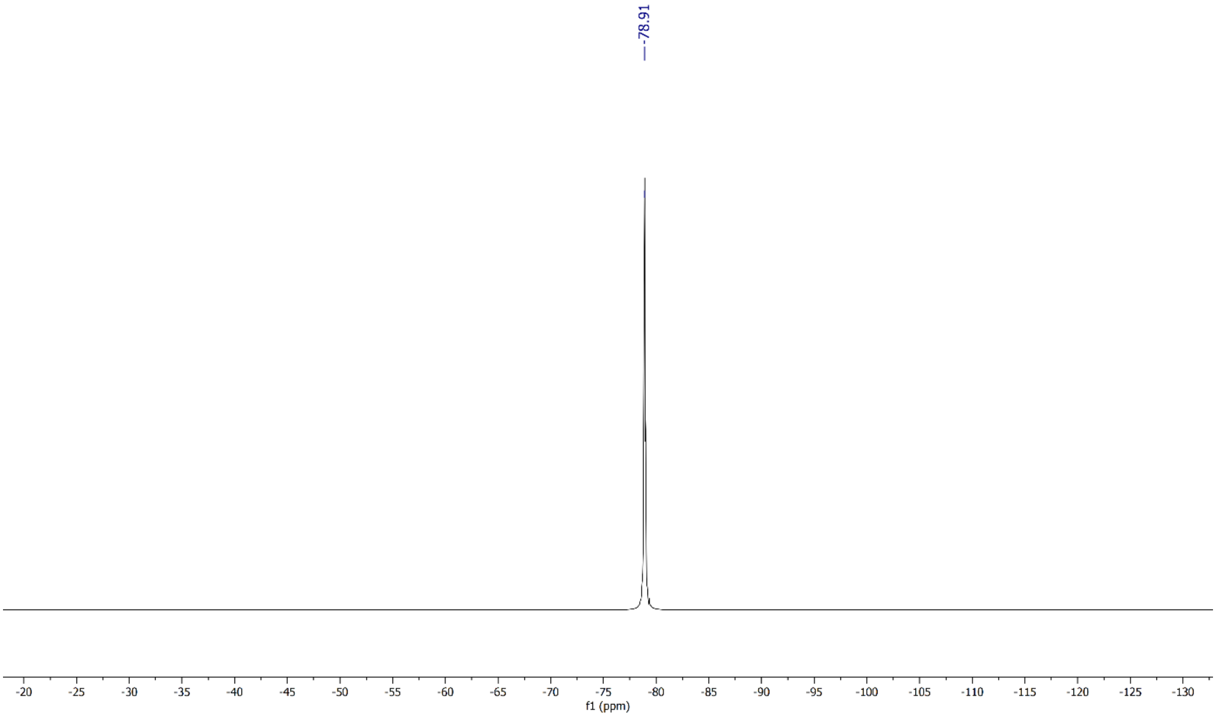

Figure S18: ^{19}F NMR (564 MHz, $CDCl_3$, 20 °C) spectrum of compound **4NH**.

Crystal structure determination of 4H [MB068-1-gg]

Suitable crystal for SC-XRD structure determination was obtained by diffusion of hexane into dichloromethane solution of **4NH** at room temperature.

Crystal Data for $C_{44}H_{44}B_2F_{12}N_6O_8S_4$ (M =1162.71 g/mol): orthorhombic, space group $Pna2_1$ (no. 33), a = 16.3505(2) Å, b = 18.4648(3) Å, c = 16.7651(2) Å, V = 5061.51(11) Å^3, Z = 4, T = 99.99(10) K, μ(Cu $K\alpha$) = 2.639 mm^{-1}, D_{calc} = 1.526 g/cm^3, 31866 reflections measured (7.122° ≤ 2Θ ≤ 133.192°), 8279 unique (R_{int} = 0.0466, R_{sigma} = 0.0410) which were used in all calculations. The final R_1 was 0.0449 ($I > 2\sigma(I)$) and wR_2 was 0.1178 (all data). CCDC Deposition Number: **2488313**.

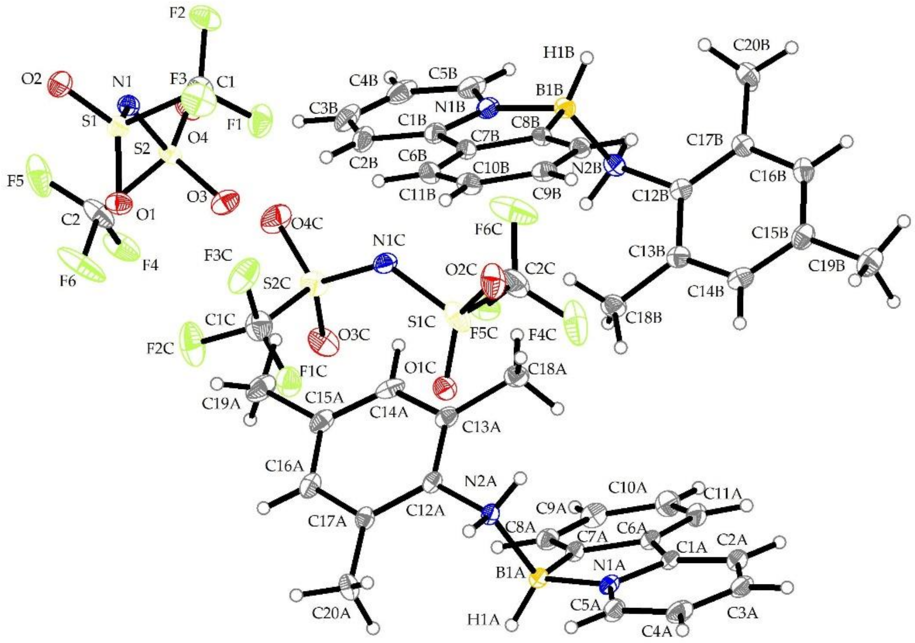

Figure S19: Crystal structure of **4NH** (thermal ellipsoids shown at 30 % probability level).

Synthesis of 6

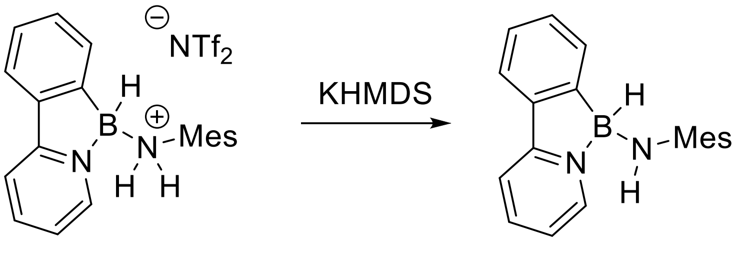

4NH **6**

Compound **4NH** (257 mg, 0.44 mmol) was dissolved in anhydrous toluene (10 mL) and potassium bis(trimethylsilyl)amide (KHMDS, 88.2 mg, 1 equiv.) was added. The mixture was stirred at room temperature for 2 hours. Then the solvent was evaporated and the residue redissolved in chloroform (10 mL). Precipitated salts were removed by filtration through a PTFE microfilter and the obtained solution concentrated *in vacuo*. After drying, **6** was obtained in the form of yellow powder (130 mg, yield: 98 %).

Characterization of **6**

NMR (CD_2Cl_2, 20 °C): ^{1}H (600 MHz): δ 2.11 (s, 6H), 2.21 (s, 3H), 4.02 (d, $^3J_{HH}$ = 133.8 Hz, 1H, B-*H*), 6.76 (s, 2H), 7.27 (ddd, $^3J_{HH}$ = 7.1, 5.6 Hz; $^4J_{HH}$ = 1.3 Hz, 1H), 7.36 (td, $^3J_{HH}$ = 7.5 Hz, $^4J_{HH}$ = 1.2 Hz, 1H), 7.43 (td, $^3J_{HH}$ = 7.2 Hz, $^4J_{HH}$ = 1.1 Hz, 1H), 7.70 (d, $^3J_{HH}$ = 7.2 Hz, 1H), 7.85 (dt, $^3J_{HH}$ = 7.6 Hz, $^4J_{HH}$ = 1.0 Hz, 1H), 7.95 (dt, $^3J_{HH}$ = 8.1 Hz, $^4J_{HH}$ = 1.1 Hz, 1H), 7.99 (td, $^3J_{HH}$ = 7.7 Hz, $^4J_{HH}$ = 1.5 Hz, 1H), 8.16 (dt, $^3J_{HH}$ = 5.7 Hz, $^4J_{HH}$ = 1.3 Hz, 1H);

^{13}C{^{1}H} (151 MHz): δ 19.8, 20.6, 118.2, 121.5, 122.3, 126.8, 127.6, 128.5, 129.4, 130.9, 136.9, 140.9, 143.6, 146.6, 146.5, 156.1, 158.1 (br, *C*-B);

^{11}B (193 MHz): δ 0.2 (d, $^1J_{BH} \approx$ 100 Hz); ^{11}B{^{1}H} (193 MHz): δ 0.2 (s);

IR (KBr): **3363 (m, NH)**, 3042 (w), 2908 (m), 2854 (m), 2734 (w), **2379 (s, B-H)**, 1615 (s), 1570 (w), 1561 (w), 1481 (s), 1439 (m), 1386 (m), 1326 (m), 1298 (m), 1287 (w), 1267 (w), 1244 (m), 1230 (m), 1172 (m), 1160 (w), 1111 (m), 1081 (w), 1064 (m), 1044 (w), 1007 (m), 976 (w), 94 (w), 927 (w), 859 (m), 824 (w), 766 (s), 741 (s), 716 (w), 689 (m), 654 (m), 605 (m), 585 (m), 568 (m), 507 (w), 487 (w), 457 (w), 419 (w) cm^{-1}.

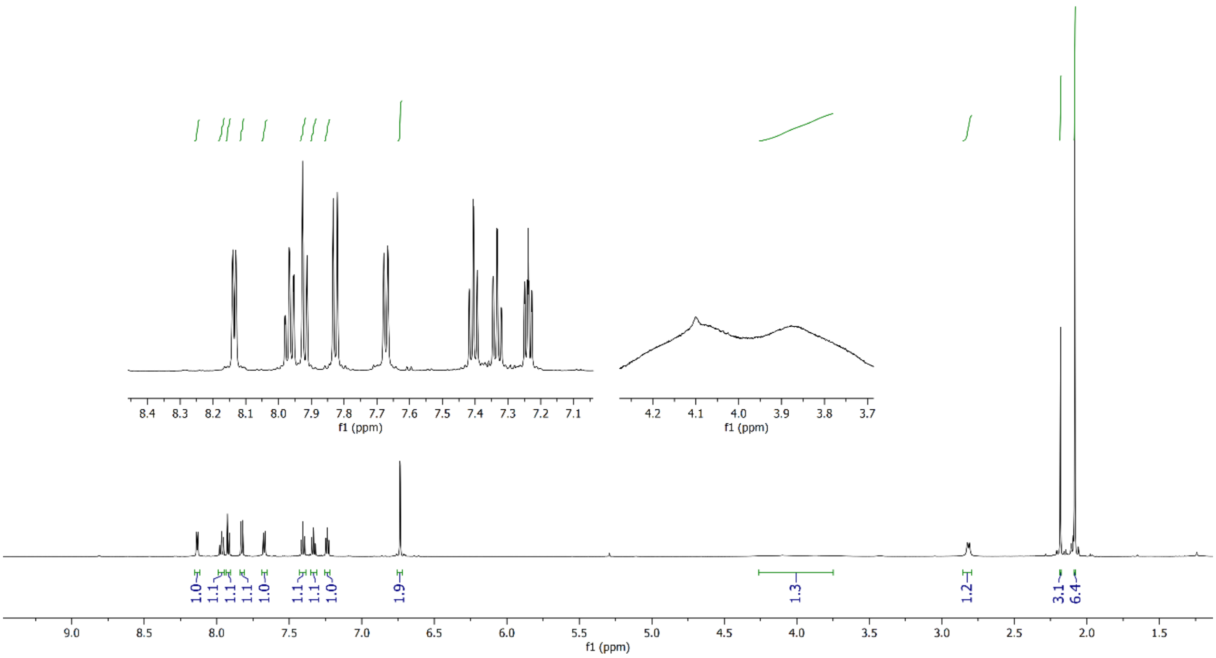

Figure S20: ^{1}H NMR (600 MHz, CD_2Cl_2, 20 °C) spectrum of compound **6**.

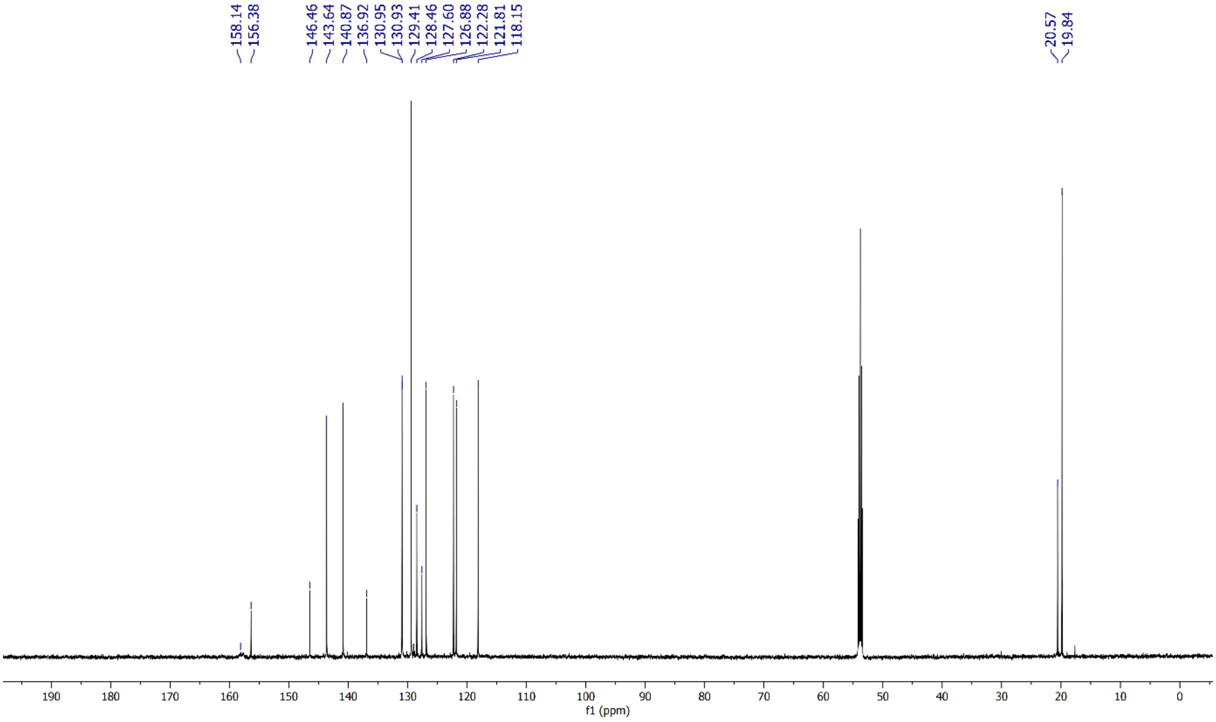

Figure S21: $^{13}C\{^{1}H\}$ NMR (151 MHz, CD_2Cl_2, 20 °C) spectrum of compound **6**.

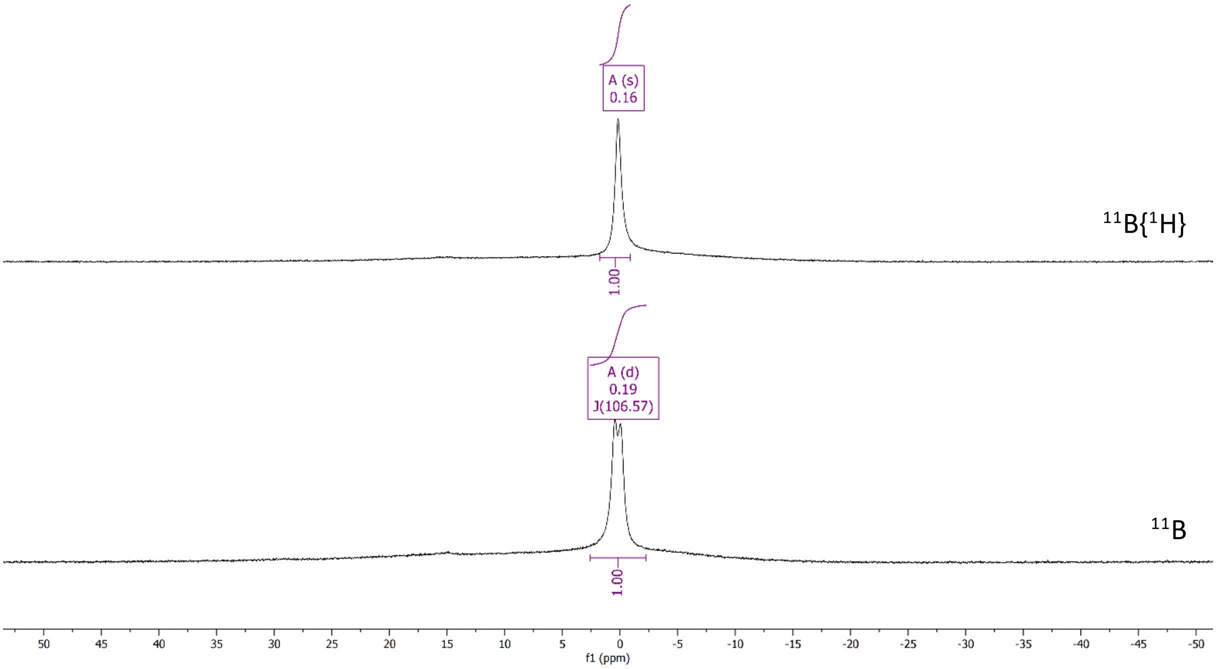

Figure S22: ^{11}B and $^{11}B\{^1H\}$ NMR (193 MHz, CD_2Cl_2, 20 °C) spectra of compound **6**.

Attempted hydride abstraction from compound 6.

Abstraction by trityl cation

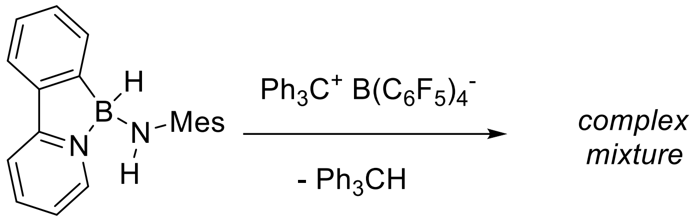

6

To a solution of compound **6** (14.6 mg, 0.049 mmol) in 0.3 mL CD_2Cl_2 in Young NMR tube was added triphenylmethylium tetrakis(pentafluorophenyl) borate (44.9 mg, 0.049 mmol, 1.0 equiv.) in 0.3 mL CD_2Cl_2. The reaction progress was directly montitored by ^{1}H and ^{11}B NMR and revealed formation of an undefined mixture of products according to both ^{1}H and ^{11}B NMR (see spectra below).

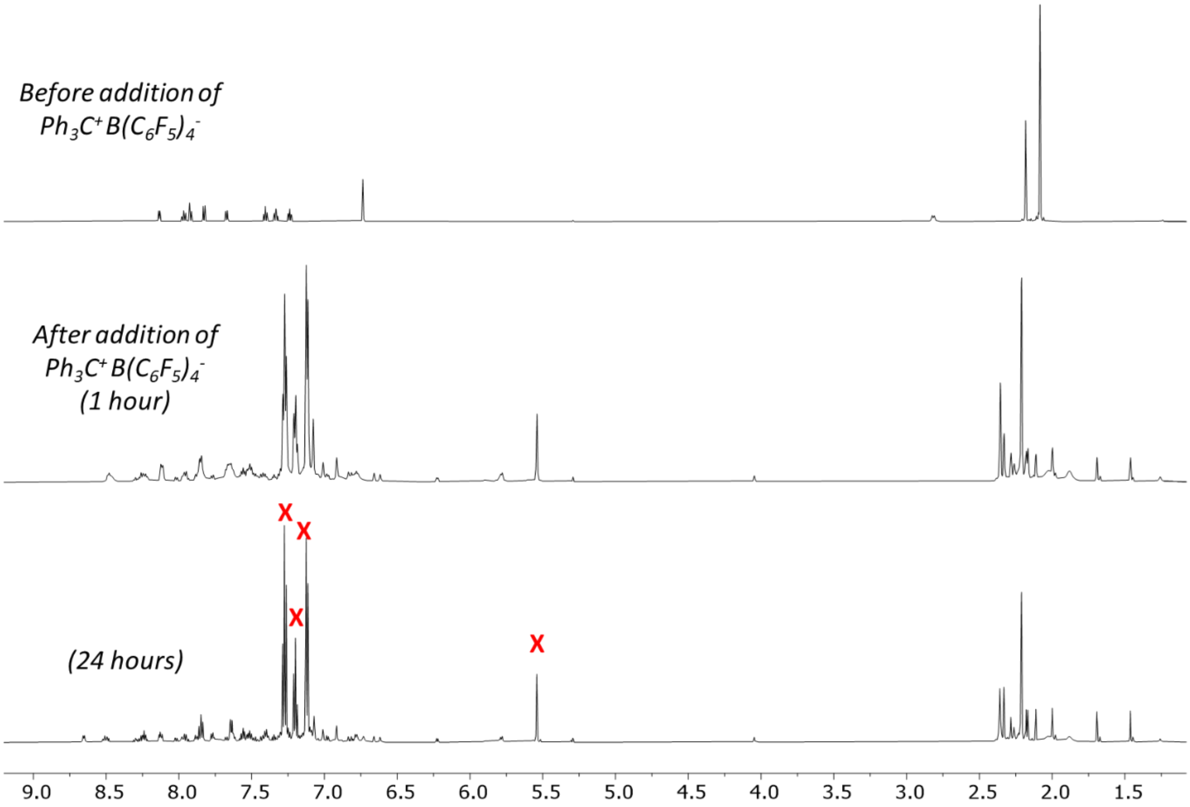

Figure S23: ^{1}H NMR (600 MHz, CD_2Cl_3, 20 °C) spectra of attempted hydride abstraction from compound **6**. (X denotes Ph_3CH as the sole identified product in the reaction mixture)

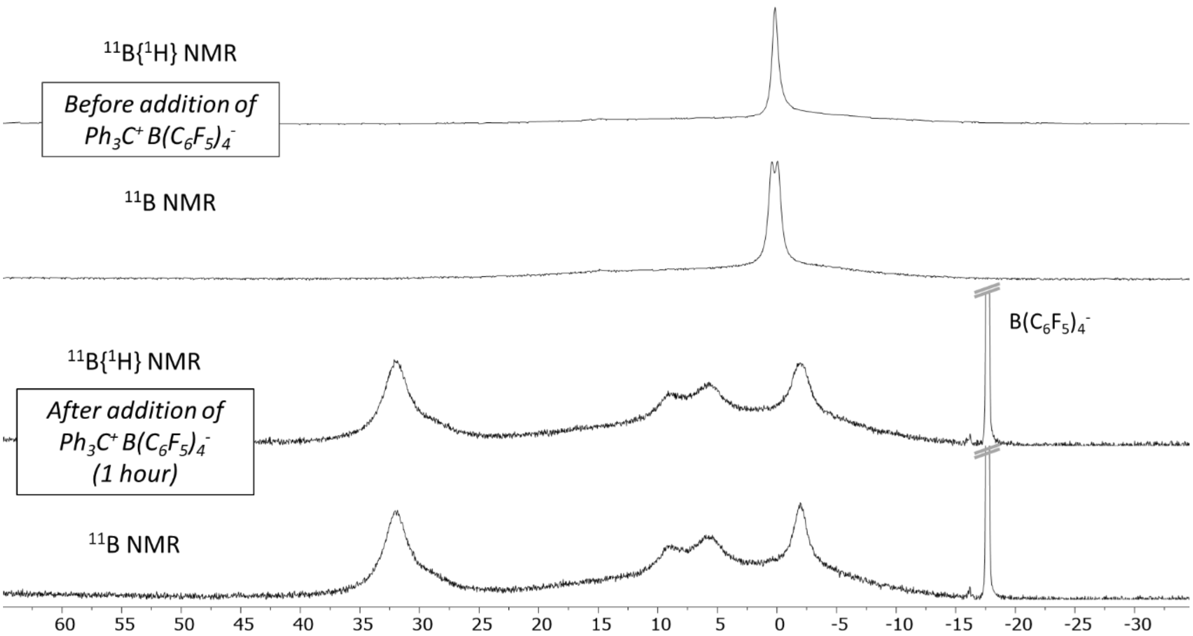

Figure S24: ^{11}B and $^{11}B\{^{1}H\}$ NMR (193 MHz, CD_2Cl_3, 20 °C) spectra of attempted hydride abstraction from compound **6**.

Reaction with triflimic acid

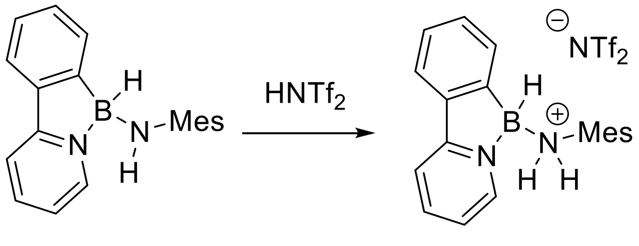

6

4NH

To a solution of compound **6** (16.0 mg, 0.053 mmol) in 0.4 mL CD_2Cl_2 in Young NMR tube was added bistriflimide (15.0 mg, 1.0 equiv.) in 0.2 mL CD_2Cl_2. The reaction resulted in reverse formation of compound **4NH** and its quantitative formation was observed.

Synthesis of 7

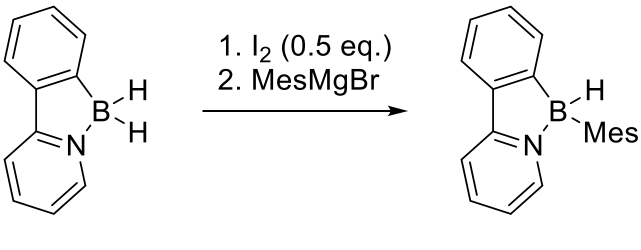

1 **7**

To a stirred solution of **1** (334 mg, 2.0 mmol) in anhydrous toluene (5 mL), solution of iodine (254 mg, 1 mmol, 0.5 equiv.) in 10 mL toluene was added dropwise. Upon addition, release of hydrogen gas was observed, together with the discoloration of iodine in solution. After addition, the mixture was stirred for 30 minutes at room temperature. The yellow suspension was then cooled to –78 °C using ethanol/liquid nitrogen bath. After cooling, solution of MesMgBr (1.0M in Et_2O, 2.2 mL, 1.1 equiv.) was added dropwise and the mixture was cooled for additional 30 minutes. The solution was then allowed to warm to room temperature and stirred overnight. The next day the reaction was quenched with methanol (2 mL), volatiles were removed *in vacuo* and the mixture was purified by column chromatography on silica gel using DCM/hexane 1/1 (v/v) as mobile phase. Combined fractions containing the product were evaporated, yielding **7** in the form of white solid (330 mg, yield: 58 %).

Characterization of **7**

Elemental analysis calculated for $C_{20}H_{20}NB$ (285.2): C 84.23, H 7.07, N 4.91; found C 84.05, H 7.24, N 5.29;

NMR ($CDCl_3$, 20 °C): ^{1}H (600 MHz): δ 1.17 (s, 3H), 2.30 (s, 3H), 2.79 (s, 3H), 4.49 (br m, 1H, B-*H*), 6.63 (s, 1H), 7.01 (s, 1H), 7.27 (dd, $^3J_{HH}$ = 7.3, 5.7 Hz, 1H), 7.36 (dd, $^3J_{HH}$ = 7.8 Hz, 1H), 7.43 (dd, $^3J_{HH}$ = 7.8 Hz, 1H), 7.65 (d, $^3J_{HH}$ = 7.5 Hz, 1H), 7.94 (d, $^3J_{HH}$ = 7.7 Hz, 1H), 7.98 (dd, $^3J_{HH}$ = 7.4 Hz, 1H), 8.04 (d, $^3J_{HH}$ = 7.8 Hz, 1H), 8.35 (d, $^3J_{HH}$ = 5.7 Hz, 1H);

^{13}C{^{1}H} (151 MHz): δ 21.1, 21.4, 25.4, 118.1, 121.3, 121.8, 125.3, 128.2, 128.4, 130.0, 130.7, 135.7, 136.2, 139.4, 141.4 (br, C-B), 142.9, 143.6, 144.6, 158.2, 163.4 (br, C-B);

^{11}B (193 MHz): δ –4.3 (d, $J_{BH} \approx 95$ Hz); ^{11}B{^{1}H} (193 MHz): δ –4.3 (br s);

IR (KBr) ν: 3055 (w), 3016 (w), 2968 (m), 2941 (m), 2912 (m), 2850 (w), **2333 (s, B-H)**, 1619 (m), 1600 (m), 1554 (m), 1477 (s), 1441 (s), 1417 (m), 1371 (w), 1322 (m), 1284 (w), 1267 (w), 1189 (w), 1160 (m), 1129 (w), 1082 (w), 1061 (m), 1016 (m), 995 (m), 946 (w), 884 (w), 851 (m), 778 (s), 744 (s), 686 (m), 663 (m), 590 (m), 528 (w), 433 (w) cm^{-1}.

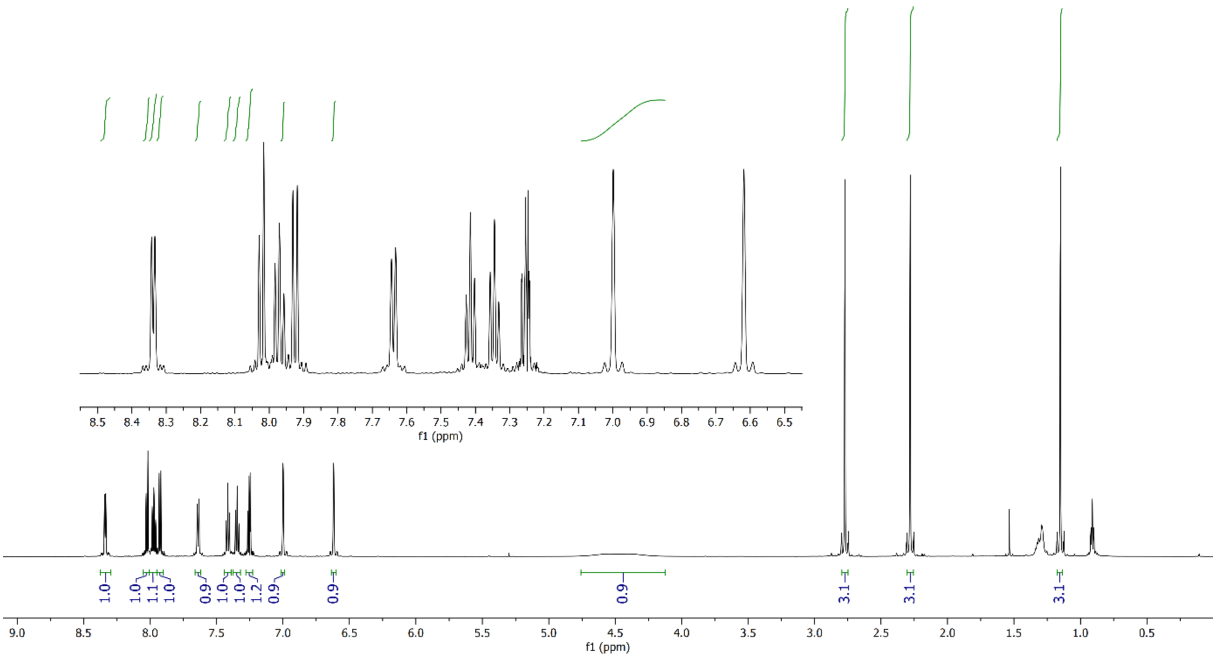

Figure S25: ^{1}H NMR (600 MHz, $CDCl_3$, 20 °C) spectrum of compound **7**.

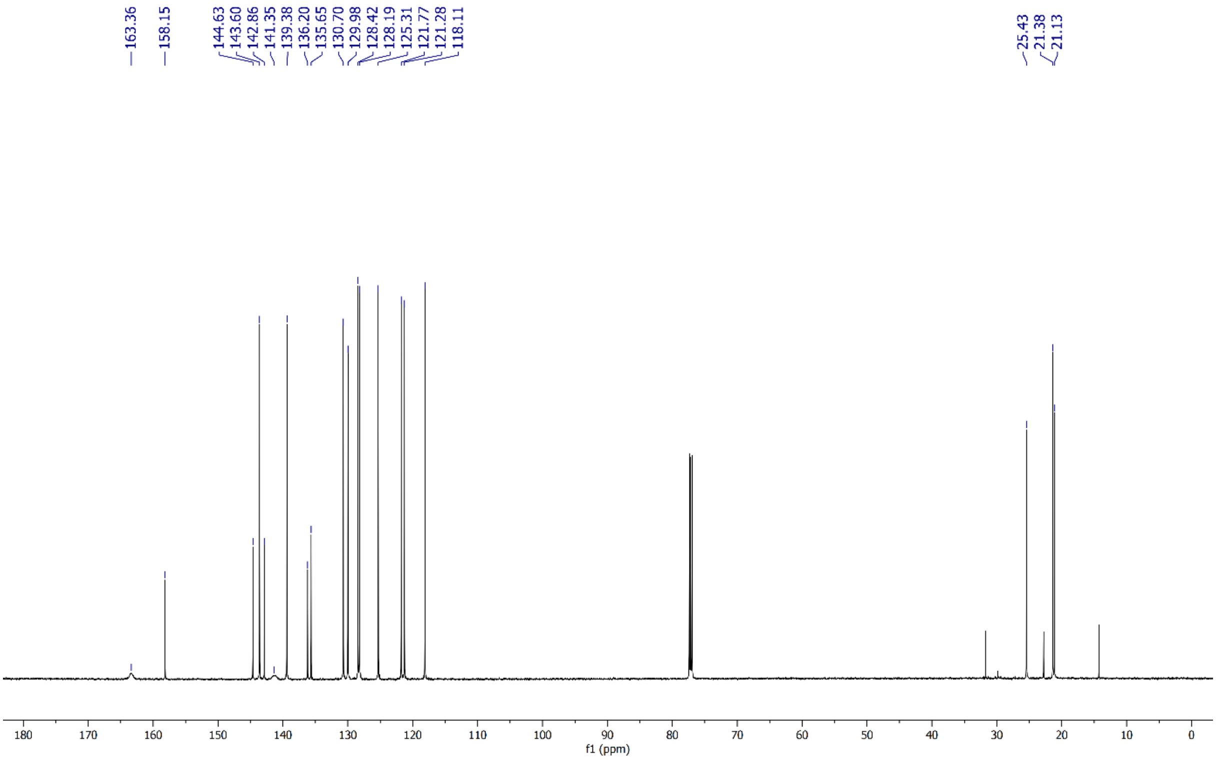

Figure S26: ^{13}C{^{1}H} NMR (151 MHz, $CDCl_3$, 20 °C) spectrum of compound **7**.

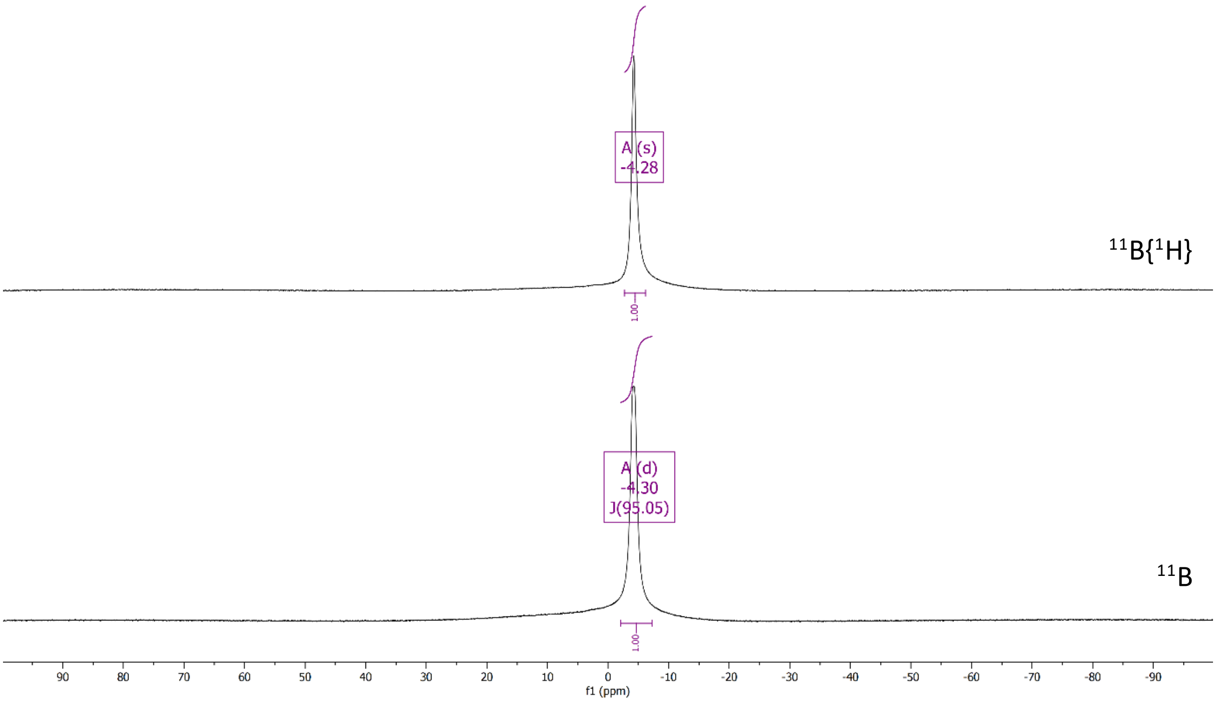

Figure S27: ^{11}B and $^{11}B\{^{1}H\}$ NMR (193 MHz, $CDCl_3$, 20 °C) spectra of compound **7**.

Crystal structure determination of 7 [KSK336-gg]

Suitable crystal for SC-XRD structure determination was obtained by slow cooling of a hot solution of **7** in heptane/chloroform mixture (ca 5/1) to room temperature.

Crystal Data for $C_{20}H_{20}BN$ (M =285.18 g/mol): orthorhombic, space group $P2_12_12_1$ (no. 19), a = 9.7533(6) Å, b = 11.6093(6) Å, c = 14.2424(11) Å, V = 1612.65(18) Å^3, Z = 4, T = 99.99(10) K, μ(Cu $K\alpha$) = 0.502 mm^{-1}, D_{calc} = 1.175 g/cm^3, 6411 reflections measured (9.83° ≤ 2Θ ≤ 154.146°), 2872 unique (R_{int} = 0.0529, R_{sigma} = 0.0632) which were used in all calculations. The final R_1 was 0.0432 ($I > 2\sigma(I)$) and wR_2 was 0.1051 (all data). CCDC Deposition Number: **2488310**.

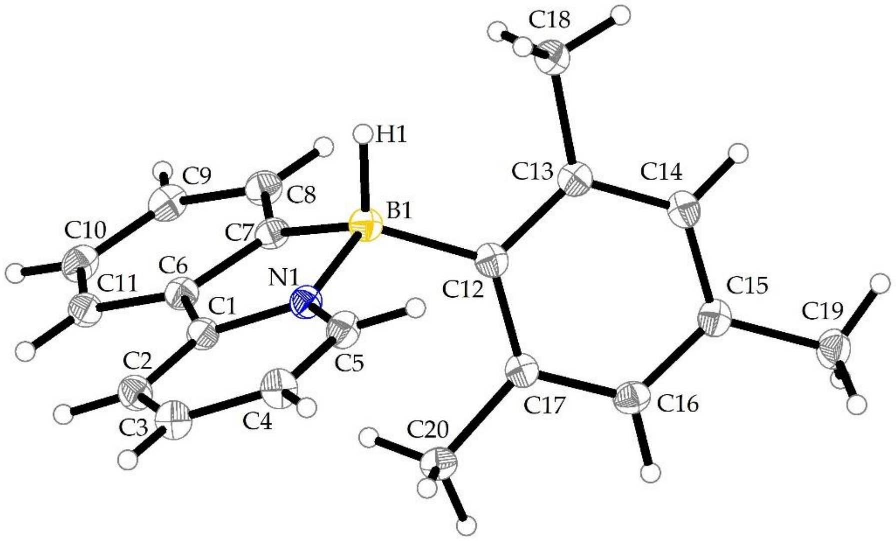

Figure S28: Crystal structure of **7** (thermal ellipsoids shown at 30 % probability level).

Synthesis of 8

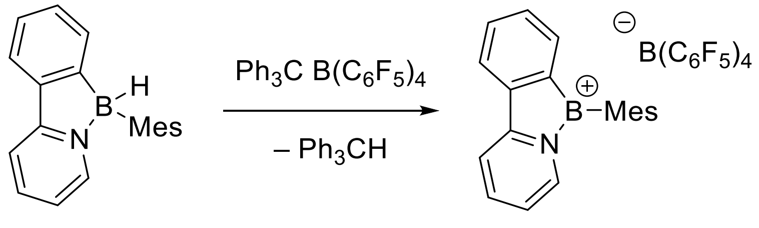

7 8

To a solution of **7** (66.9 mg, 0.235 mmol) in DCM (5 mL), a solution of triphenylmethylium tetrakis(pentafluorophenyl) borate (195 mg, 0.9 equiv.) in 5 mL DCM was added and the mixture was stirred for 10 minutes at room temperature. Then the solvent was evaporated, the residue dissolved in minimum amount of DCM (1 mL) and triturated with hexane (5 mL). This procedure was repeated twice and the residue was then washed with hexane (3x 5 mL) and dried *in vacuo*. Product **8** was obtained in the form of bright yellow powder (185 mg, yield: 82 %). Lower yield is most probably caused by losses in the trituration process employing DCM, in which the compound is highly soluble.

Characterization of **8**

Elemental analysis calculated for $C_{44}H_{19}B_2F_{20}N$ (963.23): C 54.89, H 1.99, N 1.45; found C 53.62, H 2.15, N 1.49;

NMR ($CDCl_3$, 20 °C): ^{1}H (600 MHz): δ 2.22 (s, 6H), 2.22 (s, 3H), 7.02 (s, 2H), 7.63 (t, $^3J_{HH}$ = 7.4 Hz, 1H), 7.68 (m, 1H), 7.78 (d, $^3J_{HH}$ = 7.5 Hz, 1H), 7.83 (d, $^3J_{HH}$ = 7.6 Hz, 1H), 7.93 (d, $^3J_{HH}$ = 7.2 Hz, 1H), 8.07 (d, $^3J_{HH}$ = 7.9 Hz, 1H), 8.30 (dd, $^3J_{HH}$ = 5.9, $^4J_{HH}$ = 1.4 Hz, 1H), 8.52 (td, $^3J_{HH}$ = 7.8, $^4J_{HH}$ = 1.6 Hz, 1H);

^{11}B (193 MHz) δ 61.3 (br s), –17.7 (s, BAr^F); ^{11}B{^{1}H} ($CDCl_3$, 193 MHz) δ 61.3 (br s), –17.7 (s, BAr^F);

^{19}F (565 MHz): δ –132.5 (s, 2F), –162.5 (s, 1F), –166.5 (s, 2F);

^{13}C (151 MHz): 21.4, 23.1, 120.8, 123.8 (br, *i*-C_6F_5), 124.6, 126.5, 129.1, 133.6, 135.2, 136.2 (br d, $^1J_{FC}$ ≈ 248 Hz, C_6F_5), 138.1 (br d, $^1J_{FC}$ ≈ 245 Hz, C_6F_5), 139.1, 139.6, 140.9, 141.7, 143.9, 146.6, 148.1 (d, $^1J_{FC}$ = 240 Hz, C_6F_5), 153.8, 159.9; neither C-B signal was observed;

HRMS (ESI): *m/z* [M]$^+$ calculated for $C_{20}H_{19}BN$: 284.1605; found: 284.1632;

HRMS (ESI): *m/z* [M]$^-$ calculated for $C_{24}BF_{20}$: 678.9779; found: 678.9763.

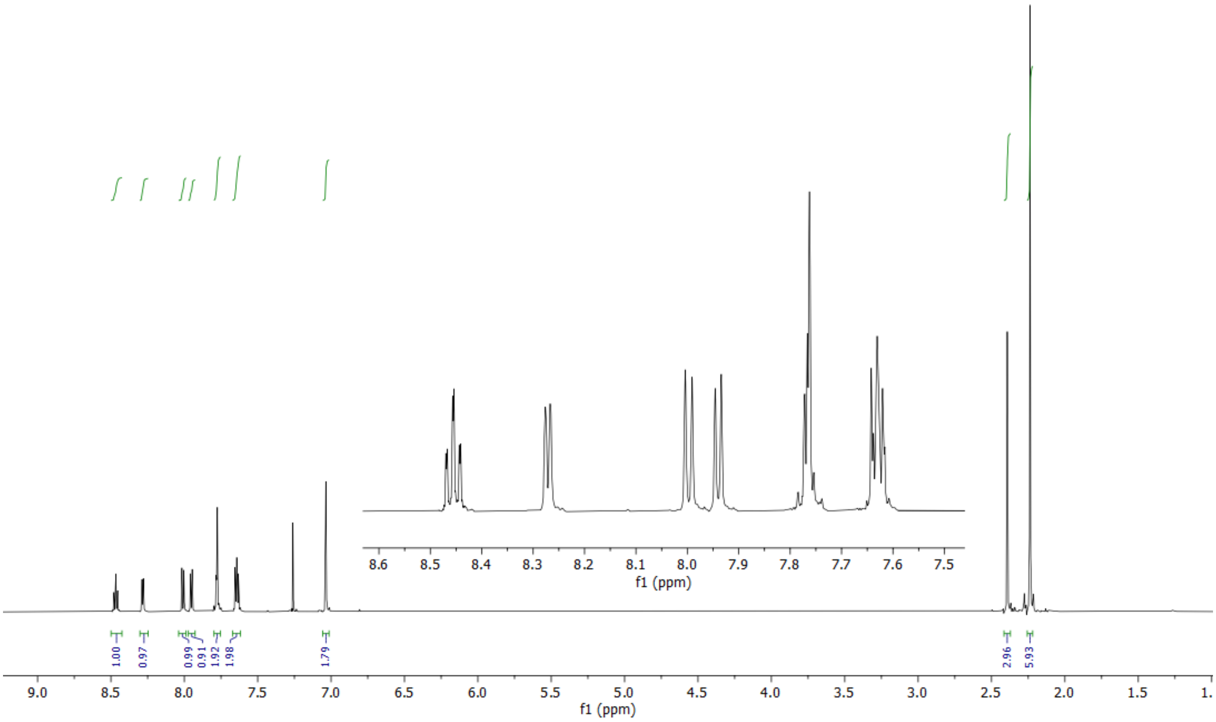

Figure S29: ^{1}H NMR (600 MHz, $CDCl_3$, 20 °C) spectrum of compound **8**.

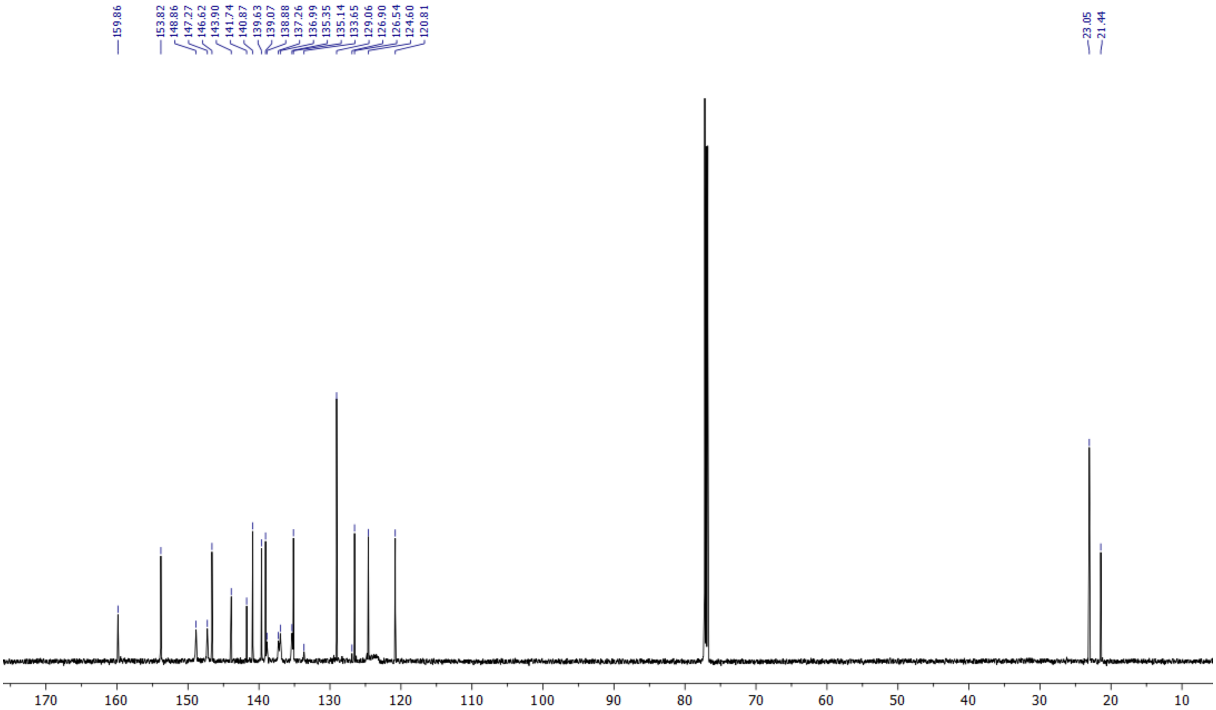

Figure S30: ^{13}C{^{1}H} NMR (151 MHz, $CDCl_3$, 20 °C) spectrum of compound **8**.

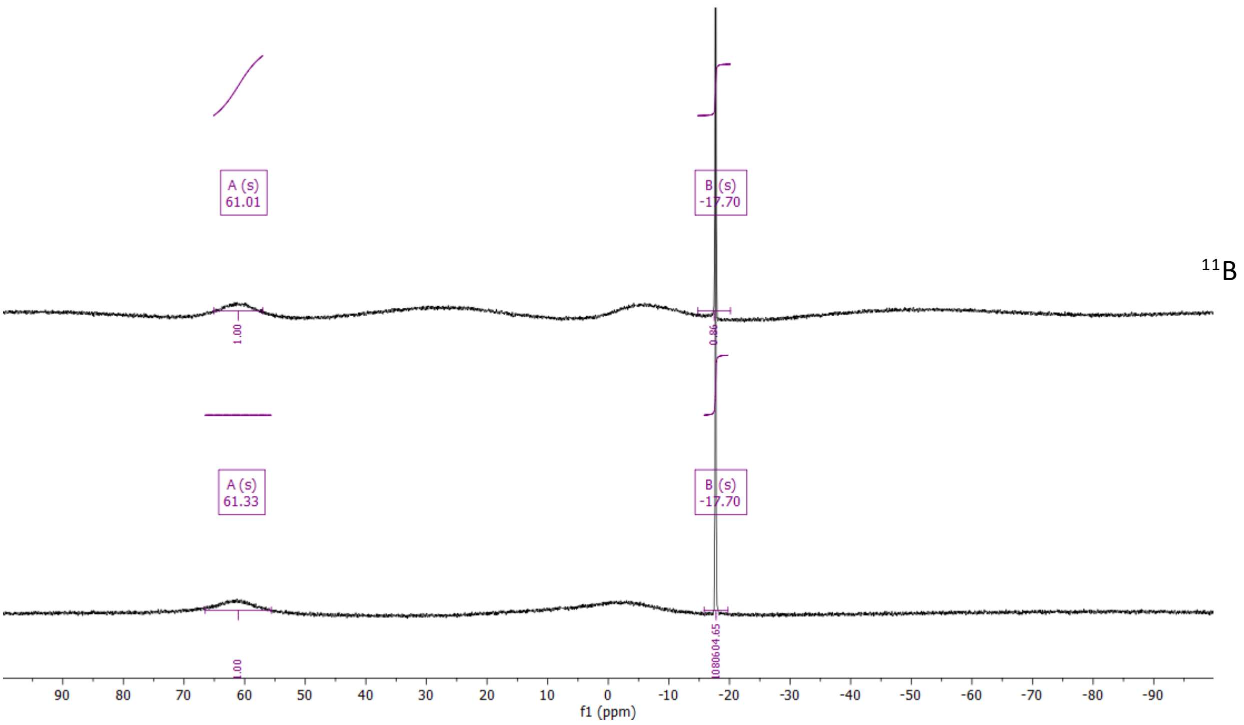

Figure S31: ^{11}B and $^{11}B\{^1H\}$ NMR (193 MHz, $CDCl_3$, 20 °C) spectra of compound **8**.

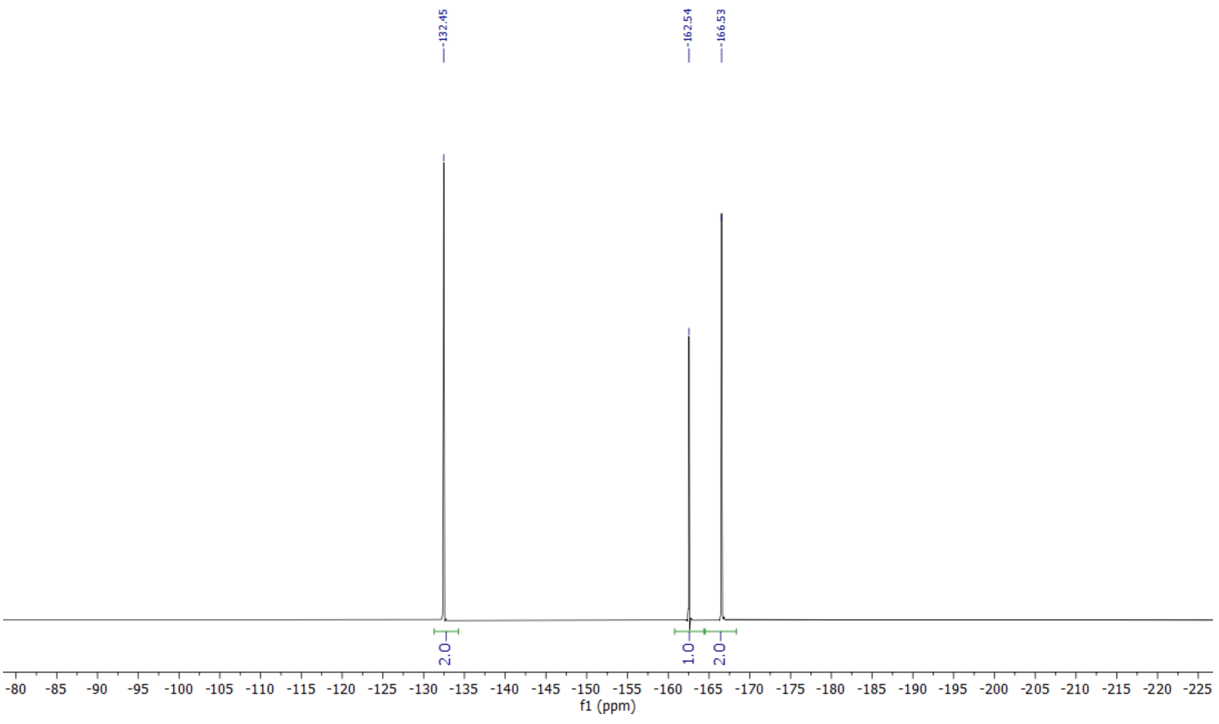

Figure S32: ^{19}F NMR (564 MHz, $CDCl_3$, 20 °C) spectrum of compound **8**.

Crystal structure determination of 8 [KSK361-P1-gg]

Suitable crystal for SC-XRD structure determination was obtained by diffusion of hexane into dichloromethane solution of **8** at room temperature.

Crystal Data for $C_{44}H_{19}B_2F_{20}N$ (M =963.22 g/mol): triclinic, space group $P1$ (no. 1), a = 11.4222(3) Å, b = 12.4087(3) Å, c = 14.8004(4) Å, α = 102.606(2)°, β = 106.093(2)°, γ = 90.935(2)°, V = 1960.33(9) Å^3, Z = 2, T = 100.00(10) K, μ(Cu $K\alpha$) = 1.447 mm^{-1}, D_{calc} = 1.632 g/cm^3, 59326 reflections measured (6.39° ≤ 2Θ ≤ 156.304°), 14462 unique (R_{int} = 0.0513, R_{sigma} = 0.0368) which were used in all calculations. The final R_1 was 0.0321 ($I > 2\sigma(I)$) and wR_2 was 0.0817 (all data). CCDC Deposition Number: **2488311**.

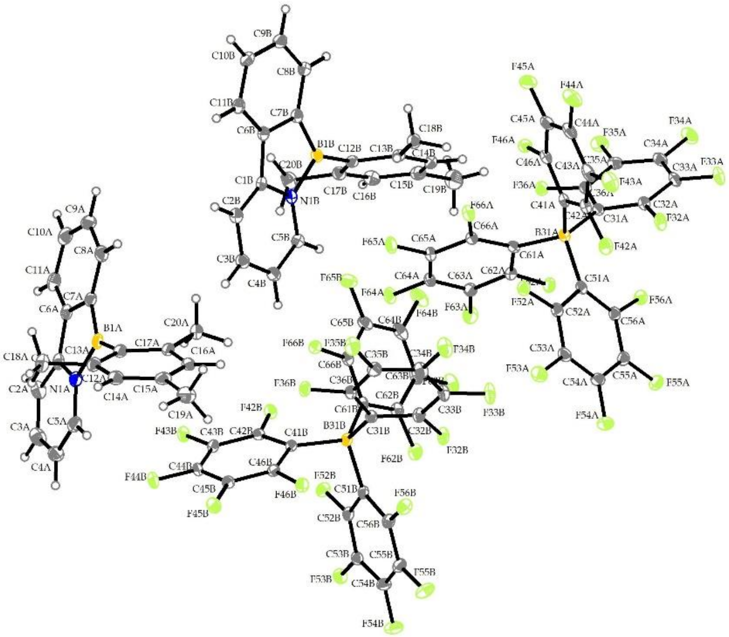

Figure S33: Crystal structure of **8** (thermal ellipsoids shown at 30 % probability level).

Determination of Buried Volume

The buried volumes were determined using the SambVca program[S14] based on the geometries of the corresponding fluoride adducts obtained for the FIA calculations at the PW6B95(D3)/def2-QZVPP//PBE0(D3)/def2-TZVP level of theory. The center of the 3.5 Å sphere was placed on the fluorine atom, and scaled radii (1.17) were employed according to the standard SambVca 2.1 protocol.

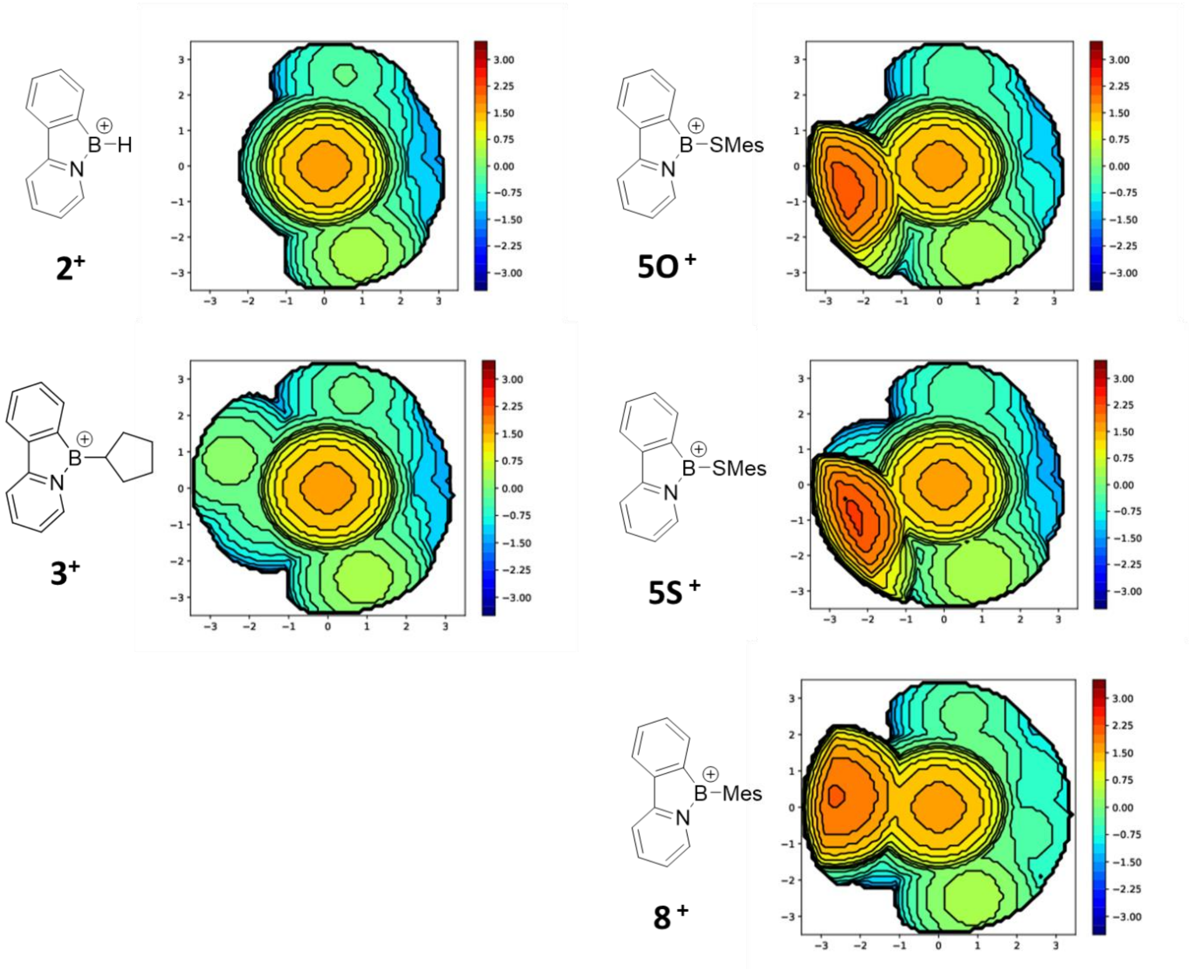

Figure S34: Topographic steric maps of the corresponding borenium cations as determined by SambVca 2.1.

Preparation of hydrazones and diazomethanes
Preparation of hydrazones

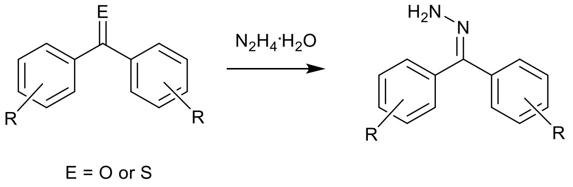

General method A:

Stirred solution of corresponding ketone/sulphide in absolute ethanol (30 mL) was treated with excess hydrazine monohydrate (typically ~5 eq.) and the mixture was refluxed overnight. In some cases, catalytic amount of acetic acid (1–2 drops) was added to facilitate the reaction. After cooling to room temperature, volatiles were evaporated and the crude product was either purified by column chromatography on silica gel or used directly in the next step without further purification.

Method B:

10,11-Dihydro-dibenzo[a,d]cycloheptene-5-thione was prepared by refluxing the ketone (1 equiv.) with excess P_2S_5 (4 equiv.) in toluene[S15] and consequent chromatography on silica gel, using Hexane/DCM 1/1 (v/v) as eluent, collecting the coloured band. The corresponding hydrazone was prepared by subsequent reaction of the sulphide with excess hydrazine monohydrate in chloroform for 45 mins at room temperature.[S16]

Preparation of diazomethanes

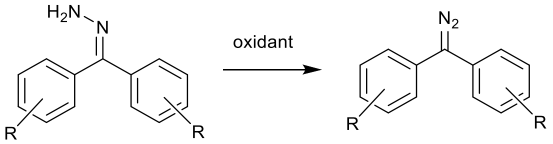

Diazo compounds were prepared by oxidation of the corresponding hydrazones using either activated manganese(IV) oxide (MnO_2) or yellow mercury(II) oxide (HgO) as the oxidizing agent.

MnO_2 pathway: To a stirred solution of the hydrazone (1.0 equiv.) in anhydrous dichloromethane was added magnesium sulfate (3 equiv.) and the mixture was cooled to 0 °C in an ice bath. To the vigorously stirred mixture, activated manganese dioxide was added, the reaction was allowed to warm to room temperature and was then stirred for the designated amount of time. Corresponding reaction times and equivalents of oxidizing agent used are stated in the experimental section by the respective compounds.

HgO pathway: To a stirred solution of the hydrazone (1.0 equiv.) in anhydrous diethyl ether was added sodium sulfate (2 equiv.), yellow mercury oxide (2.4 equiv.) and the reaction was initiated by the addition of 1 mL of saturated KOH solution in absolute ethanol. The mixture was stirred vigorously at room temperature for 3 hours, filtered and concentrated *in vacuo*.

The crude product was then either purified by flash filtration through a column of basic aluminium oxide using diethyl ether as the eluent, collecting the coloured band of the diazo compound, or was pure enough to be used as obtained (purification method is stated by the respective compounds below).

Benzophenone hydrazone: colourless crystals[S17]

Prepared by General method A with acid, recrystallized from hot ethanol. Yield: 82 %

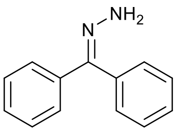

^{1}H NMR ($CDCl_3$, 600 MHz, 20 °C): δ 5.43 (s, 2H, NNH_2), 7.29 (m, 5H), 7.47 (m, 3H), 7.54 (m, 2H);

Diazobenzophenone (9a): dark purple solid[S18]

Prepared by the HgO pathway. Yield: 84 %

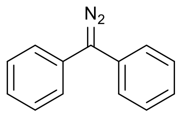

^{1}H NMR ($CDCl_3$, 600 MHz, 20 °C): δ 7.19 (t, $^{3}J_{HH}$ = 7.4 Hz, 2H), 7.30 (d, $^{3}J_{HH}$ = 7.8 Hz, 4H), 7.39 (d, $^{3}J_{HH}$ = 7.7 Hz, 4H);

^{13}C{^{1}H} ($CDCl_3$, 151 MHz, 20 °C): δ 62.5 ($C=N_2$), 125.3, 125.7, 129.2, 129.6.

IR (ATR): ν = 2032 cm^{-1} ($C=N_2$).

4,4'-Dimethyl-benzophenon-hydrazon: white crystals

Prepared by General method A with acid, recrystallized from hot ethanol. Yield: 85 %

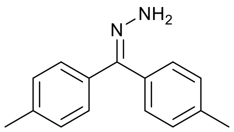

^{1}H NMR ($CDCl_3$, 600 MHz, 20 °C): δ 2.33 (s, 3H), 2.43 (s, 3H), 5.36 (s, 2H, NNH_2), 7.09 (d, $^{3}J_{HH}$ = 8.1 Hz, 2H), 7.17 (m, 2H), 7.33 (d, $^{3}J_{HH}$ = 7.8 Hz, 2H), 7.36 (m, 2H).

Bis(4-methylphenyl)diazomethane (9b): dark purple crystals[S18]

Prepared by the HgO pathway. After filtration and removal of the solvent, the residue was treated with hexane, which initiated the formation of dark-purple crystals of the product, which were isolated by filtration. Yield: 89 %

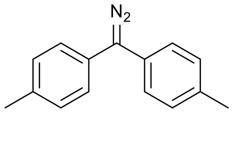

^{1}H NMR ($CDCl_3$, 600 MHz, 20 °C): δ 2.35 (s, 6H), 7.18 (m, 8H);

^{13}C{^{1}H} ($CDCl_3$, 151 MHz, 20 °C): δ 21.1, 61.8 ($C=N_2$), 125.3, 126.6, 129.9, 135.39.

IR (ATR): ν = 2029 cm^{-1} ($C=N_2$).

(Bis(4-bromophenyl)methylene)hydrazine: off-white solid[S19]

Prepared by General method A without acid. Yield: 88 %.

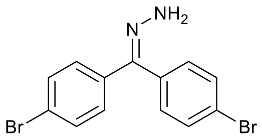

^{1}H NMR ($CDCl_3$, 600 MHz, 20 °C): δ 5.46 (s, 2H, NNH_2), 7.16 (d, $^{3}J_{HH}$ = 7.9 Hz, 2H), 7.30 (d, $^{3}J_{HH}$ = 8.3 Hz, 2H), 7.41 (d, $^{3}J_{HH}$ = 8.2 Hz, 2H), 7.68 (d, $^{3}J_{HH}$ = 7.9 Hz, 2H).

(Bis(4-bromophenyl)methylene)diazomethane (9d): purple solid[S18]

Prepared by the MnO_2 pathway. Conditions: 5 equiv. MnO_2, reaction time: 3 hours. Yield: 75 %

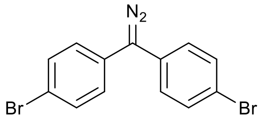

^{1}H NMR ($CDCl_3$, 600 MHz, 20 °C): δ 7.11 (m, 4H), 7.49 (m, 4H);

$^{13}C\{^1H\}$ ($CDCl_3$, 151 MHz, 20 °C): δ 62.2 ($C=N_2$)^a, 119.4, 126.7, 128.4, 132.5. ^{a} based on HMBC experiment

IR (ATR): ν = 2032 cm^{-1} ($C=N_2$).

(Bis(4-methoxyphenyl)methylene)hydrazine: white solid[S20]

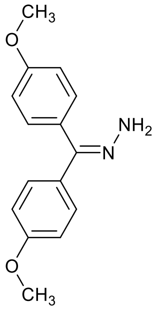

Prepared by General method A with acid. Yield: 85 %

After evaporation of the solvent and excess hydrazine monohydrate, the purity of the crude mixture which consisted of an inseparable mixture of E-/Z- isomers was 95 %, the impurity being the unreacted ketone. The mixture was used in the next reaction without further purification.

^{1}H NMR ($CDCl_3$, 600 MHz, 20 °C): δ 3.78 (s, 3H), 3.85 (s, 3H), 5.32 (s, 2H, NNH_2), 6.81 (m, 2H), 7.02 (m, 2H), 7.21 (m, 2H), 7.39 (m, 2H).

(Bis(4-methoxyphenyl)methylene)diazomethane (9c): purple solid[S20]

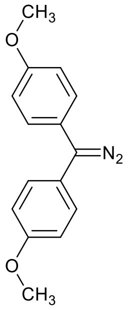

Prepared by the MnO_2 pathway. Conditions: 3 equiv. MnO_2, reaction time: 1 hour. After concentration of the filtrate *in vacuo*, product was purified by flash filtration through a column of basic aluminium oxide, eluted by Et_2O. Yield: 83 %. *Product purity is ~88 %, the impurity being the corresponding tetraphenyl ethylene, which is formed in small amounts during the oxidation reaction and cannot be separated due to the sensitive nature of the diazo product.*

^{1}H NMR ($CDCl_3$, 600 MHz, 20 °C): δ 3.81 (s, 6H), 6.93 (m, 4H), 7.17 (m, 4H);

$^{13}C\{^1H\}$ ($CDCl_3$, 151 MHz, 20 °C): δ 60.7 ($C=N_2$)^a, 114.9, 121.6, 126.6, 132.6, 157.8. ^{a} based on HMBC experiment

IR (ATR): ν = 2026 cm^{-1} ($C=N_2$).

(9H-fluoren-9-ylidene)hydrazine: light yellow solid[S21]

Prepared by General method A without acid. Used in the next reaction without further purification. Yield: 93 %

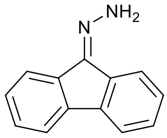

^{1}H NMR ($CDCl_3$, 600 MHz, 20 °C): δ 6.39 (s, 2H, NH_2), 7.27-7.39 (m, 3H), 7.43 (m, 1H), 7.64 (m, 2H), 7.72 (m, 1H), 7.76 (m, 1H), 7.90 (m, 1H).

9-Diazo-fluorene (9e): red solid[S18]

Prepared by the MnO_2 pathway. Conditions: 2.5 equiv. MnO_2, reaction time: 1 hour. After concentration of the filtrate *in vacuo*, product was purified by flash filtration through a column of basic aluminium oxide, eluted by Et_2O. Yield: 48 %.

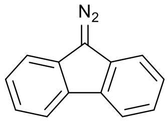

^{1}H NMR ($CDCl_3$, 600 MHz, 20 °C): δ 7.34 (td, J_{HH} = 7.5, 1.1 Hz, 2H), 7.40 (td, $^3J_{HH}$ = 7.5 Hz, $^4J_{HH}$ =1.2 Hz, 2H), 7.52 (d, $^3J_{HH}$ = 7.8 Hz, 2H), 7.96 (d, $^3J_{HH}$ = 7.7 Hz, 2H);

^{13}C{^{1}H} ($CDCl_3$, 151 MHz, 20 °C): δ 63.52 (C=N_2)^a, 119.4, 121.1, 124.6, 126.5, 131.6, 133.1. ^{a} based on HMBC experiment

IR (ATR): ν = 2048 cm^{-1} (C=N_2).

10,11-dihydro-5H-dibenzo[a,d][7]annulene-5-thione: dark blue oil[S22]

Thioderivative of dibenzosuberone was prepared following a published procedure. To a stirred solution of the ketone (1 equiv.) in toluene (50 mL), P_2S_5 (4 equiv.) was added at once and the suspension was refluxed overnight. The resulting mixture was then filtered, the filter was washed with hot toluene (3x15 mL) and the filtrate was evaporated *in vacuo*. The oily residue was then purified by column chromatography on silica gel using Hex/DCM 1/1 (v/v) as eluent. Only the coloured band was collected.

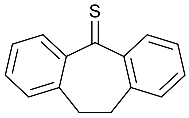

^{1}H NMR ($CDCl_3$, 600 MHz, 20 °C): δ 3.18 (s, 4H), 7.12 (dd, $^3J_{HH}$ = 7.6 Hz, $^4J_{HH}$ = 1.3 Hz, 2H), 7.21 (td, $^3J_{HH}$ = 7.6 Hz, $^4J_{HH}$ = 1.3 Hz, 2H), 7.36 (td, $^3J_{HH}$ = 7.4 Hz, $^4J_{HH}$ = 1.4 Hz, 2H), 7.77 (dd, $^3J_{HH}$ = 7.9 Hz, $^4J_{HH}$ = 1.4 Hz, 2H);

^{13}C{^{1}H} ($CDCl_3$, 151 MHz, 20 °C): δ 33.9, 126.4, 129.1, 129.6, 131.1, 136.5, 149.5, 246.8 (C=S).

10,11-dihydro-5H-dibenzo<a,d>cyclohepten-5-one hydrazone: yellow oil

Prepared by Method B. The crude hydrazone was purified by column chromatography on silica gel using Hexane/Ethyl acetate 3/1 (v/v) as the eluent. Yield: 74 %.

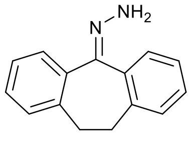

^{1}H NMR ($CDCl_3$, 600 MHz, 20 °C): δ 2.73-3.36 (br m, 4H), 5.56 (s, 2H, NNH_2), 7.08 (dd, $^3J_{HH}$ = 5.5, 3.6 Hz, 1H), 7.22 (m, 2H), 7.28 (m, 2H), 7.32 (td, $^3J_{HH}$ = 7.4 Hz; $^4J_{HH}$ = 1.6 Hz, 1H), 7.35 (dd, $^3J_{HH}$ = 7.5 Hz; $^4J_{HH}$ = 1.6 Hz, 1H), 7.39 (dd, $^3J_{HH}$ = 7.4 Hz; $^4J_{HH}$ = 1.5 Hz, 1H), 7.65 (m, 1H).

5-diazo-10,11-dihydro-5H-dibenzo[a,d]cycloheptene (9f): purple solid[S23]

Prepared by the MnO_2 pathway. Conditions: 3 equiv. MnO_2, reaction time: 2 hours. Used without further purification. Yield: 77 %

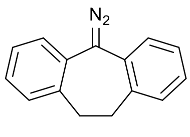

^{1}H NMR ($CDCl_3$, 600 MHz, 20 °C): δ 3.10 (s, 4H), 7.07 (td, $^3J_{HH}$ = 7.4 Hz; $^4J_{HH}$ = 1.3 Hz, 2H), 7.12 (dd, $^3J_{HH}$ = 7.9 Hz; $^4J_{HH}$ = 1.2 Hz, 2H), 7.16 (dd, $^3J_{HH}$ = 7.5 Hz; $^4J_{HH}$ = 1.5 Hz, 2H), 7.26 (td, $^3J_{HH}$ = 7.6 Hz; $^4J_{HH}$ = 1.5 Hz, 2H);

^{13}C{^{1}H} ($CDCl_3$, 151 MHz, 20 °C): δ 35.7, 64.0 (C=N_2)^a, 124.0, 124.9, 126.8, 128.06, 130.4, 139.6. ^{a} based on HMBC experiment

(2,2-Dimethyl-1-phenylpropylidene)hydrazine: colourless oily needles[S24]

Prepared by General method A without acid. The product was obtained in the form of colourless oil after evaporation of the volatiles. Upon standing, needle like oily crystals formed. The crude product was used in the next reaction without further purification. Yield: 90 %

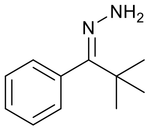

^{1}H NMR ($CDCl_3$, 600 MHz, 20 °C): δ 1.08 (s, 9H), 4.76 (s, 2H, NNH_2), 7.02 (m, 2H), 7.31 (m, 1H), 7.39 (m, 2H).

Tert-butyl(phenyl)diazomethane (9g): dark red oil[S19]

Prepared by the HgO pathway. Used without further purification. Yield: 52 %

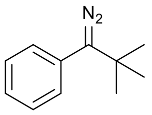

^{1}H NMR ($CDCl_3$, 600 MHz, 20 °C): δ 1.40 (s, 9 H), 1.08 (t, $^{3}J_{HH}$ = 7.4 Hz, 1H), 7.19 (d, $^{3}J_{HH}$ = 7.9 Hz), 7.34 (t, $^{3}J_{HH}$ = 7.8 Hz);

^{13}C{^{1}H} ($CDCl_3$, 151 MHz, 20 °C): δ 29.7, 62.8 (C=N_2)^{a}, 82.5, 123.8, 124.5, 128.8, 131.6. ^{a} based on HMBC experiment

IR (ATR): ν = 2029 cm^{-1} (C=N_2).

Non reactive substrates:

4-[(4-Cyanophenyl)methanehydrazonoyl]benzonitrile: off-white solid[S19]

Prepared by General method A with acid. Yield: 65 %

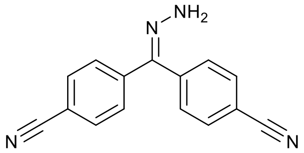

^{1}H NMR ($CDCl_3$, 600 MHz, 20 °C): δ 5.69 (s, 2H, NNH_2), 7.43 (d, $^{3}J_{HH}$ = 7.8 Hz, 2H), 7.49 (d, $^{3}J_{HH}$ = 8.1 Hz, 2H), 7.58 (d, $^{3}J_{HH}$ = 8.1 Hz, 2H), 7.88 (d, $^{3}J_{HH}$ = 7.7 Hz, 2H).

Di(4-cyanophenyl)diazomethane: red solid[S25]

Prepared by MnO_2 pathway. Conditions: 5 equiv. MnO_2, reaction time: 1 hour. Used without further purification. Yield: 92 %

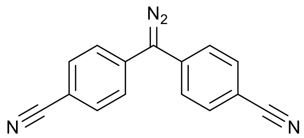

^{1}H NMR ($CDCl_3$, 600 MHz, 20 °C): δ 7.35 (m, 4H), 7.67 (m, 4H);

^{13}C{^{1}H} ($CDCl_3$, 151 MHz, 20 °C): δ 64.8 (C=N_2)^{a}, 109.4, 118.7 (CN), 125.1, 133.2, 134.1. ^{a} based on HMBC experiment.

IR (ATR): ν = 2045 cm^{-1} (C=N_2).

(Bis(3,5-bis(trifluoromethyl)phenyl)methylene)hydrazine: off-white solid

Prepared by modified General method A using 0.5 mL of concentrated HCl as the reaction catalyst. The crude mixture was purified by column chromatography on silica gel using Hexane/DCM 1/1 (v/v) as mobile phase. Yield: 61 %.

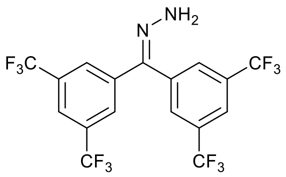

^{1}H NMR ($CDCl_3$, 600 MHz, 20 °C): δ 5.76 (s, 2H, NNH_2), 7.81 (d, $^{4}J_{HH}$ = 1.7 Hz, 3H), 7.82 (d, $^{4}J_{HH}$ = 1.6 Hz, 2H), 8.07 (s, 1H);

^{13}C{^{1}H} ($CDCl_3$, 151 MHz, 20 °C): δ 121.9, 122.9 (q, $^{1}J_{CF}$ = 273 Hz, CF_3), 123.2 (q, $^{1}J_{CF}$ = 273 Hz, CF_3), 125.8, 129.5, 132.0 (q, $^{2}J_{CF}$ = 34 Hz, *C*CF_3), 133.5, 133.8 (q, $^{2}J_{CF}$ = 34 Hz, *C*CF_3), 139.5, 141.1;

^{19}F NMR (564 MHz, $CDCl_3$, 20 °C): δ –62.94 (s, 6F), –62.85 (s, 6F).

5,5'-(Diazomethylene)bis(1,3-bis(trifluoromethyl)benzene): red solid

Prepared by the MnO_2 pathway. Conditions: 5 equiv. MnO_2, reaction time: 3 hours. Used without further purification. Yield: 94 %

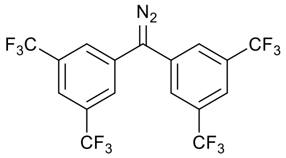

^{1}H NMR ($CDCl_3$, 600 MHz, 20 °C): δ 7.67 (s, 4H), 7.75 (s, 2H);

^{13}C{^{1}H} ($CDCl_3$, 151 MHz, 20 °C): δ 63.1 ($C{=}N_2$)^{a}, 120.2, 123.0 (q, $^{1}J_{CF}$ = 273 Hz, *C*F_3), 124.5, 131.4, 133.3 (q, $^{2}J_{CF}$ = 34.0 Hz, *C*CF_3). ^{a} based on HMBC experiment;

^{19}F NMR (564 MHz, $CDCl_3$, 20 °C): δ –63.13 (s, 12F).

IR (ATR): ν = 2052 cm^{-1} ($C{=}N_2$).

(4-Nitrophenyl)(phenyl)methylene)hydrazine: yellow oil

Prepared following General method A with acid, followed by extraction between Et_2O/water, drying and purification by column chromatography on silica gel using Hex/EtOAc 1/1 (v/v) as mobile phase. Yield: 72 %

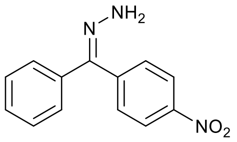

Overall purity of the obtained product was 90 %, the impurity probably being the other E-/Z-isomer.

^{1}H NMR ($CDCl_3$, 600 MHz, 20 °C): δ 5.75 (s, 2H, NNH_2), 7.26 (m, 2H), 7.50 (m, 1H), 7.55 (m, 2H), 7.58 (m, 2H), 8.11 (m, 2H).

1-(Diazo(phenyl)methyl)-4-nitrobenzene: red oily solid[S18]

Prepared by the MnO_2 pathway. Conditions: 5 equiv. MnO_2, reaction time: 3 hours. After concentration of the filtrate *in vacuo*, product was obtained as red oil which solidified upon standing. Yield: 83 %

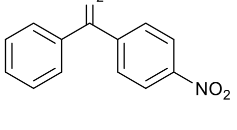

^{1}H NMR ($CDCl_3$, 600 MHz, 20 °C): δ 7.26 (m, 2H), 7.33 (m, 1H), 7.38 (m, 2H), 7.47 (m, 2H), 8.18 (m, 2H);

^{13}C{^{1}H} ($CDCl_3$, 151 MHz, 20 °C): δ 64.4 ($C{=}N_2$)^{a}, 123.0, 124.7, 127.2, 127.8, 129.7, 138.9, 144.4. ^{a} based on HMBC experiment.

IR (ATR): ν = 2044 cm^{-1} ($C{=}N_2$).

Catalytic evaluation

Solvent comparison study

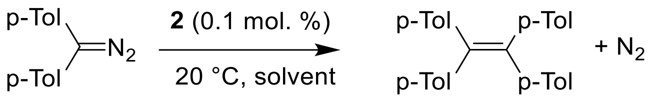

9b **10b**

λ_{max} = 535 nm

To identify the most suitable solvent for the homocoupling reaction, we studied the homocoupling of the bis(4-methylphenyl)diazomethane **9b** substrate using 0.1 mol % of the simplest borenium catalyst **2** with a hydride substituent in various solvents. The reaction progress was monitored by UV-Vis spectroscopy. Each sample preparation was conducted under inert atmosphere inside an argon-filled glovebox using anhydrous solvent. Reactions were assembled directly in 3mL quartz cuvettes with optical path of 1.00 cm.

Samples were prepared using the following general procedure: 500 μL of substrate **9b** stock solution (c = 0.06 M, c_F = 0.01 M) in corresponding solvent was transferred into 3mL quartz cuvette followed by 2.4 mL of the solvent. Then, 300 μL of catalyst **2** stock solution (c = 10^{-4} M, c_F = 10^{-5} M, V_F = 3 mL) prepared in respective solvent was added. For each solvent tested, a corresponding blank was prepared using the same catalyst concentration (300 μL of 10^{-4} M stock solution) in pure solvent (final volume 3 mL) to account for background absorbance.

After mixing, the cuvettes were sealed and promptly transported to the UV–Vis spectrometer. Spectra were recorded in the range of 400 to 700 nm every 5 minutes over a 90-minute period at a constant temperature of 20 °C. Catalyst performance was assessed by monitoring the decrease in absorbance at 535 nm (the absorption maximum of the substrate) over time.

Reaction kinetics were analysed by linear fitting of the natural logarithm of the absorbance at 535 nm (absorption maximum of the substrate) vs time and are shown in Figure S32. The observed rate constants are summarised in Table S1.

Table S1: Observed rate constants of the catalysed diazo-homocoupling reaction in various solvents. **Conditions**: c(substrate) = 10 mM, c(cat) = 0.01 mM, t = 0–90 min, T = 20 °C.

Solvent	k_{obs} ($\cdot 10^{-5}$ s^{-1})
**Hexane**	1.00
**Diethyl ether**	0.682
**Dichloromethane**	9.82
**Chloroform**	3.91
**Toluene**	2.36

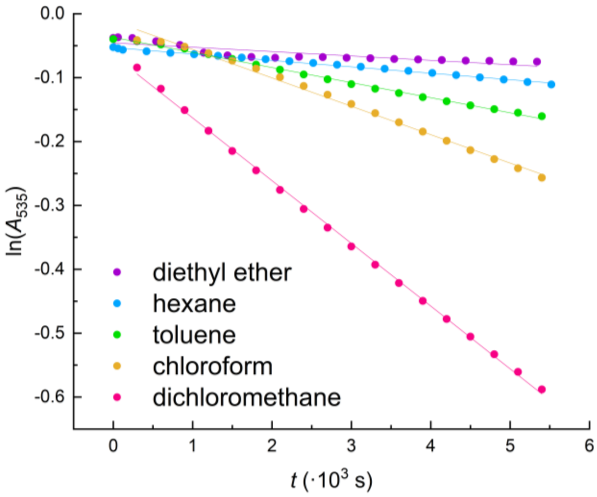

Figure S35: Time dependence of the natural logarithm of absorbance at 535 nm (substrate absorption maximum) for the homocoupling reaction of bis(4-methylphenyl)diazomethane catalyzed by **2** in various solvents. Conditions: c(substrate) = 10 mM, c(cat) = 0.01 mM, t = 0–90 min, T = 20 °C.

Catalyst screening

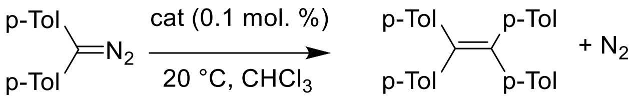

9b **10b**

λ_{max} = 535 nm

Catalyst screening was performed by monitoring the reaction of a selected substrate, bis(4-methylphenyl)diazomethane **9b**, in the presence of different catalyst candidates using UV–Vis spectroscopy. Each sample preparation was conducted under inert atmosphere inside an argon-filled glovebox, using anhydrous chloroform as the solvent. Reactions were assembled directly in 3mL quartz cuvettes with optical path of 1.00 cm.

Samples were prepared using the following general procedure: 500 µL of substrate **9b** stock solution (c = 0.06 M, c_F = 0.01 M) was transferred into the cuvette followed by 2.4 mL of chloroform. Then, 300 µL of catalyst stock solution ($c = 10^{-4}$ M, $c_F = 10^{-5}$ M, V_F = 3 mL) was added. For each catalyst tested, a corresponding blank was prepared using the same catalyst concentration (300 µL of 10^{-4} M stock solution) in pure chloroform (final volume 3 mL) to account for background absorbance.

After mixing, the cuvettes were sealed and promptly transported to the UV–Vis spectrometer. Spectra were recorded in the range of 400 to 700 nm every 5 minutes over a 5-hour period at a constant temperature of 20 °C. Catalyst performance was assessed by monitoring the decrease in absorbance at 535 nm (the absorption maximum of the substrate) over time.

Kinetic data were analysed by linear fitting of the natural logarithm of the absorbance in absorption maximum, to obtain observed rate constants and compare catalytic efficiencies. Tris(pentafluorophenyl)borane was also tested as a commercially available Lewis acid benchmark. The observed rate constants calculated for prepared catalyst series are summarised in Table S2. The kinetic profiles are shown in Figure S33.

Table S2: Observed rate constants of the catalyzed diazo-homocoupling reaction obtained by linear fitting of the natural logarithm of absorbance vs time. **Conditions**: *c*(substrate) = 10 mM, *c*(cat) = 0.01 mM, T = 20 °C, t = 0–5 h (**2**, **3**, **5O**, **12**, **13**, **14**, $B(C_6F_5)_3$; t = 0–2 h (**8**).

**Catalyst**	k_{obs} **(·10^{-5} s^{-1})**
**2**	3.91
**3**	5.17
**5O**	10.9
**8**	**15.4**
**12**	2.68
**13**	7.48
**14**	---
$B(C_6F_5)_3$	1.37

Based on these measurements, compound **8** [$L^{NC}BMes^+$ $B(C_6F_5)_4^-$] was identified as the most active catalyst and therefore it was used in the following substrate scope, which was done qualitatively using NMR spectroscopy.

Determination of rate constants in homocoupling of diazomethanes

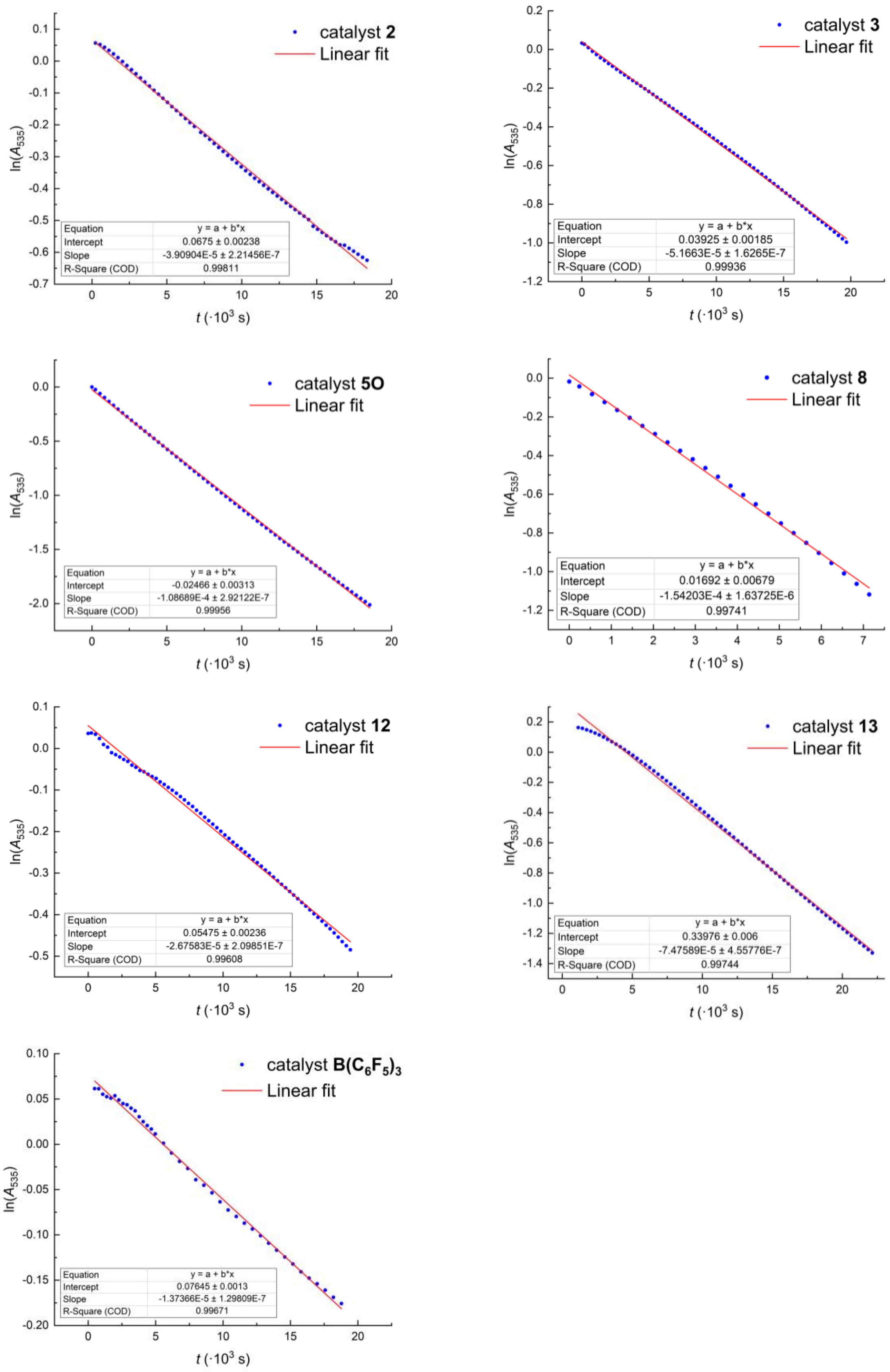

Figure S36: Time dependence of the natural logarithm of absorbance at 535 nm (absorption maximum of the substrate) for the diazo-homocoupling reaction catalysed by various catalysts in chloroform. Conditions: c(substrate) = 10 mM, c(cat) = 0.01 mM, t = 0–5 hod, T = 20 °C.

Characterization data of prepared tetraarylethylenes

The corresponding diazo compound (100 mg) was dissolved in $CHCl_3$ (10 mL) and transferred to a dried Schlenk flask under inert atmosphere. A solution of catalyst **8** (1 mol %) in chloroform (2 mL) was added, and the mixture was stirred overnight at room temperature/at 60 °C. As the reaction progressed, gradual discoloration of the diazo compound was observed. After completion, the resulting light-yellow solution was concentrated under reduced pressure and the crude residue was purified by column chromatography on silica gel (eluent composition and retention factors are provided for each respective product).

10a, 1,1,2,2-Tetraphenylethylene: white solid.[S26]

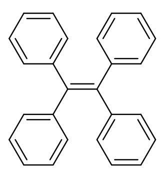

Eluent: Hexane/Ethyl acetate 15/1 (v/v), R_f = 0.79. Yield: 92 %

^{1}H NMR ($CDCl_3$, 600 MHz, 20 °C): δ 7.08 (m, 8H), 7.13 (m, 12H);

^{13}C{^{1}H} NMR ($CDCl_3$, 151 MHz, 20 °C): δ 126.54, 127.77, 131.46, 141.07, 143.85.

10b, 1,1,2,2-Tetrakis(4-methylphenyl)ethylene: white solid.[S26]

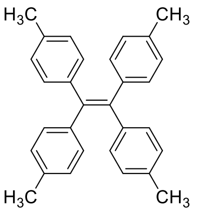

Eluent: Hexane/Ethyl acetate 95/5 (v/v), R_f = 0.70. Yield: 92 %.

^{1}H NMR ($CDCl_3$, 600 MHz, 20 °C): δ 2.25 (s, 12H), 6.89 (s, 16H);

^{13}C{^{1}H} NMR ($CDCl_3$, 151 MHz, 20 °C): δ 21.34, 128.48, 131.36, 135.79, 139.96, 141.48.

11b, 1,2-bis(di-4-tolylmethylene)hydrazine, yellow solid[S27]

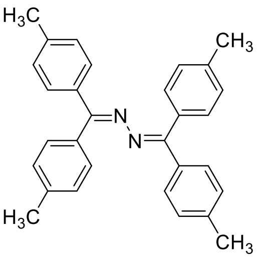

Eluent: Ethyl acetate 100%, R_f = 0.40.

^{1}H NMR ($CDCl_3$, 600 MHz, 20 °C): δ 2.37 (s, 3H), 2.40 (s, 3H), 7.12 (d, $^{3}J_{HH}$ = 7.95 Hz, 2H), 7.21 (d, $^{3}J_{HH}$ = 7.9 Hz, 2H), 7.27 (d, $^{3}J_{HH}$ = 8.1 Hz, 2H), 7.43 (m, 2H);

^{13}C{^{1}H} NMR ($CDCl_3$, 151 MHz, 20 °C): δ 21.5, 21.6, 128.5, 128.8, 128.9, 129.7, 132.8, 136.0, 138.5, 139.6, 158.99;

IR (ATR): ν 1608, 1583 cm^{-1}.

10c, 1,1,2,2-Tetrakis(4-methoxyphenyl)ethene, white solid[S28]

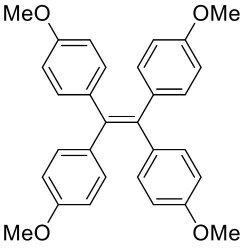

Eluent: dichloromethane, R_f = 0.79. Yield: 94 %

^{1}H NMR ($CDCl_3$, 600 MHz, 20 °C): δ 3.74 (s, 12H), 6.64 (d, $^{3}J_{HH}$ = 8.8 Hz, 8H), 6.93 (d, $^{3}J_{HH}$ = 8.7 Hz, 8H);

^{13}C{^{1}H} NMR ($CDCl_3$, 151 MHz, 20 °C): δ 55.21, 113.14, 132.68, 137.02, 138.48, 157.89.

10d, 1,1,2,2-Tetrakis(4-bromophenyl)ethene, white solid[S29]

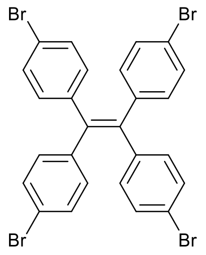

Eluent: Hexane/Ethyl acetate 10/1 (v/v). Yield: 87 %

^{1}H NMR ($CDCl_3$, 600 MHz, 20 °C): δ 6.83 (d, $^3J_{HH}$ = 8.5 Hz, 8H), 7.25 (d, $^3J_{HH}$ = 8.5 Hz, 8H);

^{13}C{^{1}H} NMR ($CDCl_3$, 151 MHz, 20 °C): δ 121.42, 131.44, 132.89, 139.74, 141.61.

10e, Tetrabenzo[5.5]fulvalene, orange solid.[S30]

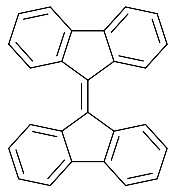

Eluent: Hexane/Ethyl acetate 7/1 (v/v), R_f = 0.64. Yield: 79 %.

^{1}H NMR ($CDCl_3$, 600 MHz, 20 °C): δ 7.23 (dt, $^3J_{HH}$ = 7.6 Hz, $^4J_{HH}$ = 1.2 Hz, 4H), 7.34 (dt, $^3J_{HH}$ = 7.4 Hz, $^4J_{HH}$ = 0.97 Hz, 4H), 7.71 (dt, $^3J_{HH}$ = 7.6 Hz, $^4J_{HH}$ = 0.89 Hz, 4H), 8.40 (dt, $^3J_{HH}$ = 7.9 Hz, $^4J_{HH}$ = 0.88 Hz, 4H);

^{13}C{^{1}H} NMR ($CDCl_3$, 151 MHz, 20 °C): δ 120.01, 126.82, 126.97, 129.26, 138.36, 141.11, 141.40.

10f, 5,5'-Bi(10,11-dihydro-5H-dibenzo<a,d>cycloheptenylidene): white solid[S30]

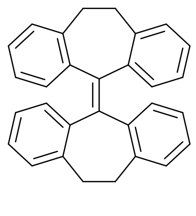

Eluent: Hexane/Ethyl acetate 4/1 (v/v), R_f = 0.59. Yield: 93 %

^{1}H NMR ($CDCl_3$, 600 MHz, 20 °C): δ 3.07 (m, 4H), 3.75 (m, 4H), 6.82 (m, 8H), 7.02 (m, 4H), 7.14 (m, 4H);

^{13}C{^{1}H} NMR ($CDCl_3$, 151 MHz, 20 °C): δ 32.95, 125.55, 126.85, 128.96, 129.25, 138.49, 140.09, 140.96.

11g, 1,2-Bis(2,2-dimethyl-1-phenylpropylidene)hydrazine[S31]

Reaction performed at 60 °C.

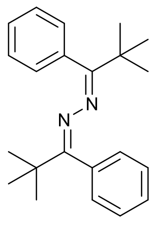

Eluent: Hexane/Ethyl acetate 4/1 (v/v), R_f = 0.84. Yield: 79 %

^{1}H NMR ($CDCl_3$, 600 MHz, 20 °C): δ 0.90 (s, 18H), 7.00 (m, 4H), 7.28 (m, 2H), 7.35 (m, 4H).

^{13}C{^{1}H} NMR ($CDCl_3$, 151 MHz, 20 °C): δ 28.36, 38.10, 127.24, 127.50, 127.61, 137.08, 165.85.

HRMS (ESI): m/z [M+H]$^+$ calculated for $C_{22}H_{28}N_2$: 321.2326; found 321.2352.

Mechanistic investigation

To investigate whether the reaction proceeds via a carbene intermediate, two experiments were conducted, one using styrene and the other cyclopentene as a carbene trap.

Samples were prepared as follows: to a solution of bis(4-methylphenyl) diazomethane **9b** (50 mg, 0.22 mmol) and the carbene trap (0.22 mmol, 1 equiv.; styrene: 23.4 mg, cyclopentene: 15.3 mg) in $CDCl_3$ (0.4 mL) in Young NMR tube was added **3** (1.5 mg, 0.0023 mmol, 1 mol %) in $CDCl_3$ (0.1 mL). The reaction progress was monitored by ^1H NMR at 20 °C and is shown in Figure S37 (styrene) and Figure S38 (cyclopentene).

When the reaction involving styrene proceeded as usual, only forming the expected TPE **10b** product while styrene was left unreacted, cyclopentene was tested instead of styrene, with its double bond expected to be more reactive because of the ring tension. However, no product of the addition on the double bond was observed in this case either. Both experiments confirmed that no free carbene intermediate is generated during the reaction.

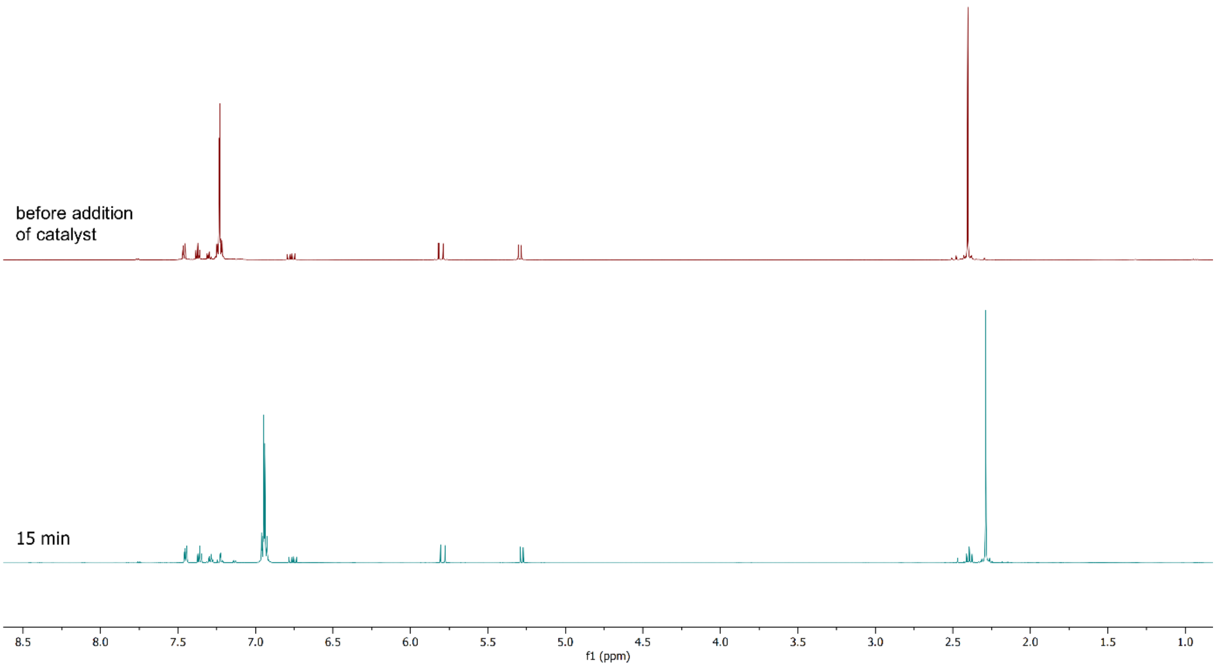

Figure S37: ^1H NMR spectra of the reaction mixture containing bis(4-methylphenyl) diazomethane and styrene in $CDCl_3$ before and 15 minutes after the addition of catalyst **3** (1 mol %) at 20 °C. Only the expected TPE product **10b** is observed, with styrene remaining unreacted, consistent with the absence of a free carbene intermediate.

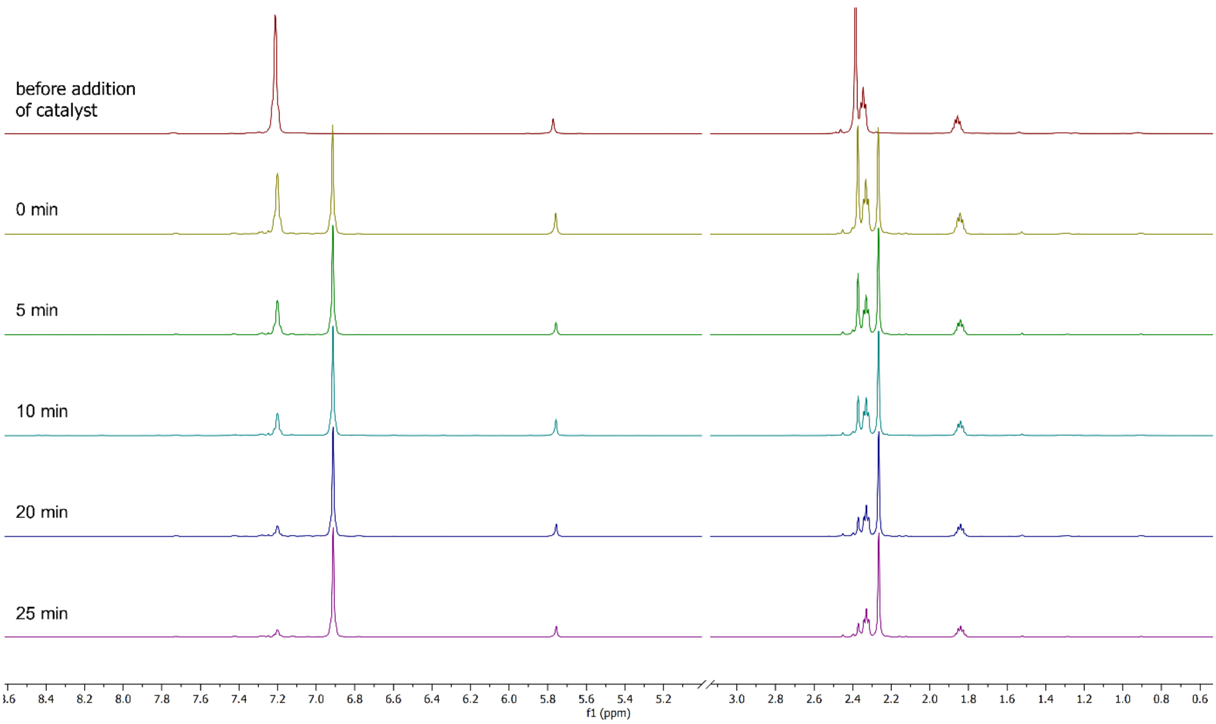

Figure S38: ^{1}H NMR spectra of the reaction mixture containing bis(4-methylphenyl) diazomethane **9b** and cyclopentene in $CDCl_3$ before and after the addition of catalyst **3** (1 mol %) at 20 °C. No evidence of addition to the strained double bond is observed, and the reaction proceeds as usual, forming product **10b**, indicating that no free carbene intermediate is generated.

Cross experiments

9b + 9c experiment

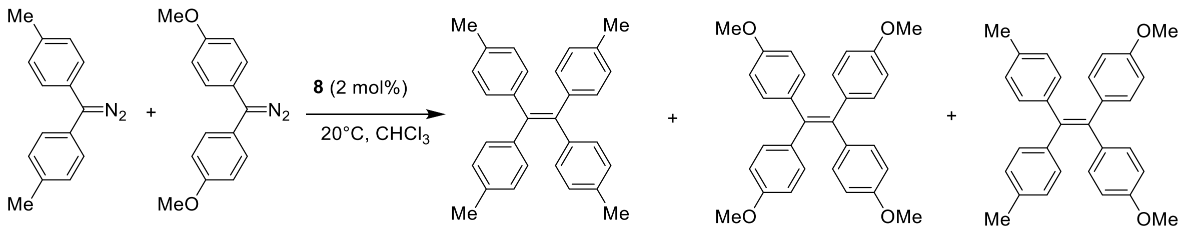

9b **9c** **10b** **10c** **10bc**

To a solution of bis(4-methylphenyl)diazomethane **9b** (49.9 mg, 0.225 mmol, 1 equiv.) and bis(4-methoxyphenyl)diazomethane **9c** (57.1 mg, 0.225 mmol, 1 equiv.) in $CHCl_3$ (5 mL) was added catalyst **8** (4.9 mg, 0.0045 mmol, 2 mol %) in $CHCl_3$ (2 mL) and the reaction was stirred at room temperature overnight. After evaporation of the solvent, reaction products were separated by column chromatography on silica gel using Hexane/Ethyl acetate 4/1 (v/v) as mobile phase.

10b product: R_f 0.88, yield 34.1 mg (0.088 mmol, 78 % of theoretical yield)

10c product: R_f 0.68, yield 28.7 mg (0.063 mmol, 56 % of theoretical yield)

Mixed product 10bc product: R_f 0.70, yield 20.6 mg (0.049 mmol, 21 % of theoretical yield)

Characterization data for **10bc:**

NMR ($CDCl_3$, 20 °C): ^{1}H (600 MHz): δ 2.25 (s, 6H), 3.74 (s, 6H), 6.63 (d, $^3J_{HH}$ = 8.8 Hz, 4H), 6.89 (s, 8H), 6.93 (d, $^3J_{HH}$ = 8.8 Hz, 4H);

^{13}C (151 MHz): δ 21.3, 55.2, 113.1, 128.5, 129.1, 130.4, 131.4, 132.7, 135.7, 136.9, 141.6, 158.0.

Obtained spectra are in accordance with data from literature.[S32]

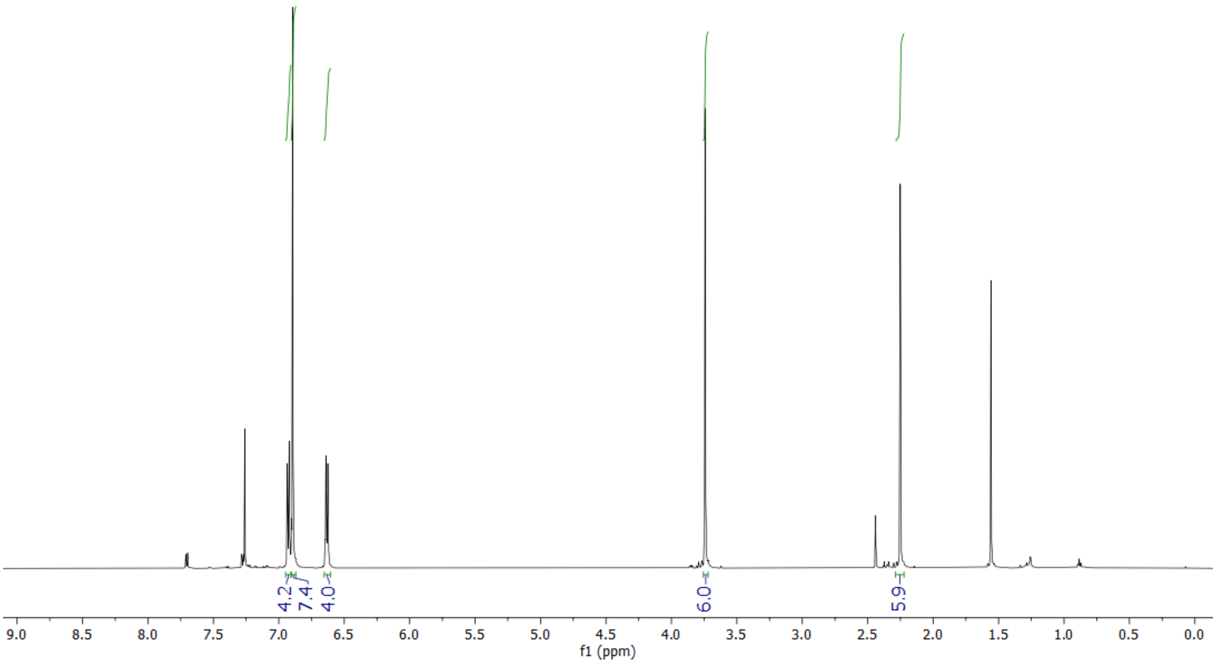

Figure S39: ^{1}H NMR (600 MHz, $CDCl_3$, 20 °C) spectrum of mixed TPE product **10bc**.

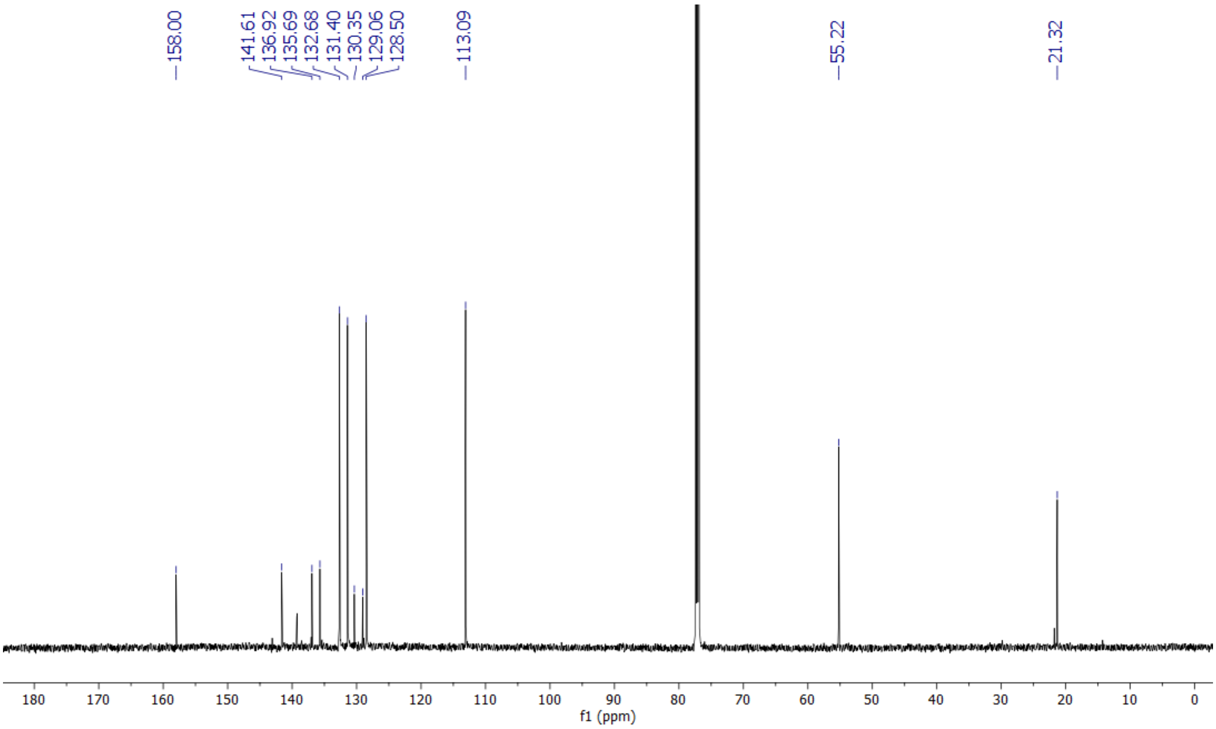

Figure S40: ^{13}C NMR (151 MHz, $CDCl_3$, 20 °C) spectrum of mixed TPE product **10bc**.

2. 9b+9d

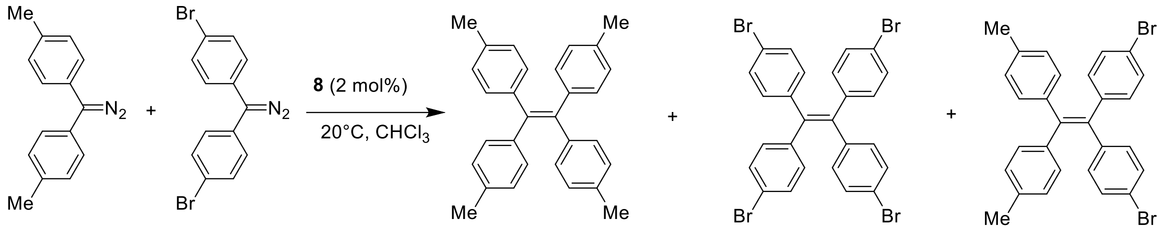

9b **9d** **10b** *not observed* **10d** *not observed* **10bd**

To a solution of bis(4-methylphenyl)diazomethane **9b** (15.8 mg, 0.071 mmol, 1 equiv.) and bis(4-bromophenyl)diazomethane **9d** (25.0 mg, 0.071 mmol, 1 equiv.) in $CDCl_3$ (0.4 mL) in Young NMR tube was added catalyst **8** (1.4 mg, 0.0014 mmol, 1 mol %) in $CDCl_3$ (0.1 mL). Gas evolution was observed immediately and ^{1}H NMR confirmed the formation of the tetrakis(4-methylphenyl)ethylene product **10b** with complete conversion in 15 minutes. The bromide diazo compound **9d** remained unchanged in this time range. No formation of the assumed cross product was observed.

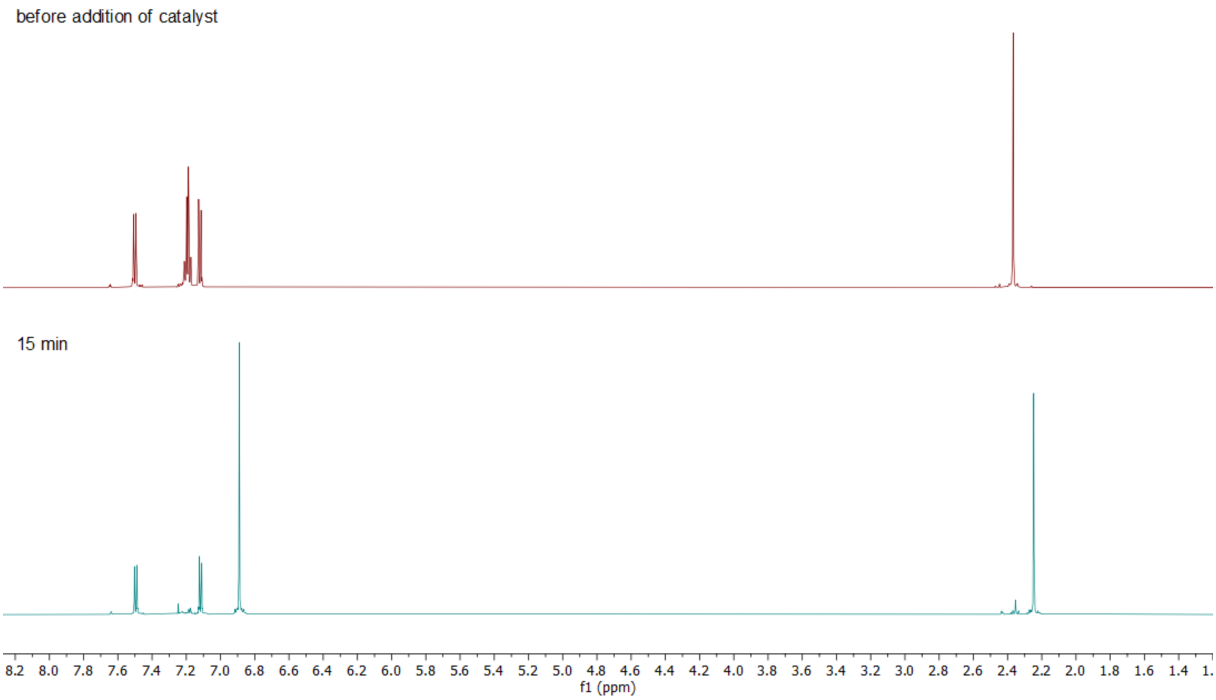

Figure S41: ^{1}H NMR (600 MHz, $CDCl_3$, 20 °C) spectra of the mixture of **9b** and **9d** before and 15 minutes after the addition of the catalyst, showing only product **10b**.

NMR spectra of isolated catalytic products:

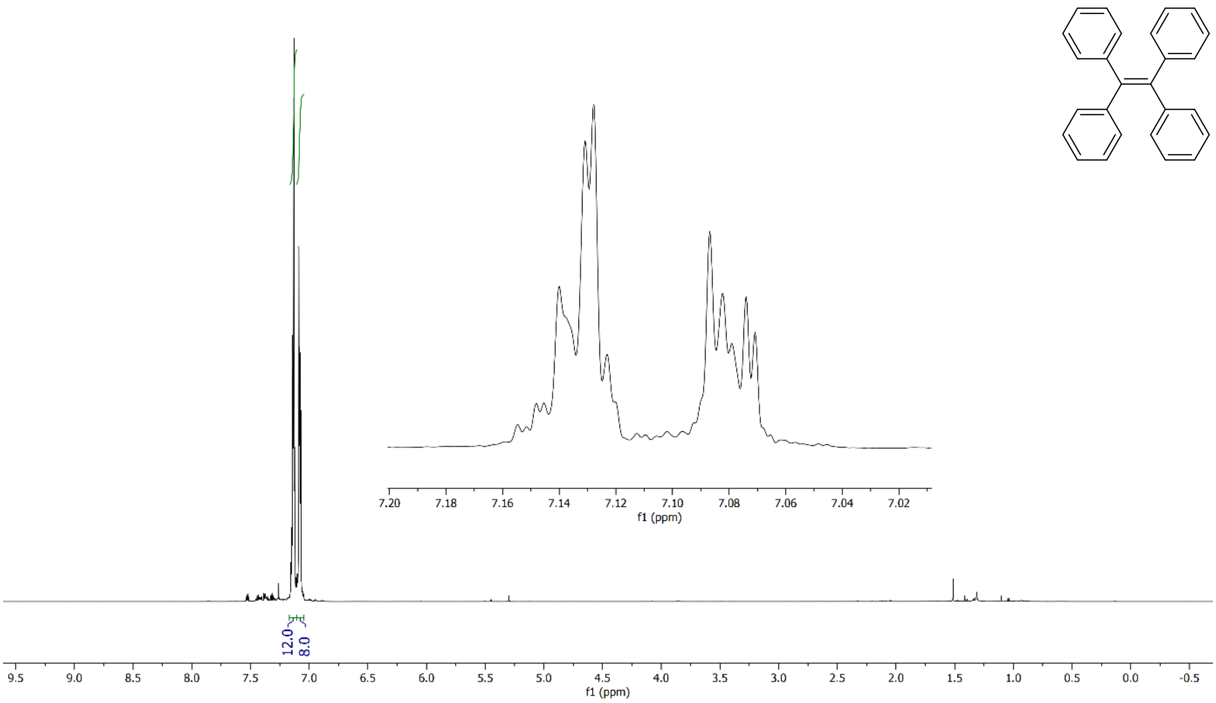

Figure S42: ^{1}H NMR (600 MHz, $CDCl_3$, 20 °C) spectrum of product **10a**.

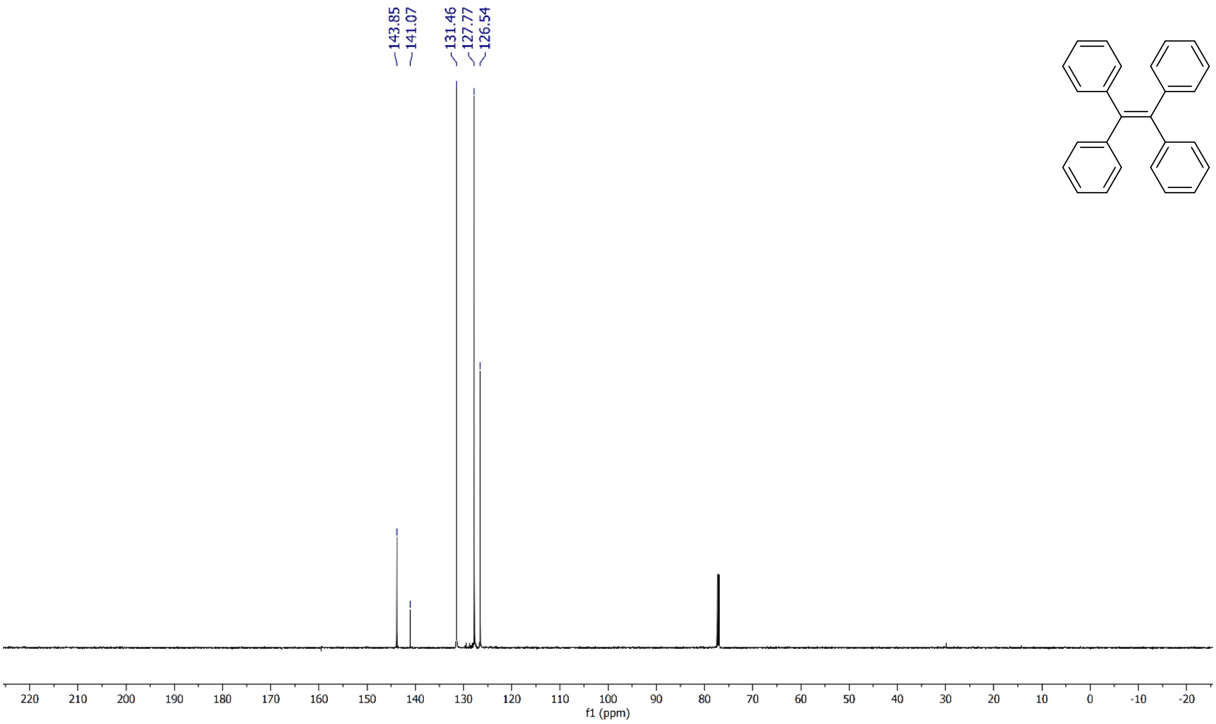

Figure S43: ^{13}C NMR (151 MHz, $CDCl_3$, 20 °C) spectrum of product **10a**.

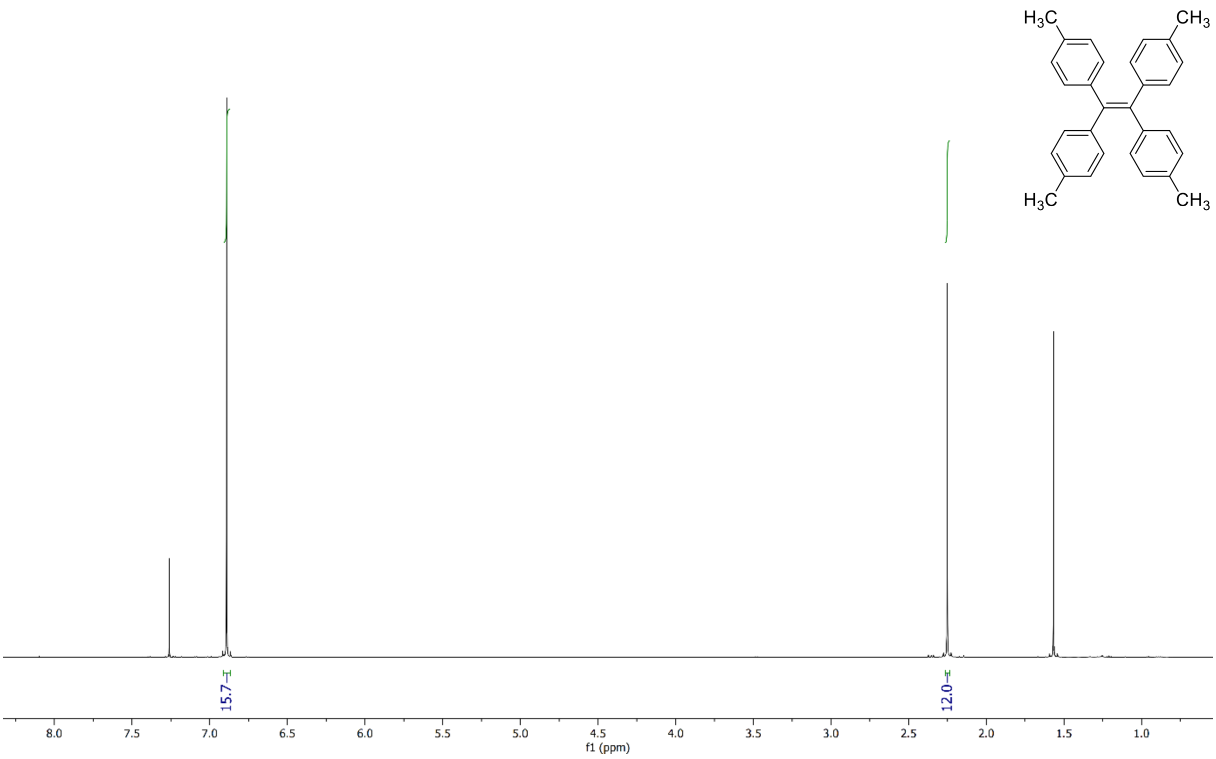

Figure S44: ^{1}H NMR (600 MHz, $CDCl_3$, 20 °C) spectrum of product **10b**.

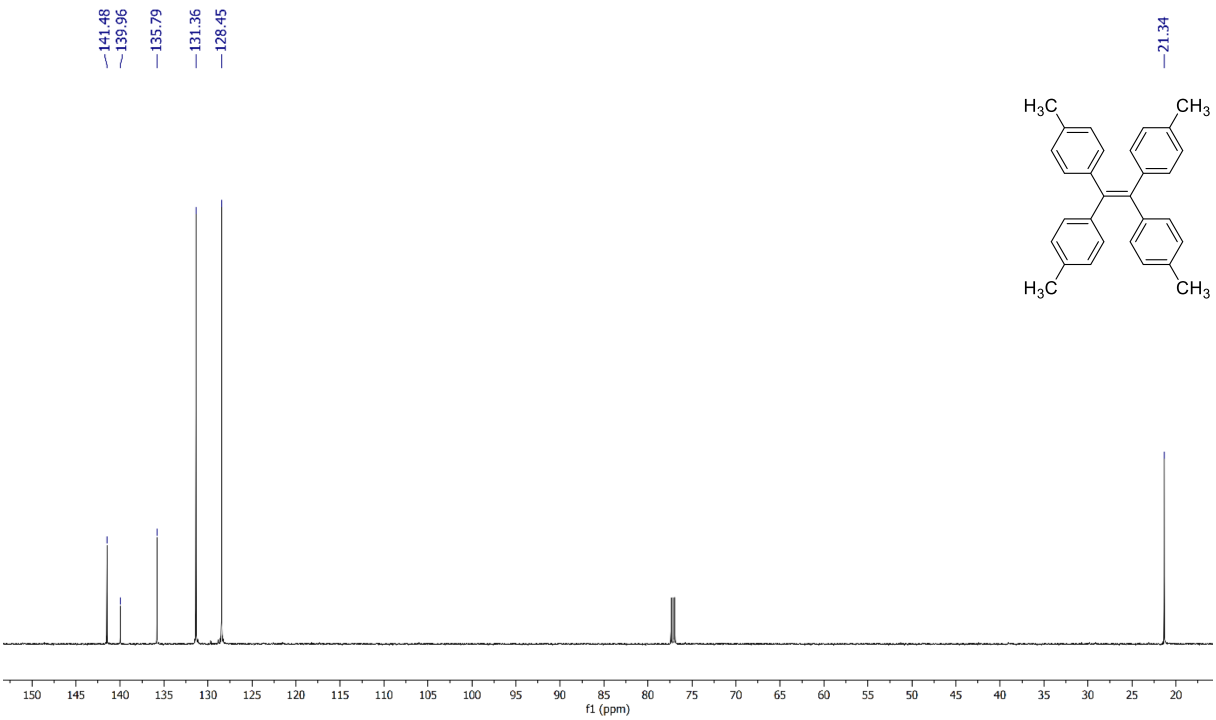

Figure S45: ^{13}C NMR (151 MHz, $CDCl_3$, 20 °C) spectrum of product **10b**.

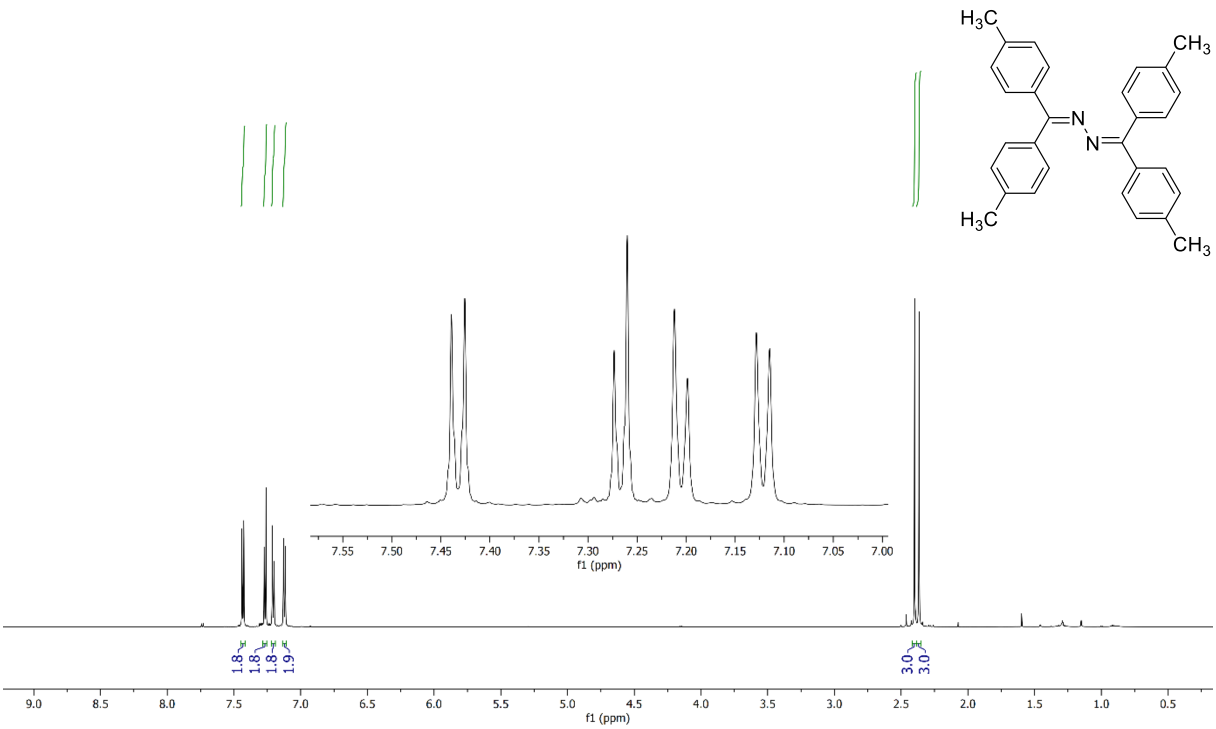

Figure S46: ^{1}H NMR (600 MHz, $CDCl_3$, 20 °C) spectrum of product **11b**.

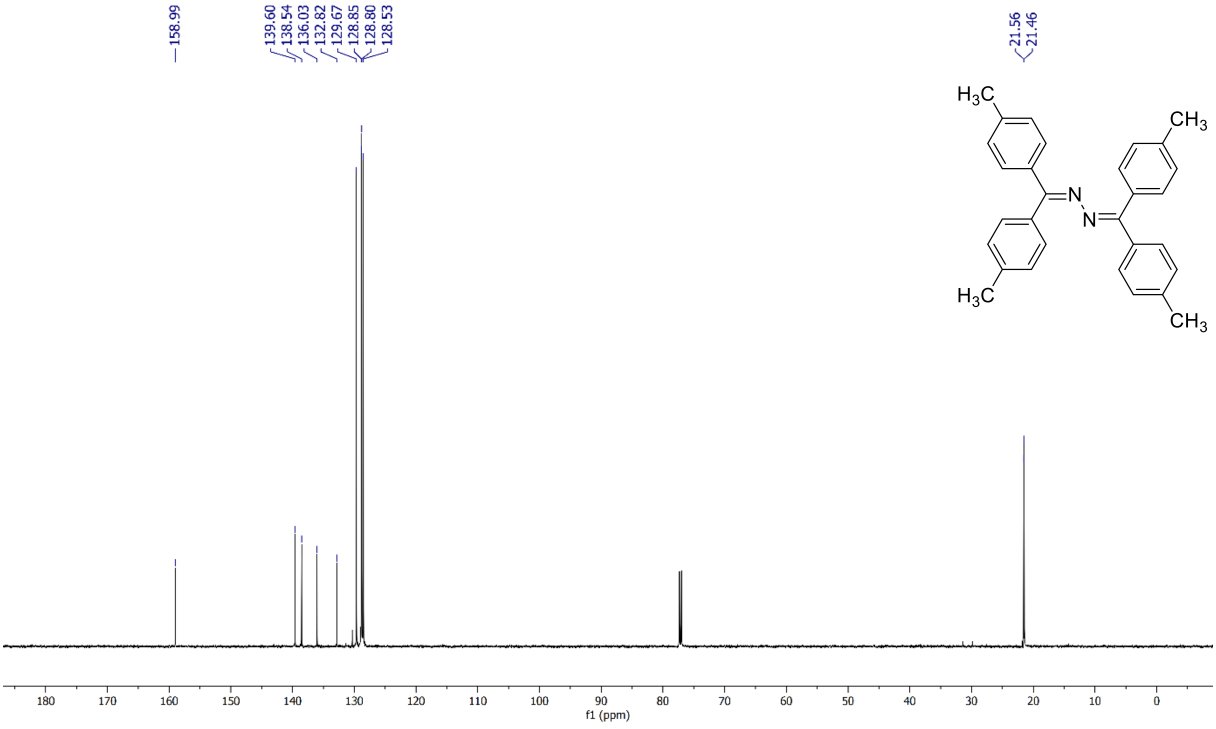

Figure S47: ^{13}C NMR (151 MHz, $CDCl_3$, 20 °C) spectrum of product **11b**.

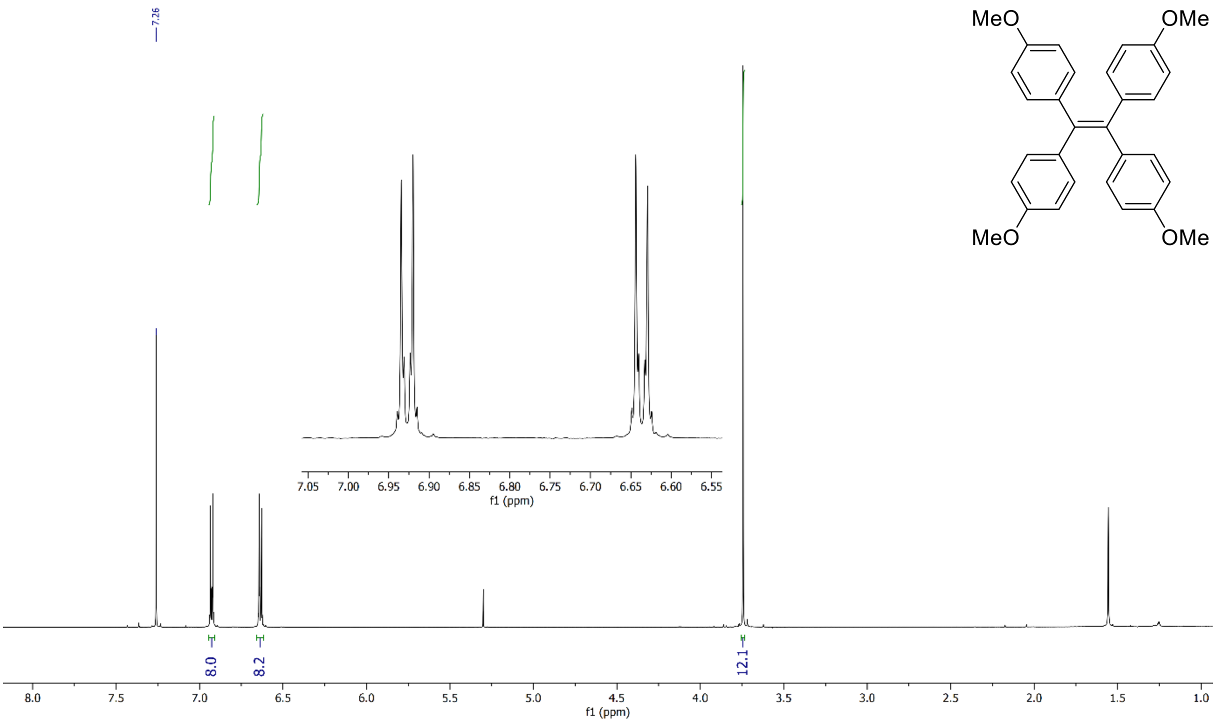

Figure S48: ^{1}H NMR (600 MHz, $CDCl_3$, 20 °C) spectrum of product **10c**.

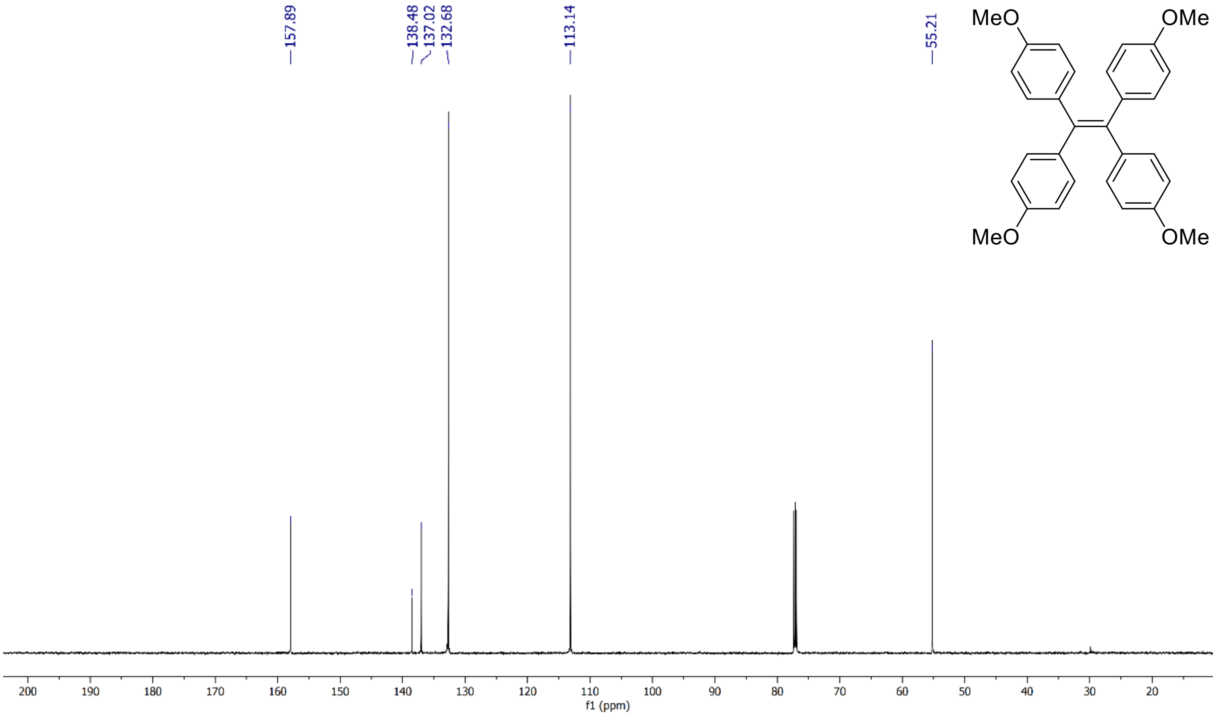

Figure S49: ^{13}C NMR (151 MHz, $CDCl_3$, 20 °C) spectrum of product **10c**.

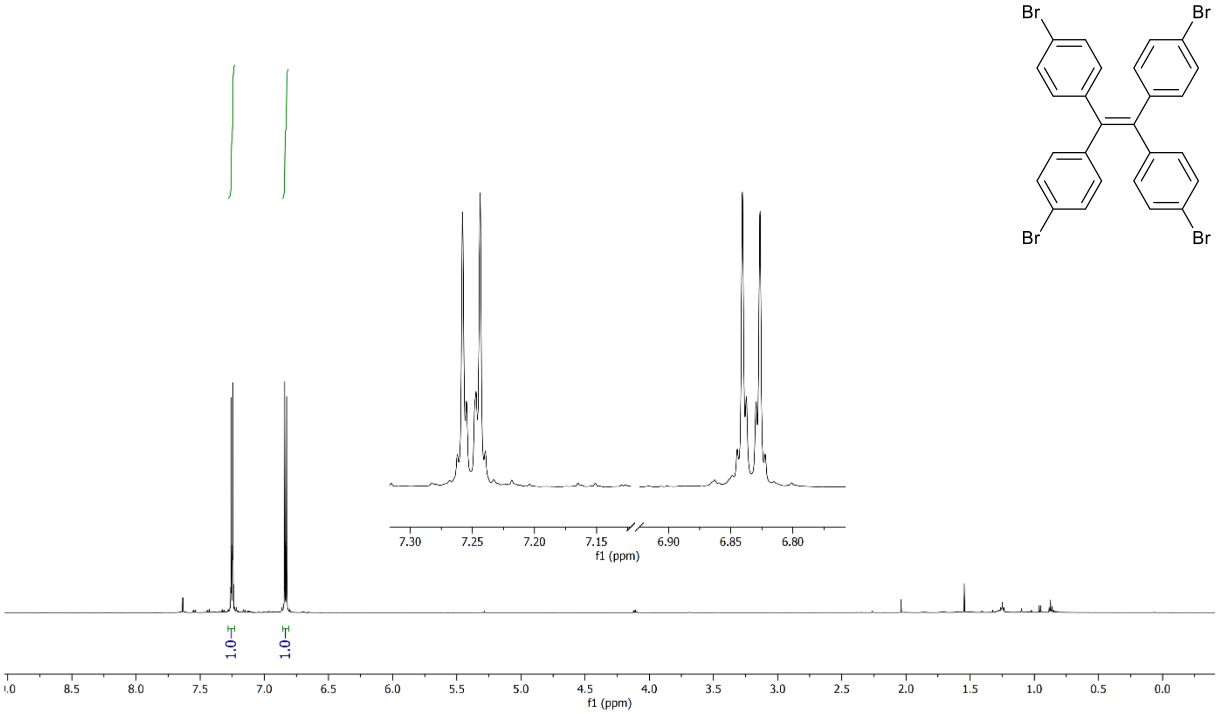

Figure S50: ^{1}H NMR (600 MHz, $CDCl_3$, 20 °C) spectrum of product **10d**.

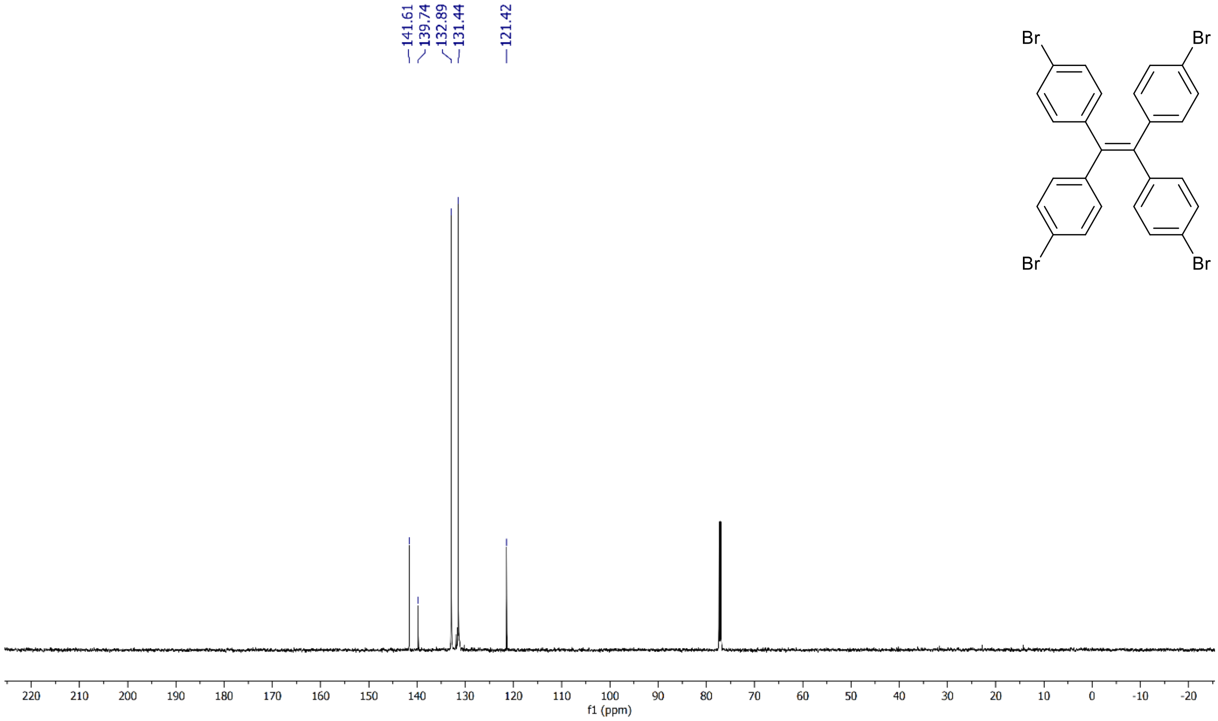

Figure S51: ^{13}C NMR (151 MHz, $CDCl_3$, 20 °C) spectrum of product **10d**.

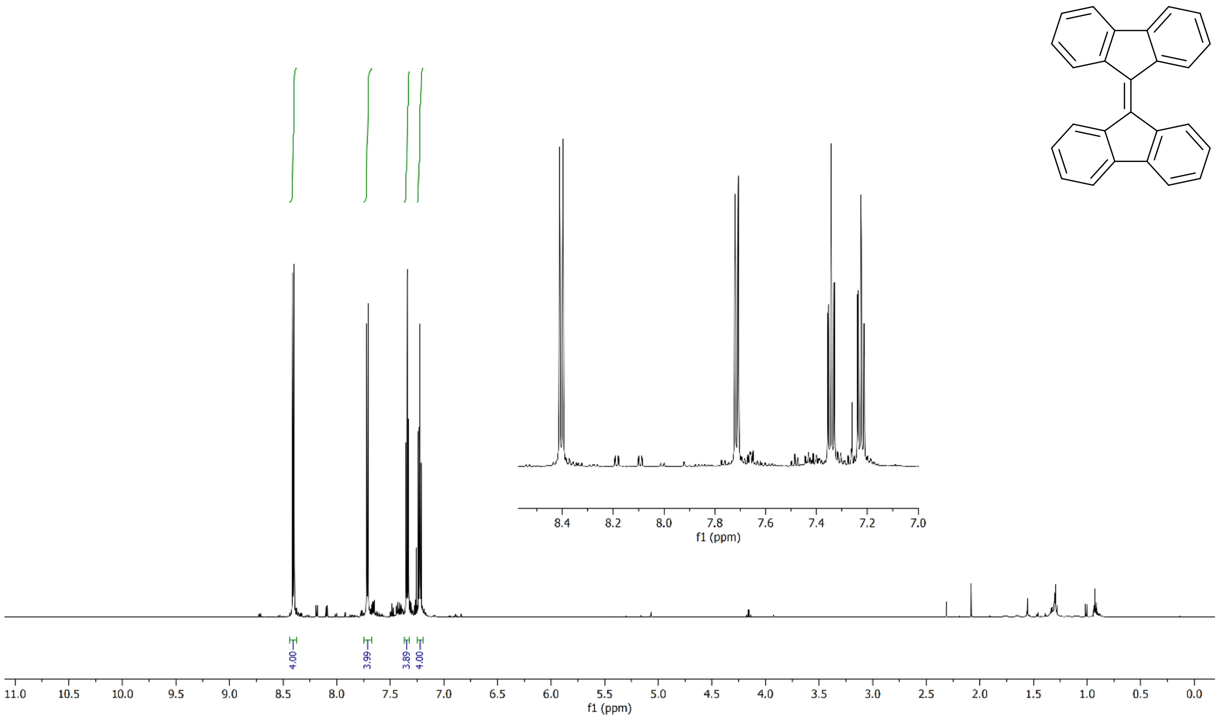

Figure S52: ^{1}H NMR (600 MHz, $CDCl_3$, 20 °C) spectrum of product **10e**.

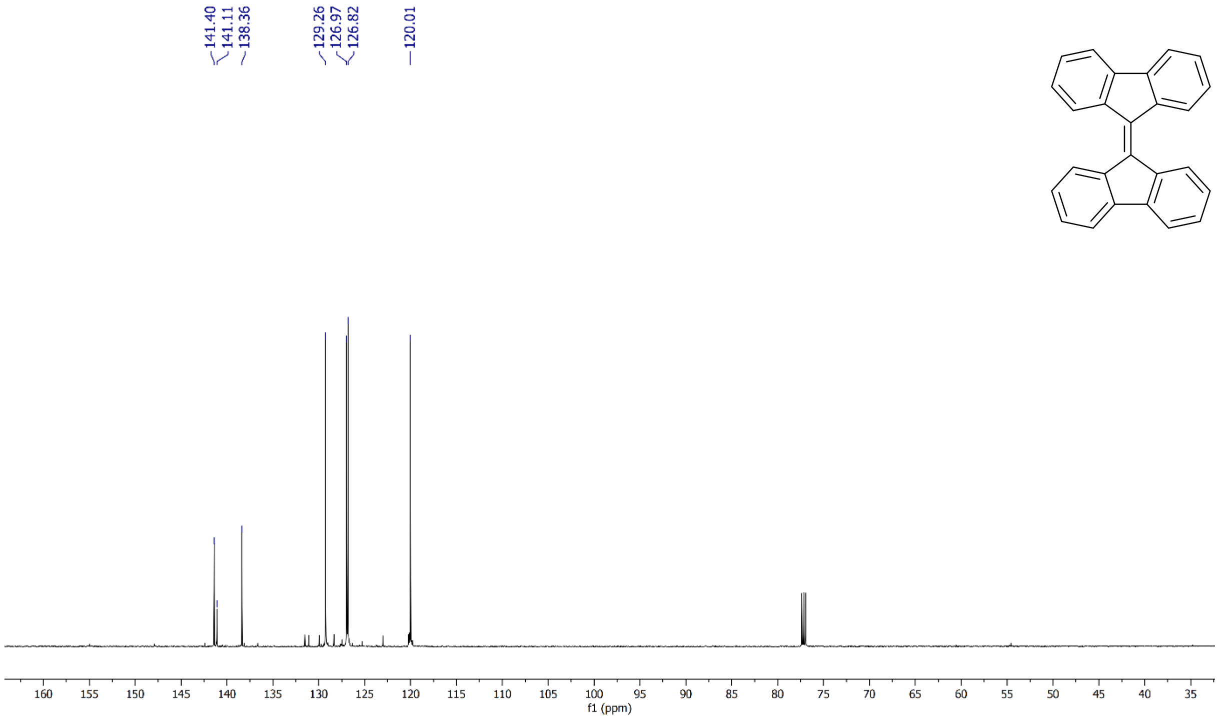

Figure S53: ^{13}C NMR (151 MHz, $CDCl_3$, 20 °C) spectrum of product **10e**.

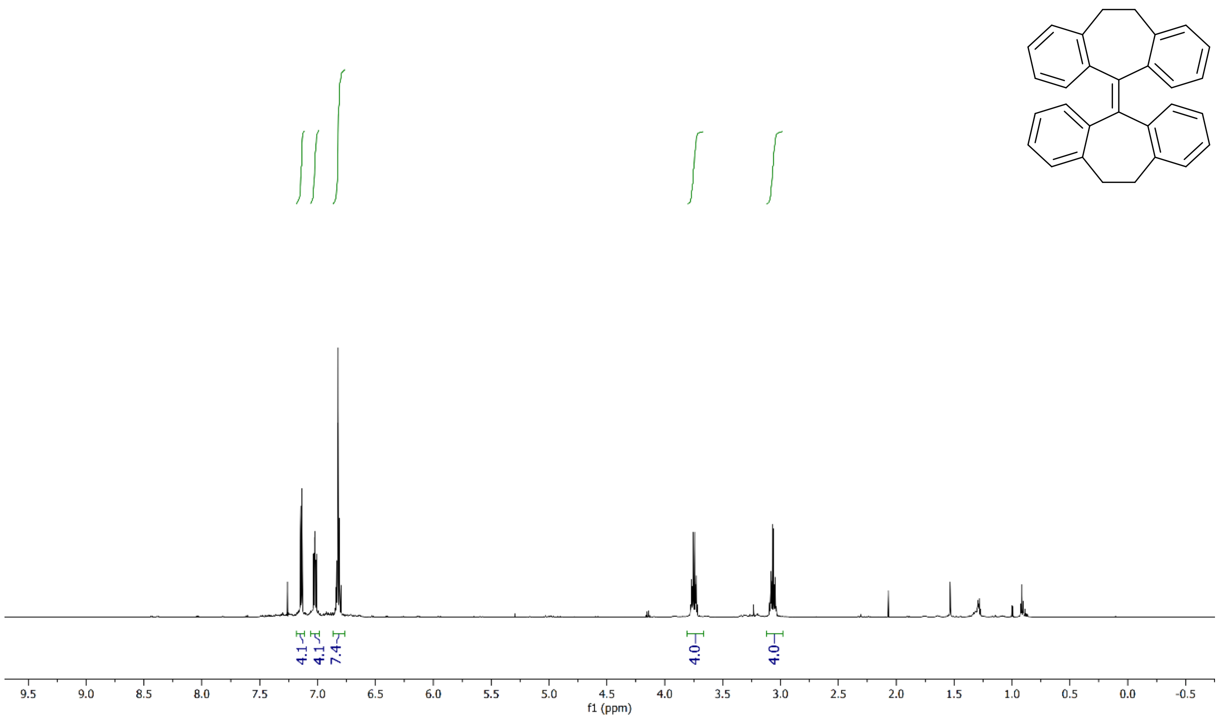

Figure S54: ^{1}H NMR (600 MHz, $CDCl_3$, 20 °C) spectrum of product **10f**.

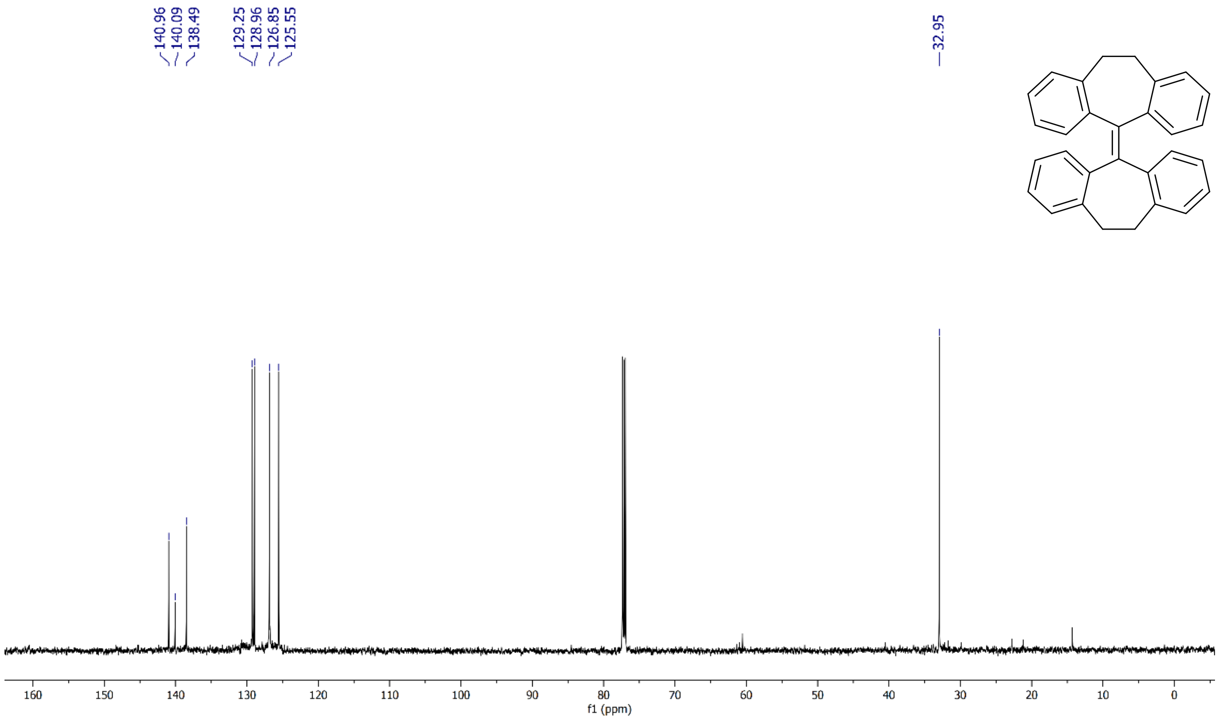

Figure S55: ^{13}C NMR (151 MHz, $CDCl_3$, 20 °C) spectrum of product **10f**.

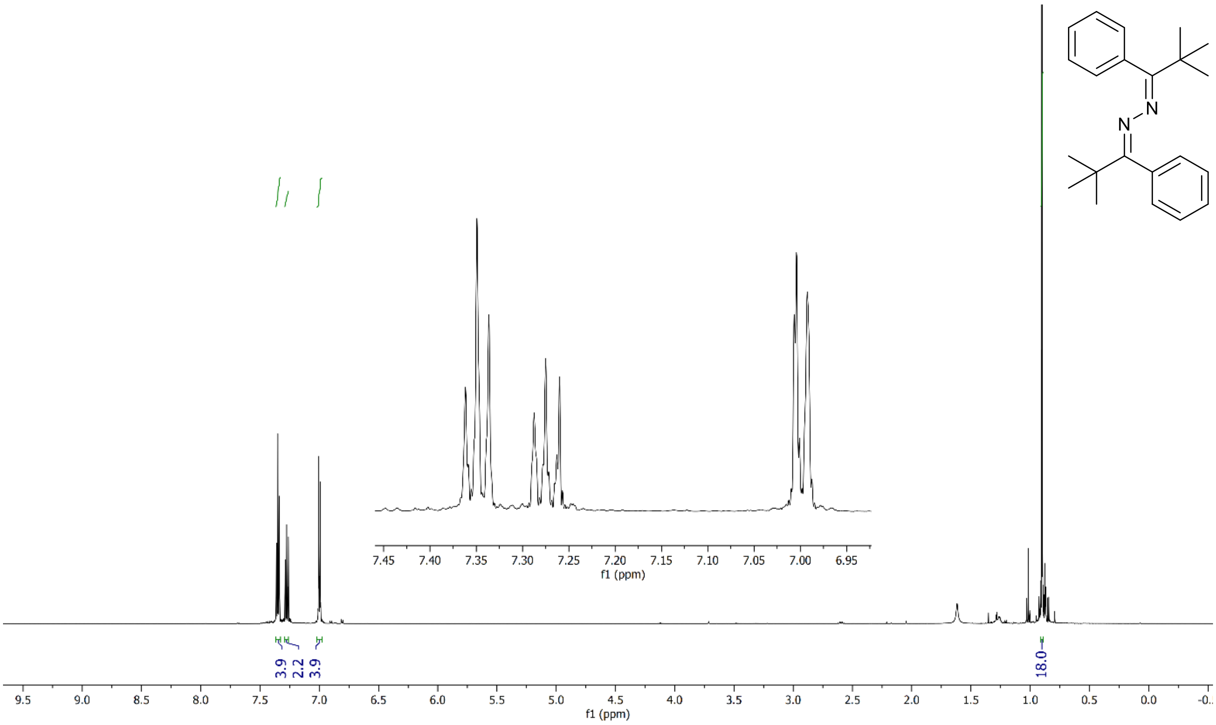

Figure S56: ^{1}H NMR (600 MHz, $CDCl_3$, 20 °C) spectrum of product **11g**.

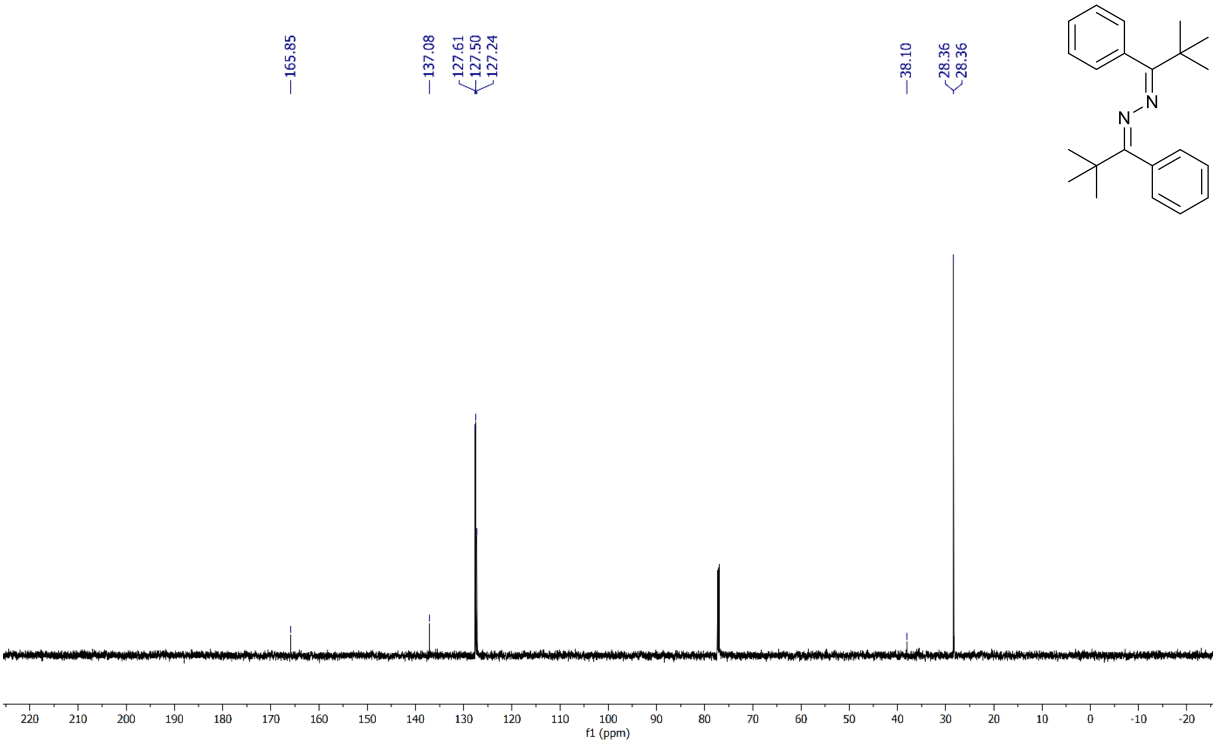

Figure S57: ^{13}C NMR (151 MHz, $CDCl_3$, 20 °C) spectrum of product **11g**.

References – Supplementary Information

S1 Škoch, K.; Buziková, M.; Hnyk, D.; Litecká, M.; Kloda, M.; Kirakci, K.; Lang, K. Preparation, Structure, Reactivity, Lewis Acidic and Fluorescence Properties of Arylpyridine Based Boron C,N-Chelates Featuring Weakly Coordinating Anions, *Chem. Eur. J.* **2024**, *30*, e202403263.

S2 Rigaku OD. (2023). *CrysAlisPro*. Retrieved from https://rigaku.com/products/crystallography/x-ray-diffraction/crysalispro

S3 Coppens, P.; Leiserowitz, L.; Rabinovich, D. Calculation of absorption corrections for camera and diffractometer data, *Acta Cryst.* **1965**, *18*, 1035-1038.

S4 Sheldrick, G.M. SHELXT - Integrated space-group and crystal-structure determination, *Acta Cryst.* **2015**, *A71*, 3-8.

S5 Sheldrick, G.M. Crystal structure refinement with SHELXL, *Acta Cryst.*, **2015**, *C71*, 3-8.

S6 Dolomanov, O.V.; Bourhis, L.J.; Gildea, R.J.; Howard, J.A.K.; Puschmann, H. OLEX2: a complete structure solution, refinement and analysis program, *J. Appl. Cryst.*, 2009, **42**, 339-341.

S7 Diamond, v4.6.3. (2020). *Diamond—Crystal and Molecular Structure Visualization*. Crystal Impact. http://www.crystalimpact.com/diamond

S8 Frisch, M. J.; Trucks, G. W.; Schlegel, H. B.; et al. *Gaussian 16 Revision C.01*, Gaussian Inc., Wallingford CT, 2016.

S9 (a) Perdew, J. P.; Burke, K.; Ernzerhof, M. Generalized Gradient Approximation Made Simple, *Phys. Rev. Lett.* **1996**, *77*, 3865; (b) Perdew, J. P.; Ernzerhof, M.; Burke, K. Rationale for mixing exact exchange with density functional approximations, *J. Chem. Phys.* **1996**, *105*, 9982-9985; (c) Ernzerhof, M.; Scuseria, G. E. Assessment of the Perdew–Burke–Ernzerhof exchange-correlation functional, *J. Chem. Phys.* **1999**, *110*, 5029-5029; (d) Adamo, C.; Barone, V. Toward reliable density functional methods without adjustable parameters: The PBE0 model, *J. Chem. Phys.* **1999**, *110*, 6158-6170.

S10 (a) Weigend, F.; Furche, F.; Ahlrichs, R. Gaussian basis sets of quadruple zeta valence quality for atoms H–Kr, *J. Chem. Phys.* **2003**, *119*, 12753-12762; (b) Weigend, F.; Ahlrichs, R. Balanced basis sets of split valence, triple zeta valence and quadruple zeta valence quality for H to Rn: Design and assessment of accuracy, *Phys. Chem. Chem. Phys.* **2005**, *7*, 3297-3305.

S11 (a) Becke, A. D.; Johnson, E. R. A density-functional model of the dispersion interaction, *J. Chem. Phys.* **2005**, *123*, 154101; (b) Grimme, S.; Antony, J.; Ehrlich, S.; Krieg, H. A consistent and accurate ab initio parametrization of density functional dispersion correction (DFT-D) for the 94 elements H-Pu, *J. Chem. Phys.* **2010**, *132*, 154104; (c) Grimme, S; Ehrlich, S.; Goerigk, L. Effect of the damping function in dispersion corrected density functional theory, *J. Comput. Chem.* **2011**, *32*, 1456-1465.

S12 (a) Tomasi, J.; Mennucci, B.; Cammi, R. Quantum Mechanical Continuum Solvation Models, *Chem. Rev.* **2005**, *105*, 2999–3094; (b) Scalmani, G.; Frisch, M. J. Continuous surface charge polarizable continuum models of solvation. I. General formalism, *J. Chem. Phys.* **2010**, *132*, 114110.

S13 Erdmann, P.; Leitner, J.; Schwarz, J.; Greb, L. An Extensive Set of Accurate Fluoride Ion Affinities for p-Block Element Lewis Acids and Basic Design Principles for Strong Fluoride Ion Acceptors, *ChemPhysChem* **2020**, *21*, 987-994.

S14 Falivene, L.; Cao, Z.; Petta, A.; Serra, L.; Poater, A.; Oliva, R.; Scarano, V.; Cavallo, T. Towards the online computer-aided design o catalytic pockets, *Nat. Chem.* **2019**, *11*, 872-879.

S15 Geertsema, E.M.; Koumura, N.; ter Wiel, M.K.J; Meetsma, A, Feinga, B.L. In control of the speed of rotation in molecular motors. Unexpected retardation of rotary motion, *Chem. Commun.* **2002**, 2962-2963.

S16 Guan, J.; Wei, R.; Prlj, A.; Peng, J.; Lin, K-H.; Liu, J.; Han, H.; Corminboeuf, C.; Zhao, D.; Yu, V; Zheng, J. Direct Observation of Aggregation-Induced Emission Mechanism, *Angew. Chem. Int. Ed.* **2020**, *59*, 14903-14909.

S17 Piteša, T.; Alešković, M.; Becker, K.; Bararić, N.; Došlić, N. Photoelimination of Nitrogen from Diazoalkanes: Involvement of Higher Excited Singlet States in the Carbene Formation, *J. Am. Chem. Soc.* **2020**, *142*, 9718-9724.

S18 Yang, Y.; Han. L.; Taponard, A.; Khrouz, L.; Bucher, C.; Monnereau, C.; Médebielle, M.; Tlili, A. A Shelf-Stable Reagent for Photocatalytic Radical Pentafluorosulfanylation of Styrene Derivatives, *Angew. Chem. Int. Ed.* **2025**, *64*, e202505146.

S19 Zhu, X.; Yang, L.; Pan, Y.; Yang, Y.; Ding, X.; Wan, C.; Zhuo, Z.; Luo, Y.; Zhou, Q.; Wang, L.; Xiao, S. A Three-Dimensional Non-Fullerene Acceptor with Contorted Hexabenzocoronene and Perylenediimide for Organic Solar Cells, *Chem. Eur. J.* **2024**, *30*, e202304167.

S20 Davis, P.J.; Harris, L.; Karim, A.; Thompson, A.L.; Gilpin, M.; Moloney, M.G.; Pound, M.J.; Thompson, C. Substituted diaryldiazomethanes and diazofluorenes: structure, reactivity and stability, *Tetrahedron Lett.*, **2011**, *52*, 1553-1556.

S21 Zhang, Y-D.; Li, X-Y.; Mo, Q-K; Shi, W-B; Zhao, J-B.; Zhu, S-F. Highly Regioselective Cobalt-Catalyzed Hydroboration of Internal Alkynes, *Angew, Chem. Int. Ed.* **2022**, *61*, e202208473.

S22 Sachse, F.; Schneider, C. Direct Access to 1,3-Oxathiolan-5-ones through (3+2)-Cycloaddition of Thioketones and Acetylenedicarboxylic Acid, *Eur. J. Org. Chem.* **2023**, *26*, e202300834.

S23 Levy, A.; Cohen, S.; Pogodin, S.; Agranat, I. Selenium- and tellurium-bridged heteromerous overcrowded bistricyclic aromatic enes with central six-member and seven-member rings, *Struct. Chem.* **2015**, *26*, 1565-1584.

S24 Celebi, S.; Leyva, S.; Modarelli, D.A.; Platz, M.S. 1,2-Hydrogen migration and alkene formation in the photoexcited states of alkylphenyldiazomethanes, *J. Am. Chem. Soc.* **1993**, *115*, 8613-8620.

S25 Humphreys, R.W.R.; Arnold, D.R. Substituent effects on the triplet–singlet energy separation in diphenylmethylene, *Can. J. Chem.* **1977**, *55*, 2286.

S26 Hazdra, S.; Panda, S. Stereoselective Synthesis of Silylated Vinylboronates by a Boron-Wittig Reaction and Their Application to Tetrasubstituted Olefins, *Chem. Eur. J.* **2024**, *12*, e202303056.

S27 Wang, Y-P; Gup, Z-Z.; Qu, J-P.; Kang, Y-B. Photoreductive N–N Homocoupling Catalyzed by a Superphotoreductant, *J. Org. Chem.* **2024**, *89*, 16804-16808.

S28 Pérez-Márquez, L.A.; Perretti, M.D.; García-Rodríguez, R.; Lahoz, F.; Carrillo, R. *Angew. Chem. Int. Ed.*, **2022**, *61*, e202205403.

S29 Maji, S.; Samanta, J.; Samanta. K., Natarajan, R. A Fluorescent Cage for Supramolecular Sensing of 3-Nitrotyrosine in Human Blood Serum, *Chem. Eur. J.* **2023**, *29*, e202301985.

S30 Purahong, N.; Hongthong, S.; Chotsaeng, N.; Kuhakarn, C; Meesin, J. DBU-Mediated Dimerization: Facile Access to 9,9′-Bifluorenylidenes and Isoindigos, *Synlett* **2023**, *34*, 2323-2328.

S31 Malament, D.S.; McBride, J.M. α,α′-Dichloroazoalkanes. I. Synthesis: Stereospecificity and side reactions. The crystal structure of 1,1'-dichloro-1,1'-diphenyl-1,1'-azopropane, *J. Am. Chem. Soc.* **1970**, *92*, 4586-4593.

S32 Okuma, K.; Kojima, K.; Oyama, K.; Kubo, K.; Shioji, K. Selenobenzophenones and Diazoalkanes: Isolation of Tetraarylethylenes by the Reaction of Benzophenone Hydrazones with Diselenium Dibromide, *Eur. J. Org. Chem.* **2004**, 820-825.
